# dissectHMMER: a HMMER-based score dissection framework that statistically evaluates fold-critical sequence segments for domain fold similarity

**DOI:** 10.1186/s13062-015-0068-3

**Published:** 2015-08-01

**Authors:** Wing-Cheong Wong, Choon-Kong Yap, Birgit Eisenhaber, Frank Eisenhaber

**Affiliations:** Bioinformatics Institute (BII), Agency for Science, Technology and Research (A*STAR), 30 Biopolis Street, #07–01, Matrix, Singapore, 138671 Singapore; Department of Biological Sciences (DBS), National University of Singapore (NUS), 8 Medical Drive, Singapore, 117597 Singapore; School of Computer Engineering (SCE), Nanyang Technological University (NTU), 50 Nanyang Drive, Singapore, 637553 Singapore

**Keywords:** Sequence homology, Hidden Markov model, Sequence similarity search, Fold-critical sequence segment, Non-globular sequence segment, Similarity score dissection

## Abstract

**Background:**

Annotation transfer for function and structure within the sequence homology concept essentially requires protein sequence similarity for the secondary structural blocks forming the fold of a protein. A simplistic similarity approach in the case of non-globular segments (coiled coils, low complexity regions, transmembrane regions, long loops, etc.) is not justified and a pertinent source for mistaken homologies. The latter is either due to positional sequence conservation as a result of a very simple, physically induced pattern or integral sequence properties that are critical for function. Furthermore, against the backdrop that the number of well-studied proteins continues to grow at a slow rate, it necessitates for a search methodology to dive deeper into the sequence similarity space to connect the unknown sequences to the well-studied ones, albeit more distant, for biological function postulations.

**Results:**

Based on our previous work of dissecting the hidden markov model (HMMER) based similarity score into fold-critical and the non-globular contributions to improve homology inference, we propose a framework-dissectHMMER, that identifies more fold-related domain hits from standard HMMER searches. Subsequent statistical stratification of the fold-related hits into cohorts of functionally-related domains allows for the function postulation of the query sequence. Briefly, the technical problems as to how to recognize non-globular parts in the domain model, resolve contradictory HMMER2/HMMER3 results and evaluate fold-related domain hits for homology, are addressed in this work. The framework is benchmarked against a set of SCOP-to-Pfam domain models. Despite being a sequence-to-profile method, dissectHMMER performs favorably against a profile-to-profile based method-HHsuite/HHsearch. Examples of function annotation using dissectHMMER, including the function discovery of an uncharacterized membrane protein Q9K8K1_BACHD (WP_010899149.1) as a lactose/H+ symporter, are presented. Finally, dissectHMMER webserver is made publicly available at http://dissecthmmer.bii.a-star.edu.sg.

**Conclusions:**

The proposed framework-dissectHMMER, is faithful to the original inception of the sequence homology concept while improving upon the existing HMMER search tool through the rescue of statistically evaluated false-negative yet fold-related domain hits to the query sequence. Overall, this translates into an opportunity for any novel protein sequence to be functionally characterized.

**Reviewers:**

This article was reviewed by Masanori Arita, Shamil Sunyaev and L. Aravind.

**Electronic supplementary material:**

The online version of this article (doi:10.1186/s13062-015-0068-3) contains supplementary material, which is available to authorized users.

## Background

The sequence homology concept [1–3] is collectively founded upon the inductive reasoning that a homologous protein group (as an antecedent) shares a high level of sequence similarity (as a consequent) [4–8]. Implicitly, this refers to a high level of similarity among comparable structural elements across the sequences so that a common structural fold among these homologs is maintained which, in turn, governs the general biological function of this homologous protein family. In simple terms, the highest abstraction of a biological function is conferred by the protein fold. In turn, fold conservation implies the conservation of a sequence pattern of hydrophilic/hydrophobic and size-restricted residues within the protein family.

Despite the simplicity and elegance of the sequence homology concept, homology itself is not readily computable. Its closest surrogate is the sequence similarity measure. This measure comes along with the caveat of possibly high sequence similarity does not necessarily imply homology. Worse, as we delve deeper into sequence search space, high sequence divergence among the distant homologs will inevitably corrupt the homology signal. Hence, the only recourse to maintain on the correct search path is to piggyback on the similarity between the structural pieces of the alignment to ensure reasonable fold similarity and, hence, the implied biological function.

Regrettably, current implementation of sequence search algorithms do not consciously differentiate between the 3D-structural (i.e., fold-critical) sequence segments and the non-globular (i.e., remnant) segments. As such, statistically significant yet spurious alignments (attributed by the remnant segments) can pass off as homologous sequences once they escape the designated statistical threshold. In mitigation though, mainstream sequence search algorithms like BLAST [9, 10] and HMMER [11, 12] deploy compositional bias statistics [13–15] to suppress some classes of remnant segments (e.g., low-complexity sequence). However, compositional bias statistics is purely a mathematical solution that does not necessarily only target remnant segments. As an example, the low-complexity structural α-helices can be suppressed as well [16]. On top of that, it can also compromise on search sensitivity (true-positive detection) while suppressing the false-positive hits. The latter marks a trade-off.

And given that the number of well-studied proteins continues to grow at a slow rate [17], the transfer of functional annotation from characterized sequences to unknown ones remains important [3]; yet it is rate-limiting. As such, this necessitates for a search methodology that can search deeper into the sequence similarity space to link (via fold similarity) the unknowns to the known ones while staying theoretically truthful to the sequence homology concept.

As fold similarity detection is the cornerstone of homology searches and the fold is defined as the spatial arrangement of secondary structural elements [18], the exclusion of the evolutionarily more variable loop regions and, generally, the excision of non-globular sequence segments should minimize noise in the sequence similarity searches. Essentially, the dissectHMMER approach attempts to make use the basic concept.

The idea of dissecting the similarity score of the sequence-to-domain alignment into its fold-critical (i.e., 3D-structural elements) and remnant (i.e., non-globular elements) sums with subsequent statistical re-evaluation of their E-values was introduced in our recent work [19]. In particular, the HMMER variants (HMMER2 and HMMER3) were investigated. As a necessary condition to be considered as a true hit, the fold-critical E-value should be either be more significant than its remnant E-value, or minimally be statistically significant on itself. As a proof of concept, the score dissection idea has been shown to elucidate previously obscured true hits due to bad E-values attributed by the remnant sequence segments, while suppressing the false hits at the same time. As such, there is no need to compromise for high false-negative rates (low sensitivity) in exchange for low false-positive rates (low specificity). Also, compositional bias statistics to suppress false-hits becomes less important with the score dissection. Interestingly, this was achieved without any modifications to the search algorithm itself, since the score dissection was applied to the alignments post mortem. Essentially, the score dissection idea provides a new paradigm in which homology can be better evaluated with improved search sensitivity/specificity and deeper search depth. Most importantly, it is more faithful to the original inception of the sequence homology concept than current sequence search implementations.

With the necessary proof of concept established in our previous work [19], the current work extends our existing proof to a full-fledged implementation of the score dissection idea, herewith, dissectHMMER (available at dissecthmmer.bii.a-star.edu.sg). In a nutshell, dissectHMMER attempts to break the limits of current sequence search algorithms (whether sequence-to-profile or profile-to-profile based methods) to better bridge between the sequence similarity space and the structural similarity space with its deeper search depth. This is achieved through rescuing the false-negative sequence-to-domain hits by re-capturing the significance of the fold-critical sequence segments of these hits. As outputs, dissectHMMER searches for a set of statistically confident domain hits with similar fold for a given query sequence.

In the course of implementing dissectHMMER, several issues were resolved. Firstly, the annotation of fold-critical residues in domain models is an integral part of the score dissection idea. Previously, for domain models without PDB/DSSP information, the quality-score [20] was used to predict the fold-critical residues in domain models. In hindsight, it was found to underestimate the remnant sequence segments. In the current work, dissectHMMER uses a weighted combination of quality-score [20], PSIPred [21, 22], SEG [23] and GlobPlot [24] to improve the sensitivity/specificity of this prediction task. In the process, a handful of problematic domain models (e.g. domains without fold-critical residues, domains with dominant proportion of remnant residues) were found.

Secondly, the fold-critical E-value and the ratio (i.e. fold-critical E-value/remnant E-value) of a sequence-to-domain alignment are two critical surrogate measures of the fold-critical score in the score dissection idea. Briefly, a confident sequence-to-domain hit is founded on a low fold-critical E-value and a low ratio. However, the ranges of fold-critical E-values and the ratios exhibited by the HMMER variants (i.e. HMMER2 and HMMER3) can differ. Fundamentally, the difference stems from the utilized alignment modes in HMMER2 (i.e., glocal mode; local to sequence, global to domain) and HMMER3 (i.e., local mode; local to both sequence and domain). This is further underpinned by the difference between glocal gapped alignment statistics in HMMER2 and ungapped alignment statistics in HMMER3.

Beyond the different statistical consideration, HMMER3 can only operate in local mode where significant hits are concluded from fragmented sequence-to-domain alignments. Unlike HMMER2, the latter is insufficient for inferring protein domain function since a domain denotes a unit of function. As such, HMMER2 glocal mode remains relevant for domain annotation work and its results can be complemented by HMMER3’s improved sensitivity and specificity that increases search space.

In practice, the difference in the derived E-values by the HMMER variants can nevertheless be problematic for overlapping HMMER2 and HMMER3 sequence-to-domain alignments. At times, contradiction cases can occur, where one HMMER variants declares the hit as true and the other declares it as false.

In the current work, the differences are properly quantified by dissectHMMER where the hits are associated to some expected false-positive rates. These false-positives rates which were sampled from the vicinity of negative domain hits to a set of SCOP-to-Pfam sequences, are employed to correct the fold-critical E-values and ratios accordingly. By doing so, the overlapping (occasionally contradictory) sequence-to-domain results can then be merged in a justified manner after the corrections.

Thirdly, an error-adjusted domain-coverage measure is introduced in dissectHMMER. Since the objective of dissectHMMER is to detect domains with similar fold, this measure accounts for the statistical significance of the domain hits, as well as the coverage of the domain. When benchmarked against a set of SCOP superfamily profiles (i.e. distant homologs with the same protein fold), dissectHMMER was found to perform favourably against its former self, HMMER and a profile-to-profile method-HHsuite/HHsearch.

Finally, through several case studies of elucidating more fold-related domain hits through a deeper search depth and subsequently stratifying the quantified hits into functionally-related domain cohorts, dissectHMMER has demonstrated its ability to minimally propose a generalized function when combined with biological evidence. The latter is crucial for many novel sequences whose current search space cannot be linked to any well-characterized protein sequences. In addition, the stratification of the quantified domain hits (via the ordered total FPR) helps to guide the amount or level of function transfer from the most significant domain hit to the sequence, depending on the magnitude of the total FPR. As such, the dissectHMMER framework attempts to balance between over-and under-prediction of biological function while presenting an opportunity for the currently novel protein sequences to be functionally characterized as exemplified by the novel sequence Q9K8K1_BACHD (WP_010899149.1) in our case study.

## Results and discussion

### The HMMER score dissection framework: dissectHMMER webserver

**Fig. 1:**
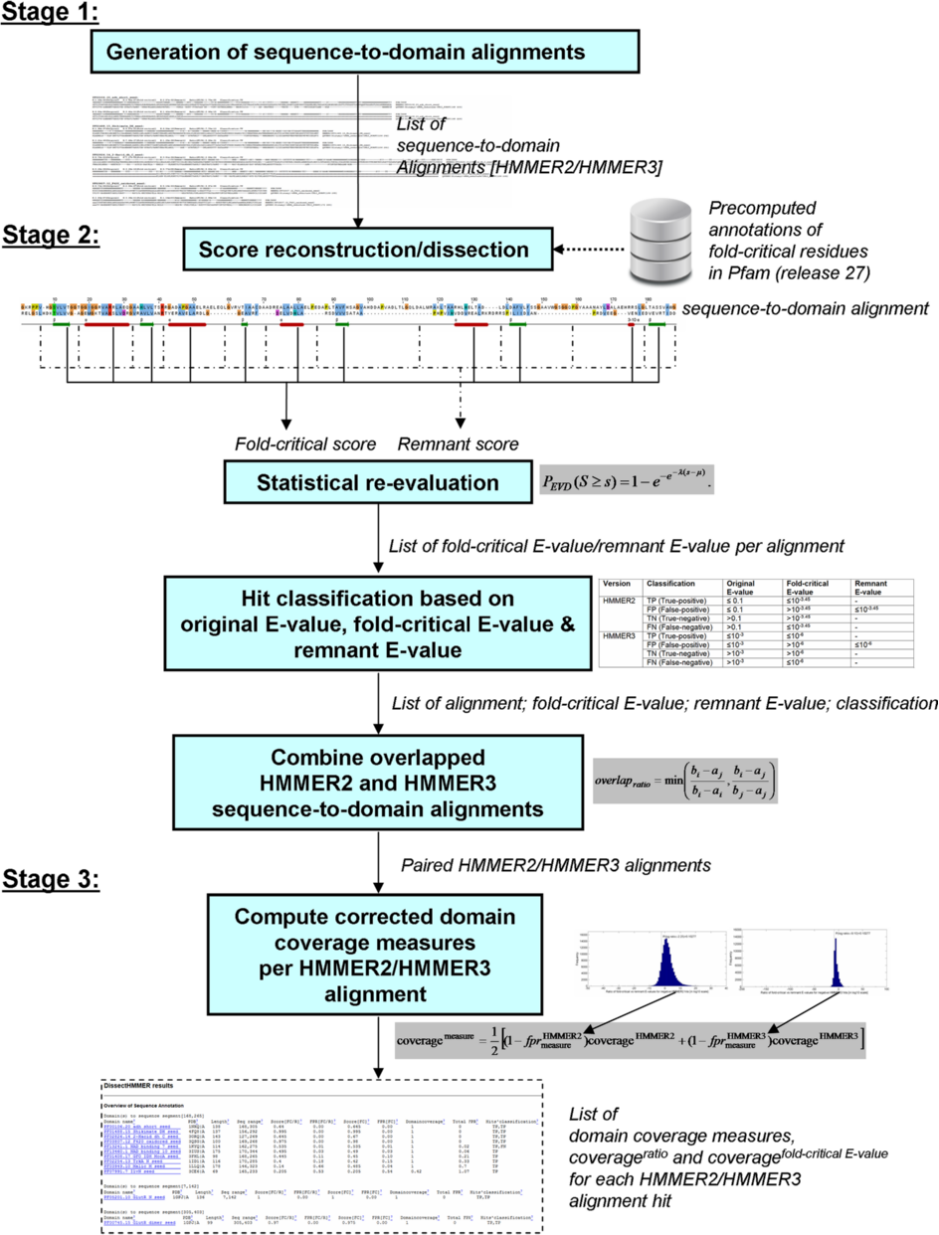
The HMMER score dissection framework, herewith, dissectHMMER is implemented in Perl and resides at dissecthmmer.bii.a-star.edu.sg. The workflow of dissectHMMER is generalized into three stages: 1) Generation of the sequence-to-domain alignment 2) score reconstruction/dissection/statistical re-evaluation/hits classification and 3) Computations of the error-adjusted domain coverage measures

The HMMER score dissection framework, herewith, dissectHMMER is implemented in Perl and resides at dissecthmmer.bii.a-star.edu.sg. As depicted in Fig. 1, the dissectHMMER workflow can be generalized into three stages: 1) sequence-to-domain alignment generation, 2) score reconstruction/dissection/statistical re-evaluation/hits classification and 3) error-adjusted domain coverage computation. It must be emphasized that no algorithmic changes are necessary to the original HMMER codes since the main computations in dissectHMMER is done after the alignments have been generated.

In the sequence-to-domain alignment generation stage, both HMMER2 (in glocal mode) and HMMER3 (limited to local mode) were used concurrently to generate the alignment results when presented with a query sequence. In the current setup, the glocal mode for HMMER2 is enforced so that a full alignment with respect to the domain model can be made with the query sequence to maximize the belief of the domain’s overall fold similarity when the hit is subsequently evaluated to be true. In contrast, HMMER3 by itself cannot always guarantee the full alignment with respect to the domain model. As such, this can lead to fragmented sequence-to-domain alignments that suggest only partial domain fold similarity. In hindsight, the overall fold similarity to a domain is a necessary condition for inferring its biological function since the ideal notion of protein domain necessarily encompasses an unit of function by definition.

Nevertheless, the inclusion of HMMER3 [12] was necessary since its sensitivity and specificity was supposedly improved over HMMER2 [11] other than its computational speed. Aside that, the average domain model length in the current Pfam library (release 27) is about 230 with a standard deviation of approximately 200. As such, about 12 % (1795 out of 14,831) of the Pfam domain models are longer than 430 in length and hence likely to be multi-domain. For these multi-domain Pfam models, HMMER3 has a higher likelihood of capturing the individual domains as “fragmented” hits than HMMER2 in glocal mode. Whenever possible, the common or overlapping sequence-to-domain alignment that arose from both HMMER2 and HMMER3 are paired at an overlap ratio, *overlap*_*ratio*_ of 0.9 (see Eq. 4 in Methods). Here, overlap refers to the common sequence coverage by the same domain model between two sequence-to-domain alignments.

In the score reconstruction/dissection/statistical re-evaluation/hits classification stage, the full score of each alignment is reconstructed using the emission/transition/invariant log-odd scores of the alignment’s domain model. Then, this is followed by the score dissection computations. Meanwhile, a crucial component that is tightly coupled to the dissection, is the predefined positions of the fold-critical and remnant residues in each of the Pfam [25, 26] domain models. These annotations are derived from the PDB/DSSP information for 6599 Pfam models with representative structures while the remaining (8232 Pfam models) are derived from the combination of sequence predictors : quality-score [20], PSIPred [21, 22], SEG [23] and GlobPlot [24]. Accordingly, the relevant fold-critical and remnant score sums can be derived based on the summations of these annotations. Consequently, the fold-critical and remnant sums are re-evaluated via the model’s EVD statistical model to obtain the corresponding fold-critical E-value and remnant E-value (see Eqs. 1–2 of [19]). Together with the original (undissected) E-values, each sequence-to-domain hit can be classified as a true-positive (TP), false-negative (FN), false-positive (FP) and true-negative (TN) according to predefined criteria in Table 1 (see Methods section “*Classification criteria of sequence*-*to*-*domain alignment hits*” and the table therein). Only TP and FN hits will be retained for subsequent analysis.

**Table 1:** Classification of sequence-to-domain alignment HMMER2 and HMMER3 hits

Version	Classification	Original E-value	Fold-critical E-value	Remnant E-value
HMMER2	TP (True-positive)	≤0.1	≤10^−3.45^	-
	FP (False-positive)	≤0.1	>10^−3.45^	≤10^−3.45^
	TN (True-negative)	>0.1	>10^−3.45^	-
	FN (False-negative)	>0.1	≤10^−3.45^	-
HMMER3	TP (True-positive)	≤10^−3^	≤10^−6^	-
	FP (False-positive)	≤10^−3^	>10^−6^	≤10^−6^
	TN (True-negative)	>10^−3^	>10^−6^	-
	FN (False-negative)	>10^−3^	≤10^−6^	-

The original E-value of hit is set at 0.1 as recommended by the HMMER2 manual [32] which gives a false-positive rate of 0.53. Using the latter as reference, the equivalent original E-value for HMMER3 is set at 10^−3^ (false-positive rate of 0.55). For the fold-critical E-value, the false-positive rate is preset at 0.1. These corresponds to the values of 10^–3.45^ and 10^–6^, respectively. ‘-’ denotes “don’t care” condition

In the final stage, two domain coverage scores, coverage^ratio^ and coverage^fold ‐ critical E ‐ value^ (between value of 0 and 1; corrected by some empirical false-positive rates) are calculated for each pair of overlapping HMMER2/HMMER3 sequence-to-domain alignment (see Eq. 5). In retrospect, the false-positive rates are associated to the fold-critical scores through two surrogate measures of (i) fold-critical E-value and (ii) ratio of fold-critical E-value versus remnant E-value (see Eq. 3) which are underpinned by two pairs of empirical false-positive rate distributions (one for HMMER2, another for HMMER3) that reflect the relationship between the negative hits to a list of 1330 SCOP superfamilies to Pfam domains mappings (see Methods section “*Quantifying the false*-*positive rates of dissected fold*-*critical score associated measures using the SCOP superfamilies to Pfam domains mappings*” and the figures therein).

Taken together, dissectHMMER with its deeper search depth, aims to gather a cohort of statistically confident domain hits that has good fold similarity to the query sequence. Instead of typically limiting oneself to examine only the best domain hit to the sequence, the collective view constructed from a set of fold-related (ideally homologous) domains can minimally postulate the generalized biological function of the query sequence. This is further fuelled by the fact that experimental protein studies occur under different conditions and cell-specific context. Hence, the task of cataloguing the complete biological function of any protein is a time-extended effort [17]. In contrast, dissectHMMER offers an opportunity to build a collective and quick glimpse of the possible dated biological function of a novel protein, albeit in-silico.

### Statistical weighted combination of sequence predictors (quality-score, PSIPred, SEG, GlobPlot) improves the sensitivity and specificity of fold-critical residues detection in domain models

As an integral part of dissectHMMER, the annotation of the Pfam domains which denotes the positions of the fold-critical and remnant residues in the models, allows for the computations of fold-critical and remnant sums of a sequence-to-domain alignment. For Pfam domains with representative structure, the identification of their fold-critical residues can be resolved using the PDB/DSSP information. For those without representative structures, the identification becomes a prediction task. Previously, the quality-score (that measures sequence conservation [20]) has been investigated and was found to have the tendency to underestimate remnant segments.

In this work, the task of predicting the fold-critical residues in domain models through a weighted combination of several sequence property predictors was investigated. These predictors are quality-score (that predicts sequence conservation [20]), PSIPred (that predicts secondary structures like α-helices and β-strands [21, 22]), SEG (that finds low-complexity regions [23]) and GlobPlot (that predicts segments of globularity [24]). The full annotation procedure is described in detail in the Methods section “*Annotation of Pfam domain models into their fold*-*critical and remnant residues*…” and the weighted-scoring scheme equation follows Eqs. 1–2 therein.

To evaluate the performance in terms of sensitivity (i.e. true-positive rate) and specificity (i.e. false-positive rate) for each of the predictors, they were benchmarked against a reference set of 6599 Pfam domains with PDB/DSSP information (see Additional file 1). The respective true-positive rate (TPR) and false-positive rate (FPR) at each threshold level are provided in Additional file 2: Table S1.

We measured the score performance via the difference (TPR-FPR) between the true-positive rate (TPR) and false-positive rate (FPR) at each threshold level. Based on the data from Additional file 2: Table S1, quality-score [20], PSIPred [21, 22], SEG [23] and GlobPlot [24] obtained their best predictive performance at (TPR-FPR) of 0.61, 0.50, 0.41 and 0.39 respectively. The latter serves as the predictor-specific weight variables *w*_*predictor*_ in the proposed weighted-scoring scheme (see Eqs. 1–2) which combines the four predictors’ outputs into a singular value *normscore*_*weighted*_. As such, the sensitivity and specificity of the weighted-scoring scheme can also be computed and is provided in Additional file 2: Table S2.

**Fig. 2:**
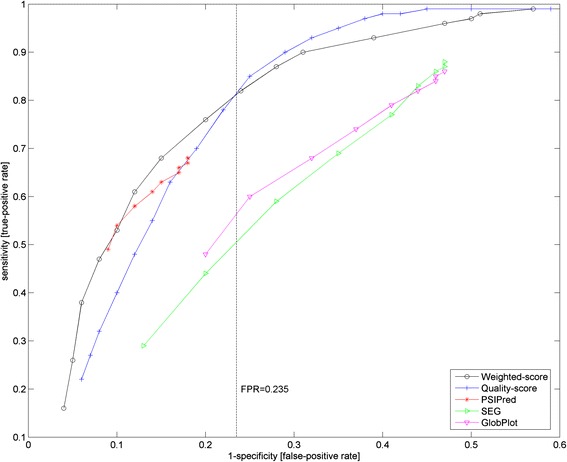
ROC (Receiver operator curve) of weighted-scoring, quality-score, PSIPred, SEG and GlobPlot sequence predictor against 6599 Pfam-to-PDB mappings. For each sequence predictor, 19 data points form each plot (Additional file 2: Tables S1 and S2). However, in the case of PSIPred, SEG and GlobPlot, several of their data points coincide to the same positions for the lower thresholds of between 0.05 and around 0.50. As such, the sensitivity and specificity for PSIPred, SEG and GlobPlot cannot be improved beyond certain thresholds. Quality-score by itself is a better predictor than SEG and GlobPlot but worse than PSIPred for a limited range. Meanwhile, the weighted-scoring scheme performs the best for false-positive rate (i.e. 1-specificity) of less than 0.235 (see *vertical dotted line*, Fig. 3). Beyond that, the quality-score takes over as the better predictor. However, since larger false-positive rate values are typically avoided, the slight inferior performance of the weighted-scoring scheme beyond the false-positive rate of 0.31. Generally speaking, the better performance of the weighted-scoring scheme than any single predictor is due to the statistical weighing step where the contributions of the better predictors are made more significant

Figure 2 shows the ROC (receiver operator curve) plots for the 4 individual predictors (quality-score [20], PSIPred [21, 22], SEG [23] and GlobPlot [24]) and the weighted-scoring scheme by plotting the true-positive rate (i.e. sensitivity) and false-positive rate (i.e. 1-specificity) values from Additional file 2: Tables S1 and S2. Given the threshold range of between 0.05 and 0.95 (at an interval of 0.05), one would expect 19 data points along each plot given. However, in the case of PSIPred [21, 22], SEG [23] and GlobPlot [24], several of their data points coincide to the same positions for lower thresholds of between 0.05 and around 0.50 (see Additional file 2: Table S1). This implies that beyond a certain threshold, the sensitivity and specificity for PSIPred [21, 22], SEG [23] and GlobPlot [24] cannot be improved. Interestingly, quality-score [20] by itself is a better predictor than SEG [23] (consistent with our previous findings [19]) and GlobPlot [24] but worse than PSIPred [21, 22], albeit just for a limited range.

Generally speaking, the weighted-scoring scheme performed the best amongst all the predictors, especially for false-positive rate (i.e. 1-specificity) of less than 0.235 (see vertical dotted line, Fig. 2). Beyond this false-positive rate, the quality-score [20] takes over as the better predictor. But since larger false-positive rate values are typically avoided when choosing practical settings, one should not be overly critical of the slightly lower performance of the weighted-scoring scheme beyond this false-positive rate. In addition, the weight-scoring scheme has its best predictive performance at the (TPR-FPR) of 0.59 (see Additional file 2: Table S2). This corresponds to the false-positive rate of 0.28–0.31. Going for the lower false-positive rate of 0.28, the weighted-scoring scheme then declares a fold-critical residue at a threshold level of ≥0.5. Consequently, the predicted annotations of fold-critical residue in the 14,831 Pfam domains were computed with the weighted-scoring scheme and are provided as Additional file 3.

Taken together, the weighted-scoring scheme has shown to predict fold-critical residues better than any single predictor. Mathematically, this is attributed to the statistical weighing step where the contributions of the better predictors were made more significant. Aside that, the diversity of sequence predictors with different sequence property measure (sequence conservation, secondary structures, globularity, complexity) also allow for a multi-dimensional coverage of fold-critical residues.

### At least 10 % of Pfam library (release 27) contain domain models with fold-critical residues less than that of remnant while at least 14 % of remnant segments are as long as small domain models

As a result of resolving fold-critical residues in Pfam domains in the preceding section, two sets of annotated Pfam domain were derived. A set of 6599 domain models with representative PDB/DSSP entries where locations of the fold-critical or remnant residues with respect to each domain model can be straightforwardly resolved, and another set of the full 14,831 Pfam domains where the fold-critical and remnant residues are predicted using a weighted set of calibrated sequence predictors (quality-score [20], PSIPred [21, 22], SEG, GlobPlot [24]).

**Fig. 3:**
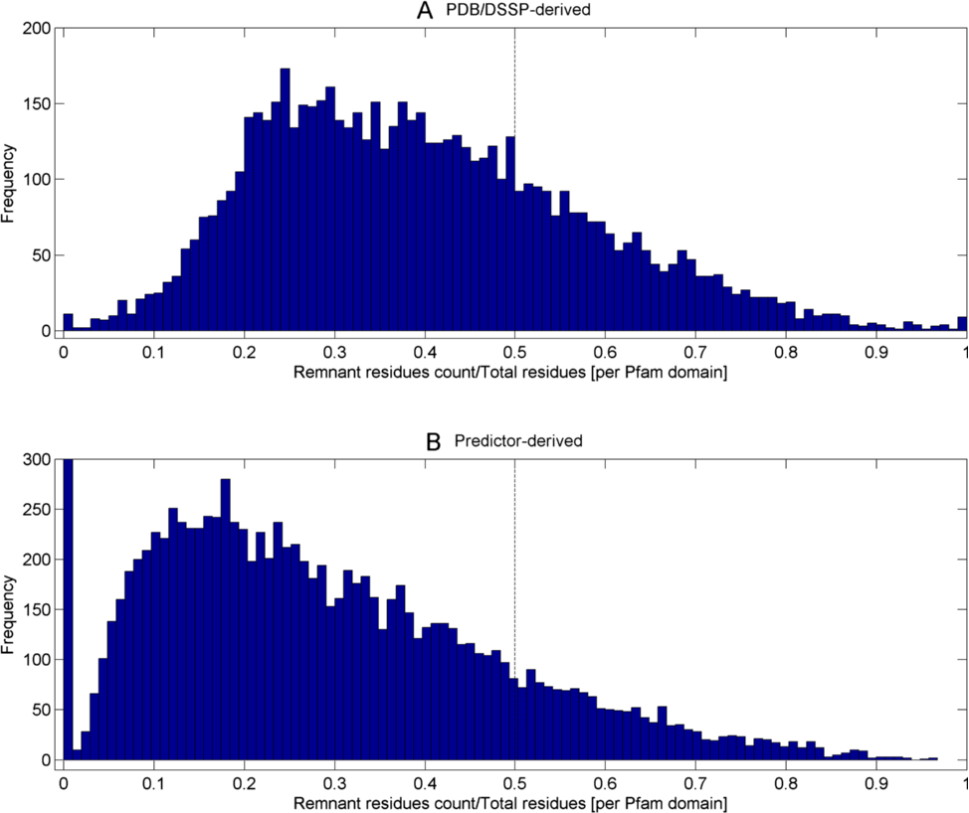
Ratio of remnant residues versus total residues per domain model for the sets of 6599 PDB/DSSP-derived and 14,831 predictor-derived Pfam domain annotations. At a ratio of >0.5, 26.8 % (1767 out of 6599; see *dotted line*) and 10.3 % (1522 out of 14,831; see *dotted line*) of the domains from the PDB/DSSP-derived and predictor-derived sets have more remnant residues than fold-critical residues in each of these models respectively. In addition, 8 PDB/DSSP-derived and 44 predictor-derived domain models have no fold-critical residues (ratio of 1) and they contain either compositionally-biased or disordered sequences

Figure 3 depicts the ratio of remnant residues versus total residues per domain model for the sets of 6599 PDB/DSSP-derived (Fig. 3a) and 14,831 predictor-derived (Fig. 3b) Pfam domain annotations. At a ratio of >0.5, 26.8 % (1767 out of 6599) and 10.0 % (1482 out of 14,831) of the domains from the PDB/DSSP-derived and predictor-derived sets have more remnant residues than fold-critical residues in each of these models, respectively. Given that the derivation is expected to be more accurate in the PDB/DSSP-derived annotations than the predictor-derived ones, 10.0 % serves as a lower estimate of models with the propensity to attract spurious sequence similarities via the more abundant remnant residues. Furthermore, 8 PDB/DSSP-derived domain models have no fold-critical residues (ratio of 1). For these domain models, their model lengths were relatively short and they vary between 13 and 120 (median is 51) AA long. Most importantly, they hit the non-globular region of their representative PDB structures (see Additional file 4 for the sequence-to-domain alignments). As such, such domain models should be excluded when the HMMER search task is aimed at homology inference.

**Fig. 4:**
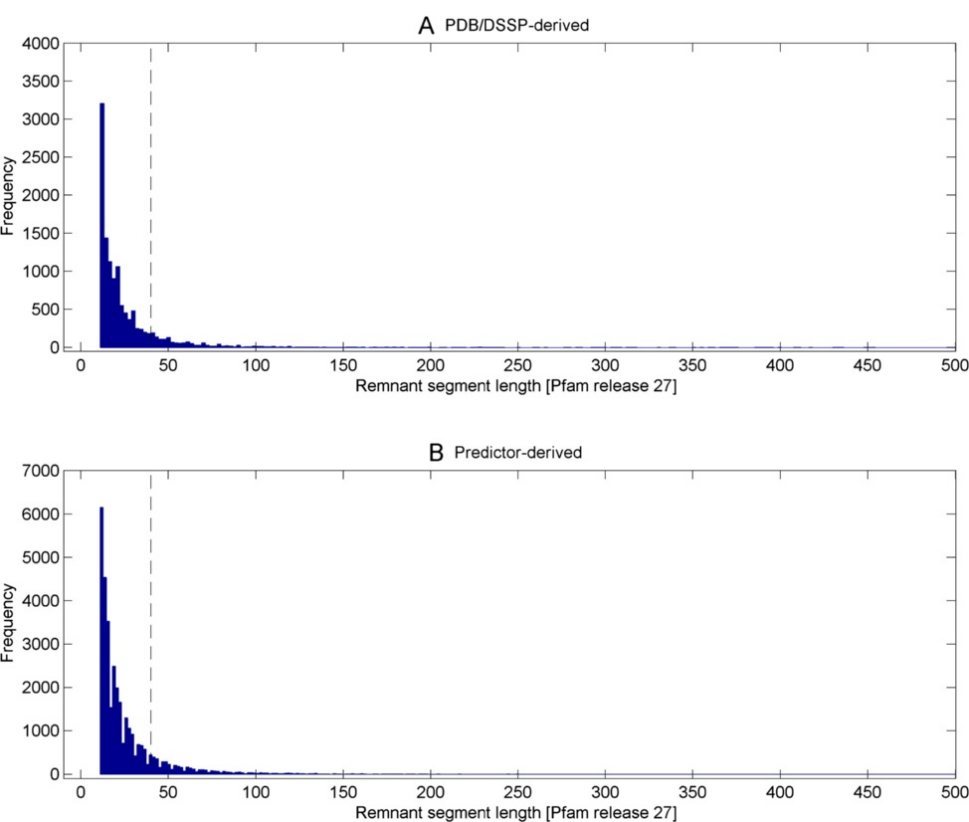
Histograms of remnant segment lengths (i.e. continuous stretches of remnant residues of >10AA) for the PDB/DSSP-derived and predictor-derived Pfam domain sets. A total of 12,253 and 33,774 remnant segments were derived from the 6599 PDB/DSSP-derived and 14,831 predictor-derived domain set respectively. With reference to small domain model of about 40 AA (609 Pfam domains are of lengths 40 AAs or less), the respective domain sets contain 14.6 % (1789 out of 12,253; see *dotted line*) and 15.3 % (5155 out of 33,774; see *dotted line*) of remnant segments with lengths exceeding 40AA

Figure 4 depicts the histograms of remnant segment lengths (i.e. continuous stretches of remnant residues of >10AA) for the PDB/DSSP-derived (Fig. 4a) and predictor-derived (Fig. 4b) Pfam domain sets. A total of 12,253 and 33,788 remnant segments were derived from the 6599 PDB/DSSP-derived and 14,831 predictor-derived domain set respectively. Using a small domain model of about 40 AA like the zinc fingers as reference (609 Pfam domains are of lengths 40 AAs or less based on release 27), the respective domain sets contain 14.6 % (1789 out of 12,253) and 15.2 % (5130 out of 33,788) of remnant segments with lengths exceeding 40AA.

Collectively, the above findings strongly suggest that certain domains have the propensity to attract spurious sequence similarity during homology search. Unfortunately, in practice, other than the obvious removal of signal peptides, simple transmembrane helices and non-globular segments that flank either the beginning or the ending of domain models [3, 27–31], it is unrealistic to create domain models totally without the remnant residues since structural segments are naturally stitched by inter-linkers. Therefore, the latter reinforces the need to dissect any sequence-to-domain alignments into their fold-critical and remnant sums for further statistical re-evaluation prior to making any homology inference; dissectHMMER is a pragmatic step towards homology inference.

### For a given false-positive rate, HMMER3 sequence-to-domain hit’s fold-critical E-value needs to be more stringent than its HMMER2’s counterpart in glocal mode

Previously, an unresolved issue with respect to the score dissection framework was the selection of the fold-critical E-value cutoff for including potential true hits. Based on the HMMER manuals, the recommended E-value cutoff was 0.1 for trusted hits in HMMER2 (see pg 43 of [32]), while it was less well-defined for HMMER3 at an E-value cutoff of << 1 (see pg 19 of [33]). Now, taking the better defined E-value of 0.1, it still remains unclear (i) if the cutoff of 0.1 is appropriate for the fold-critical E-value cutoff for trusted hits inclusion and (ii) whether it is justifiable to use the same fold-critical E-value cutoff for both HMMER2 and HMMER3, given that their algorithmic and parameterization differences.

To properly resolve these issues, we calibrate HMMER2 and HMMER3 E-values against a set of sequence-structure-Pfam domain assignments. While the SCOP superfamilies were derived from alignments of similar structures, Pfam domains were created from alignments of homologous sequences. Essentially, the SCOP-to-Pfam mappings create a set of distant homologous Pfam domains for each SCOP superfamily where its sequences share good fold similarity. Through the sampling of the fold-critical E-value range of the negative domain hits in the vicinity of the SCOP-to-Pfam sequences, it allows dissectHMMER to examine the extent to which the fold-critical score can be used to infer fold similarity (structural alignment space) from sequence similarity (sequence alignment space).

Therefore, a set of 1330 mapped SCOP superfamilies (version 1.75) to Pfam domains (release 27) was created where each SCOP superfamily (with an average of 16.5 sequences) can be mapped to an average of 4.8 Pfam domains (see Additional file 4). For details of the SCOP-to-Pfam mapping creation, readers are referred to Methods section “*Creation of a SCOP superfamilies*…”.

Altogether, there are a total of 22,001 sequences in the 1330 SCOP superfamilies set. Thereafter, each sequence in the SCOP superfamily was searched against 14,831 Pfam domain models to generate a list of negative domain hits (i.e. domain hits that cannot be mapped to the particular SCOP superfamily where the query sequence originates from). The negative domain hits were then dissected into the fold-critical and remnant scores and then evaluated for their corresponding fold-critical and remnant E-values. Altogether, 136,642 HMMER2 and 41,506 HMMER3 fold-critical E-values of the negative domain hits were generated over the 22,001 SCOP sequences. Consequently, using Eq. 3 (see Methods section), the false-positive rates of the fold-critical E-values were computed for the sets of HMMER2 and HMMER3 negative hits at each threshold level over a common threshold range of 10^–205^ to 10^3^ (at an unit step in the logarithm of 10; see Additional file 5). For details, see Methods section “*Quantifying error rates of dissected fold*-*critical scores*”.

**Fig. 5:**
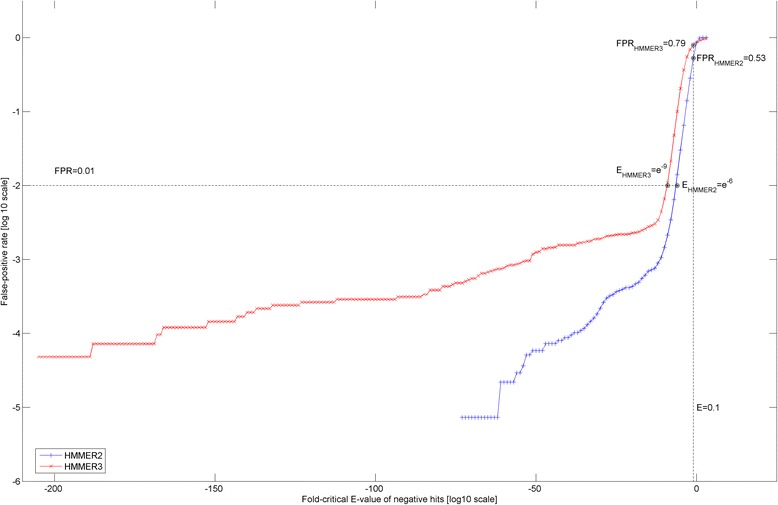
Fold-critical E-values of the negative hits (in logarithm of 10) versus their corresponding false-positive rates (in logarithm of 10). At the recommended E-value cutoff of 0.1 (see *vertical dotted line*), the HMMER2 false-positive rate is lower (at 0.53) than that of HMMER3 (at 0.79). The equivalent HMMER3 E-value cutoff to achieve the same false-positive rate would have been 10^–3^. Conversely, if a false-positive rate of 0.01 (see *horizontal dotted line*) is set, the corresponding HMMER2 and HMMER3 fold-critical E-value cutoffs corresponds to 10^–6^ and 10^–9^ respectively

Figure 5 depicts the fold-critical E-values of the negative hits (in logarithm of 10) versus their corresponding false-positive rates (in logarithm of 10). At the recommended E-value cutoff of 0.1 (see vertical dotted line), the HMMER2 false-positive rate is lower (at 0.53) than that of HMMER3 (at 0.79). The equivalent HMMER3 E-value cutoff to achieve the same false-positive rate would have been 10^–3^. The latter falls within the recommended E-value of << 1 by the manual (see pg 19 of [33]). Conversely, if one goes for a preset false-positive rate of 0.01 (see horizontal dotted line), the corresponding HMMER2 fold-critical E-value cutoff will be at 10^–6^ and a much smaller fold-critical E-value cutoff of 10^–9^ for HMMER3.

Taken together, the original recommended E-value cutoff of 0.1 is probably too generous for the fold-critical E-value cutoff since the false-positive rates are both over 0.50 for the HMMER variants. Conversely, if one opts to control for a common false-positive rate between the HMMER variants, then the fold-critical E-value cutoff of both HMMER algorithms need to be set at different levels, with the case of HMMER3’s being more stringent.

In hindsight, it must be emphasized that this is not a comprehensive comparative study between HMMER2 and HMMER3 given the differences in the alignment modes (glocal versus local; fragmented versus contiguous full alignment). Moreover, it is unreasonable to assume a one-to-one correspondence relationship between HMMER2 and HMMER3 for the case of negative domain hits. Rather, we only wish to highlight that the HMMER3 local alignments do have a general tendency towards more significant E-values than HMMER2 glocal alignments at the same false-positive rate (see Fig. 5). Unfortunately, since HMMER3 does not currently offer glocal mode, readers need to enforce a stricter E-value cutoff for admitting trusted hits when they migrate from the glocal mode of HMMER2 to HMMER3. For dissectHMMER, the preceding finding necessitates for a scoring method that normalizes for the differences in false-positive rates generated by HMMER2 and HMMER3 when unifying overlapping (i.e. common sequence coverage for the same domain model) sequence-to-domain hits between HMMER2 and HMMER3.

### Segregation between fold-critical and remnant sequences, dissectHMMER improves the sensitivity and specificity of domain detection with similar fold over profile-to-profile method-HHsearch

As discussed earlier, the respective false-positive rates associated to any overlapping HMMER2 and HMMER3 sequence-to-domain alignments can vary greatly at the same E-value cutoff. Although in mitigation, one may preset independent HMMER2-specific and HMMER3-specific fold-critical E-value cutoffs to limit hits beyond a common false-positive rate, this will inevitably cause some of the paired or overlapping HMMER2 and HMMER3 alignments to be orphaned. As a consequence, this may create a bias towards one of the HMMER alignments; for better or worse. Also, besides achieving statistical significance, the sequence-to-domain hit should also reflect some level of fold similarity of the domain model. This issue is particularly relevant to the HMMER3 hits since HMMER3 returns fragmented alignments, thus satisfying only partial domain fold similarity. In the case of HMMER2 hits, domain coverage is always one, since the glocal mode can be enforced.

With the preceding background considerations, an error-adjusted domain coverage measure is proposed as shown by Eq. 5 (see Methods section “*Error*-*adjusted domain coverage score*: *combining HMMER2 and HMMER3 fold*-*critical measures and domain coverage*” and equations therein). This measure gives a singular value for each pair of HMMER2/HMMER3 sequence-to-domain alignments that is computed from both HMMER2-specific and HMMER3-specific false-positive rates and domain coverages.

To backtrack, dissectHMMER creates a pair of fold-critical and remnant score for each sequence-to-domain alignment that is subsequently evaluated for its fold-critical E-value and its remnant E-values. As such, two surrogate measures of the fold-critical score can be derived : (i) fold-critical E-value and (ii) ratio of fold-critical E-value over remnant E-value.

To elaborate further, the fold-critical E-value indicates the level of statistical significance of the fold-critical components in the alignment while ratio reflects the magnitude difference between the fold-critical and the remnant parts of the alignment (low ratio suggests the fold-critical components dominates the statistical significance of the alignment). A confident sequence-to-domain hit is characterized by low fold-critical E-value and low ratio. In any case, both fold-critical E-value and ratio can be associated to some level of false-positive rate as part of the error-adjusted domain coverage formula. Therefore, two versions of the error-adjusted domain coverage for the paired HMMER2/HMMER3 alignments can be evaluated for, as represented by coverage^ratio^ and coverage^fold ‐ critical E ‐ value^ (see Eq. 5).

For the purpose of evaluating the error-adjusted domain coverage measure of dissectHMMER, it was pit against the well-regarded profile-to-profile HMM method–HHsuite/HHsearch. The two algorithms were evaluated across 1330 SCOP superfamilies where a multiple sequence alignment of each SCOP superfamily profile was generated using the clustalw program and presented to the respective algorithms as inputs. To reiterate, each SCOP superfamily fold can be shared by several Pfam domain models. Meanwhile, since dissectHMMER is fundamentally a sequence-to-profile method, some additional steps were added to allow dissectHMMER to derive a domain-wise score measure (see Eq. 6) comparable to that of HHsearch for this evaluation.

As usual, each sequence of the SCOP profile was first processed by the dissectHMMER workflow (see section “*The HMMER score dissection framework*: *dissectHMMER webserver*”) to generate a list of sequence-to-domain hits and their associated error-adjusted domain coverage score measures (i.e. coverage^ratio^ and coverage^fold ‐ critical E ‐ value^). Then, the collection of all sequence-to-domain hits for this SCOP profile were sorted into groups of individual domain models. As such, the averages of error-adjusted domain coverage scores per domain model can be computed (see Eq. 6) in “*SCOP superfamily*-*wise evaluation of dissectHMMER*” section of Methods). Essentially, the average is the domain-wise score measure (domainscore_*k*_^ratio^ or domainscore_*k*_^fold − critical E ‐ value^ for the k^th^ domain model) for the dissectHMMER algorithm.

Meanwhile, for the HHsuite/HHsearch algorithm (taken from ftp://toolkit.genzentrum.lmu.de/pub/HH-suite/), the SCOP profile was directly used to sea rch against the HHsearch-specific Pfam release 27 database (PfamA_27.0.hhm downloaded from ftp://toolkit.genzentrum.lmu.de/pub/HH-suite/databases/hhsearch_dbs/). In addition, the search model was set to the “local alignment” mode to maximize for search sensitivity. For each SCOP superfamily profile, the HHSearch algorithm presented a list of domain-to-domain hits that were sorted based on their probabilities, E-values (database size of 14,831 based on Pfam release 27) and P-values. For this comparison, the probability score of each domain-to-domain from HHsearch is taken as its best measure against that of dissectHMMER’s.

In total, the dissectHMMER algorithm computed the domain-wise score measures for all 1330 SCOP superfamilies (with 6339 mappable Pfam domains) while only 1199 SCOP superfamilies for the HHsearch algorithm. In hindsight, some SCOP profiles contain information entropy that was too low for the HHsearch to create its native HMM representation. In any case, to ensure that comparison is fair, only the common 1199 superfamilies (with 5966 mappable Pfam domains) between the two algorithms were further analyzed. This made up 80,592 domainscore^ratio^ and 80,592 domainscore^fold − critical E ‐ value^ for dissectHMMER and 39,695 HHsearch probability scores. Consequently, the sets of domain-wise scores for both dissectHMMER and HHsearch were evaluated for the algorithms’ specificity and sensitivity over the score range of 0 to 1 at an interval of 0.01.

As a baseline prior to applying score dissection, the HMMER3 algorithm was also evaluated at three E-value cutoffs of 0.1, 1 and 10. For each of the cutoffs, only the sequence-to-domain alignment hits below the designated E-value thresholds (i.e. trusted hits; assuming false-positive rate of 0) were kept and the domain-wise score measure is simply the domain coverage. This implies that the terms (1 − *fpr*_measure_^HMMER2^)coverage^HMMER2^ and *fpr*_measure_^HMMER3^ in Eq. 5 are both set to zero. Consequently, this made up 12,871 (at E-value ≤0.1), 18,191 (at E-value ≤1) and 20,672 at (E-value ≤10) HMMER3 domain coverage values.

**Fig. 6:**
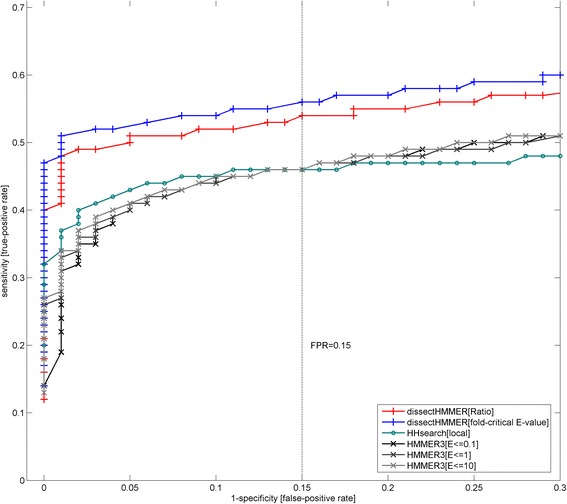
ROC (receiver operator curve) plots for dissectHMMER, HHsearch and HMMER3 against SCOP-to-Pfam mapping set. The domain detection for the SCOP superfamily (based on fold similarity) is generally a difficult task for sequence-based search methods. Towards the false-positive rate of 1, HHsearch, HMMER3 and dissectHMMER only detected up to 50 % (3067), 63 % (3767) and 65 % (3890 for fold-critical measure; 3894 for ratio measure) of the 5966 mappable Pfam models respectively. At the false-positive rate (i.e. 1-specificity) of below 0.15, HMMER3 (a sequence-to-profile method) performed worse than HHsearch (a profile-to-profile method) as expected. Beyond 0.15, HMMER3 picked up higher sensitivities than HHsearch when false-positive rates went over 0.15 but large false-positive rate thresholds are rarely considered. Meanwhile, dissectHMMER’s plots hovered above that of both HMMER3 and HHsearch by a considerably margin, thus suggesting that it is more capable at detecting Pfam domains that share a superfamily fold; thus, better bridging sequence similarity search space and structural similarity search space. Note that the error rates are separately derived from the empirical distributions of the negative domain hits in the vicinity of the SCOP-to-Pfam sequences

Figure 6 depicts the ROC plots for dissectHMMER, HHsearch and HMMER3. Generally speaking, the domain detection for the SCOP superfamily (based on fold similarity) proves to be a formidable task for all the sequence-based search methods. Towards the extreme false-positive rate of about 1, the HHsearch, HMMER3 and dissectHMMER algorithms only detected up to 50 % (3067), 63 % (3767 for E-value ≤0.1; 3779 for E-value ≤1; 3782 for E-value ≤10) and 65 % (3890 for fold-critical measure; 3894 for ratio measure) of the 5966 possible Pfam models respectively.

At the false-positive rate (i.e. 1-specificity) of below 0.15, HMMER3 which belongs to the sequence-to-profile method, performed worse than the profile-to-profile method HHsearch as expected. And although HMMER3 picked up higher sensitivities than HHsearch when false-positive rates went over 0.15, large false-positive rate thresholds are seldom considered since they admit a large number of false hits.

Most importantly, when the score dissection framework is integrated into the HMMER algorithm, the end results are a set of dissectHMMER’s ROC plots that hover above that of both HMMER3 and HHsearch by a considerably margin (see Fig. 6). At a false-positive rate of 0.15, the increase in true-positive rate (or the recovery of false-negatives) by dissectHMMER was between 8 and 10 % over that of HMMER3 with a true-positive rate of 46 % (54 % for the ratio measure; 56 % for the fold-critical E-value measure). This increase of 8–10 % is in the same order of magnitude as the average false-negative rate of 4.86 (± 10.27)% generated by the Pfam domains when searched against the SwissProt/UniProt database from our previous work [19].

Taken together, dissectHMMER is more capable at detecting Pfam domains that share a superfamily fold; thus, better bridging sequence similarity search space and structural similarity search space. This better performance is underpinned by the error-adjusted domain coverage score measures coverage^ratio^ and coverage^fold ‐ critical E ‐ value^ in dissectHMMER that critically corrects the fold-critical sums of the sequence-to-domain hits with estimated false-positive rates.

In hindsight, the score dissection concept can benefit the supposedly less sensitive sequence-to-profile search method by outperforming the more superior profile-to-profile search method (e.g. HHsearch) as exemplified by dissectHMMER. It would hardly be surprising if the “dissected” version of HHsearch would improve upon itself and over dissectHMMER as well, though it is beyond the scope of this work. Overall, the improvement brought about by score dissection asserts the necessity of segregating between the fold-critical and remnant residues that is deeply rooted in the core of the homology inference problem. And beyond mere numerical improvements, dissectHMMER’s implementation is the most faithful to the sequence homology concept as compared to other current search algorithms since only the structural residues (i.e. fold-critical segments) will be considered for inferring homology.

### Case studies of dissectHMMER improves the confidence of protein function prediction/annotation

**Fig. 7:**
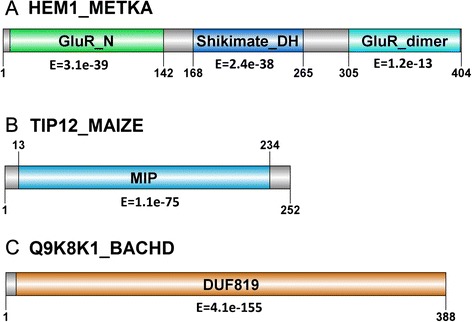
Domain architecture of sequence examples HEM1_METKA, TIP12_MAIZE and Q9K8K1_BACHD. The sequences were analyzed by the Pfam webserver (http://pfam.xfam.org/). Briefly, HEM1_METKA has three domains: GlutR_N (PF05201.10) with E-value of 3.1e–39 from positions 7–142, Shikimate_DH (PF01488.15) with E-value of 2.4e–38 from positions 156–292 and GlutR_dimer (PF00745.15) with E-value of 1.2e–13 from positions 305–403 (See Fig. 7a). In the case of TIP12_MAIZE, it has a single domain hit to MIP (PF00230.15) with E-value of 1.1e–75 from positions 13–234 (See Fig. 7b). Lastly, the sequence Q9K8K1_BACHD has a single domain hit to an unknown domain DUF819 (PF05684) with E-value of 4.1e–155 from positions 10–388 (See Fig. 7c). Generally speaking, there is no way to assert if the significance of these domain hits are mainly attributed to the fold-critical sequence segments of the sequence-to-domain alignments. In the case of Q9K8K1_BACHD, the hit to an unknown domain offers little information pertaining to its plausible biological function

In this section, the improved postulation of biological function for protein sequences by dissectHMMER, beyond that of the typical Pfam/HMMER analysis, was demonstrated through the analysis of three UniProt sequences: HEM1_METKA (NP_613487.1), TIP12_MAIZE (NP_001105029.1) and Q9K8K1_BACHD (WP_010899149.1; completely uncharacterized). These sequences were first analyzed by the Pfam webserver (http://pfam.xfam.org/) and their domain architectures are depicted in Fig. 7. Briefly, HEM1_METKA has three domains: GlutR_N (PF05201.10) with E-value of 3.1e–39 from positions 7–142, Shikimate_DH (PF01488.15) with E-value of 2.4e–38 from positions 156–292 and GlutR_dimer (PF00745.15) with E-value of 1.2e–13 from positions 305–403 (See Fig. 7a). In the case of TIP12_MAIZE, it has a single domain hit to MIP (PF00230.15) with E-value of 1.1e–75 from positions 13–234 (See Fig. 7b). Lastly, the sequence Q9K8K1_BACHD has a single domain hit to an domain of unknown function DUF819 (PF05684) with E-value of 4.1e–155 from positions 10–388 (See Fig. 7c). Generally speaking, there is no way to assert if the significance of these domain hits are mainly attributed to the fold-critical sequence segments of the sequence-to-domain alignments to justify for function annotation transfer. Implicitly, this requires some leap of faith based on a single significant domain hit. And in the case of Q9K8K1_BACHD, faith on a significant yet unknown domain hit offers little clue as to the plausible biological function of the sequence.

**Fig. 8:**
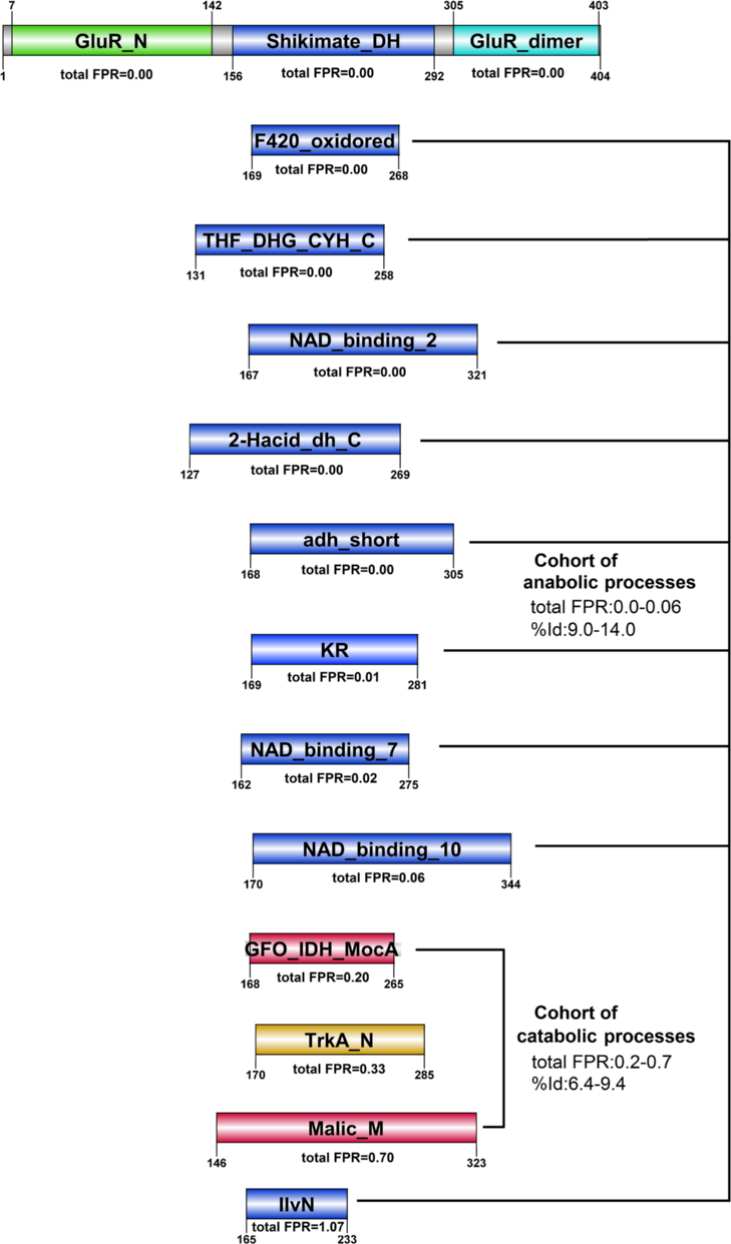
DissectHMMER analysis of HEM1_METKA. The domain hits can mainly be generalized as cellular metabolism reactions and can be further subdivided into 2 main groups : (i) 10 anabolic reactions that combine simple substances into more complex molecules driven by NADP+ and (ii) 2 catabolic reactions that breaks complex organic molecules into simpler substances driven by NAD+. With the exception of the IlvN domain (PF07991.7), the top 9 domain hits Shikimate_DH (PF01488.15), F420_oxidored (PF03807.12), THF_DHG_CYH_C (PF02882.14), NAD_binding_2 (PF03446.10), 2-Hacid_dh_C(PF02826.14), adh_short (PF00106.20), KR (PF08659.5), NAD_binding_7 (PF13241.1) and NAD_binding_10 (PF13460.1) are NADP+ driven anabolic processes with a total FPR range of between 0.0 and 0.06 and a structural-derived %Id range of between 9.0 and 14. In the case of the IlvN domain, its sequence-to-domain hit covers only 42 % (75 out of 177 AA) of its NADP+ domain and found by HMMER3 alone. As a result, the total FPR is regretfully high at 1.07 as the HMMER algorithm reaches its limit of detection. The next group of domain hits i.e. GFO_IDH_MocA (PF01408.17) and Malic_M (PF03949.10) binds the NAD+ molecule. This group exhibits a larger total FPR range of between 0.20 and 0.70 and a lower structural-derived %Id of between 6.4 and 9.4. Notably, there was a noticeable jump between the adjacent hits from 0.06 (NAD_binding_10) to 0.20 (GFO_IDH_MocA) when the ligand changes from NADP+ to NAD+ between the anabolic and catabolic groups

Meanwhile, in analysis of HEM1_METKA (sequence length of 404 AA) by dissectHMMER (see Additional file 6), the sequence also exhibited a N-terminus domain hit to the PF05201.10 GlutR_N domain (positions 7–142) and a C-terminus domain hit to PF00745.15 GlutR_dimer domain (positions 305–403). The key difference in results between the Pfam server and dissectHMMER was marked by the middle sequence stretch from positions 168–265 where dissectHMMER generated 13 domain hits while the Pfam webserver generates only a single domain hit (See Fig. 7 versus Fig. 8a). Between the Pfam webserver and dissectHMMER, only the Shikimate_DH (PF01488.15) domain was a common hit. The results are presented in Table 2.

**Table 2:** DissectHMMER results for the analysis of HEM1_METKA for the middle sequence stretch from positions 168–265

Domain description	Sequence range/Domain coverage	Original E-values [HMMER2/HMMER3]	[coverage/FPR]^ratio^[coverage/FPR]^fc E-value^	Total FPR	RMSD/%Id/Structural alignment range (1GPJ|A:pdb)	Function description of representative pdb
PF01488.15	156–292/	1.90e–67/	0.995/0.00	0.00	3.12/	Synthesis of aromatic amino-acids in shikimate pathway [39]; NADP+ driven
Shikimate_DH	1	3.21e–40	0.995/0.00		14.0/	
length:170					149–304:	
pdb:1NVT|A					110–270	
PF03807.12	169–268/	8.73e–06/	0.975/0.00	0.00	3.75/	Conversion of insoluble ferrin (Fe^3+^) to soluble ferrin(Fe^2+^) [40]; NADP+ driven
F420_oxidored	1	2.93e–07	0.980/0.00		14.0/	
length:123					168–281:	
pdb:2VNS|A					29–150	
PF02882.14	131–258/	5.34e–01/	0.780/0.00	0.00	3.29/	Interconversion of 1-carbon derivatives of tetrahydrofolate; substrates for methione, thymidylate and purine syntheses [41]; NADP+ driven
THF_DHG_CYH_C	1	8.87e–03	0.795/0.00		10.7/	
length:205					149–307:	
pdb:1A4I|A					147–296	
PF03446.10	167–321/1	3.00e–01/	0.700/0.00	0.00	3.57/	Decarboxylating reduction of 6-phosphogluconate to ribose 5-phosphate [42]; NADP+ driven
NAD_binding_2		1.27e–03	0.710/0.00		11.4/	
length:235					167–288:	
pdb:1PGQ|A					2–129	
PF02826.14	127–269/	8.79e–03/	0.645/0.00	0.00	3.05/	Purine biosynthesis [43]; NADP+ driven
2-Hacid_dh_C	1	5.29e–09	0.670/0.00		11.8/	
length:260					161–264:	
pdb:3ORQ|A					3–101	
PF00106.20	168–305/	1.10e–02/	0.640/0.00	0.00	3.00/	Synthesis of tripinone from pseudotropine [44]; NADP+ driven
adh_short	1	5.53e–05	0.645/0.00		9.2/	
length:225					164–265:	
pdb:1IPE|A					6–145	
PF08659.5	169–281/1	4.86e + 00/	0.57/0.01	0.01	3.62/	Mammalian fatty acid synthase; a large multienzyme that catalyzes all steps of fatty acid synthesis [45]; NADP+ driven
KR		6.51e–03	0.725/0.00		9.0/	
length:257					150–291:	
pdb:2VZ9|A					1651–1802	
PF13241.1	162–275/	9.13e–02/	0.535/0.01	0.02	4.17/	Siroheme synthesis from uro’gen III in tetrapyrrole biosynthesis [46]; NADP+ driven
NAD_binding_7	1	7.38e–03	0.535/0.01		12.0/	
length:379					160–304:	
pdb:1PJQ|A					5–150	
PF13460.1	170–344/	2.40e–02/-	0.495/0.03	0.06	3.26/	Synthesis of bilverdin from bilirubin [47]; NADP+ driven
NAD_binding_10	1		0.490/0.03		11.5/	
Length:362					168–288:	
pdb:1HDO|A					4–152	
PF01408.17	168–265/1	8.97e–02/-	0.445/0.11	0.20	3.73/	Cleavage of non-reducing N-acetylgactosamine from blood group ABO antigens [48]; NAD+ driven
GFO_IDH_MocA			0.450/0.10		9.4/	
length:188					167–303:	
pdb:2IXB|A					20–164	
PF02254.13	170–285/	3.55e–02/-	0.400/0.18	0.33	3.31/	NAD-mediated conformation switch for K^+^ influx control [49]; NAD+ driven
TrkA_N	1		0.420/0.15		10.5/	
length:195					168–300:	
pdb:1LSS|A					1–132	
PF03949.10	146–323/	3.44e–02/-	0.140/0.66	0.70	3.22/	Oxidation of malate to pyruvate [50]; NAD+ driven
Malic_M	1		0.485/0.04		6.4/	
length:324					149–305:	
pdb:1DO8|A					298–494	
PF07991.7	165–233/	-/1.65e–04	0.205/0.53	1.07	3.49/	Synthesis of branched side of valine and isoleucine [51]; NADP+ driven
IlvN	0.42		0.205/0.54		9.7/	
length:177					163–288:	
pdb:1YVE|A					121–252	

For each domain hit, the Pfam accession, domain name, domain length and representative PDB (if any) are given in column 1. Column 2 gives the sequence range (i.e. sequence stretch covered by the domain) and the domain coverage where 1 indicates full coverage while <1 implies partial coverage by the domain model. Column 3 gives the original (or undissected) HMMER2 and HMMER3 E-values of the sequence-to-domain alignments. Column 4 gives the coverage score, coverage^ratio^ and coverage^fold ‐ critical E ‐ value^ (see Eq. 5) which is the corrected domain coverage score of the HMMER2/HMMER3 sequence-to-domain hit. The expected FPRs (false-positive rates) for the coverage scores are also provided and they were estimated from the relevant dissectHMMER ROC plots in Fig. 6. Column 5 gives the sorted total FPR in ascending order, where the latter is the sum of the two independent FPRs as given in column 3. Column 6 gives the RMSD/%Id and alignment range derived from the structure alignments between 1GPJ|A and the representative structures of the domain models. The last column gives the biological function of the representative structures.

In Table 2, the Pfam accession, domain name, domain length and representative PDB (if any) for each domain hit are given in column 1. Column 2 gives the sequence range (i.e. sequence stretch covered by the domain) and the domain coverage, where 1 indicates full coverage while <1 implies partial coverage by the domain model. Column 3 gives the individual original HMMER2 and HMMER3 E-values for each of the sequence-to-domain alignment. Column 4 gives the corrected domain coverage scores, coverage^ratio^ and coverage^fold ‐ critical E ‐ value^ (see Eq. 5) of the HMMER2/HMMER3 sequence-to-domain hits. The expected FPRs (false-positive rates) for the domain coverage scores are also provided and they were estimated from the relevant dissectHMMER ROC plots in Fig. 6 when given a coverage score. Finally, the list of domains were sorted by the total FPR in ascending order (column 5) where the latter is the sum of the two independent FPRs as given in column 3.

To validate the domain hits as proposed by dissectHMMER for the sequence stretch of 168–265, the PDB structure of HEM1_METKA, 1GPJ|A (sequence length of 404AA), was used to perform structural alignments (via the jCE algorithm [34]) against each of the representative PDB structure of the 13 domains. Briefly, the structure 1GPJ|A is a glutamyl-tRNA reductase involved in the tetrapyrrole biosynthesis of plants and prokaryotes [35]. This reductase contains 3 domains : a N-terminus RNA-binding domain, a NADPH-binding domain (which positional range coincides with the 13 domains proposed by dissectHMMER in Table 2) and a C-terminus dimerization domain. The resulting RMSDs and sequence identities (%Ids) from the structural alignments are tabulated in column 6. Column 6 also includes the aligned range between the structure 1GPJ|A and the respective representative structures (in column 1). Finally, the relevant biological function of these representative structures based on literature review are listed in the last column.

Based on the listed biological functions in Table 2 (last column), the domain hits can mainly be generalized as cellular metabolism reactions and can be further subdivided into 2 main groups : (i) 10 anabolic reactions that combine simple substances into more complex molecules driven by NADP+ and (ii) 2 catabolic reactions that breaks complex organic molecules into simpler substances driven by NAD+. The exception in the list is the TrkA_N (PF02254.13) domain that uses NAD+ to drive conformational change in K+ channels/transporters for osmoregulation [36]. The 13 NADP+ and NAD+ binding domain hits are depicted in Fig. 8.

With the exception of the IlvN domain (PF07991.7), the top 9 domain hits Shikimate_DH (PF01488.15), F420_oxidored (PF03807.12), THF_DHG_CYH_C (PF02882.14), NAD_binding_2 (PF03446.10), 2-Hacid_dh_C(PF02826.14), adh_short (PF00106.20), KR (PF08659.5), NAD_binding_7 (PF13241.1) and NAD_binding_10 (PF13460.1) are NADP+ driven anabolic processes with a total FPR range of between 0.0 and 0.06 and a structural-derived %Id range of between 9.0 and 14. Essentially, this cohort of NADP+ driven biosynthesis processes corroborates well with the notion that the HEM1_HETKA possesses a NADP+ domain to drive its tetrapyrrole biosynthesis. In the case of the IlvN domain, the structural alignment covers about 120 positions between the NADP+ binding domains of 1GPJ|A and 1YVE|A (see last row, column 6; Table 2) supporting the notion that it has a NADP+ binding domain, However, its sequence-to-domain hit covers only 42 % (75 out of 177 AA) of its NADP+ domain and found by HMMER3 alone. As a result, the total FPR is regretfully high at 1.07 as the HMMER algorithm reaches its limit of detection. The next group of domain hits i.e. GFO_IDH_MocA (PF01408.17) and Malic_M (PF03949.10) binds the NAD+ molecule. This group exhibits a larger total FPR range of between 0.20 and 0.70 and a lower structural-derived %Id of between 6.4 and 9.4. Interestingly, there was an noticeable jump between the adjacent hits from 0.06 (NAD_binding_10) to 0.20 (GFO_IDH_MocA) when the ligand changes from NADP+ to NAD+ between the anabolic and catabolic groups. If the original E-values had been used, the distinction between the NADP+ (HMMER2 E-values between 1.9e–67 and 4.86) and NAD+ (HMMER2 E-values between 3.44e–2 and 8.97e–2) domains would be challenging due to the overlapping ranges. Meanwhile, the HMMER3 results would have posed some additional difficulties due to missing E-values for some of these domains.

In hindsight, though the chemical composition between NADP+ and NAD+ only differs by a phosphate, the biological processes that each molecule outlines suggests quite the opposite i.e. building versus breaking down (see section 14.3 of [37]). On this occasion, the deeper search depth to gather more fold-related domains by dissectHMMER and the subsequent hits stratification via the total FPR measure and biological evidence to partition the hits into a cohort of NADP+ domains as a closer and majority group while the NAD+ domains as the distant and minority group, helps to clarify that the middle segment of HEM1_METKA is a NADP+ and not a NAD+ binding domain.

**Fig. 9:**
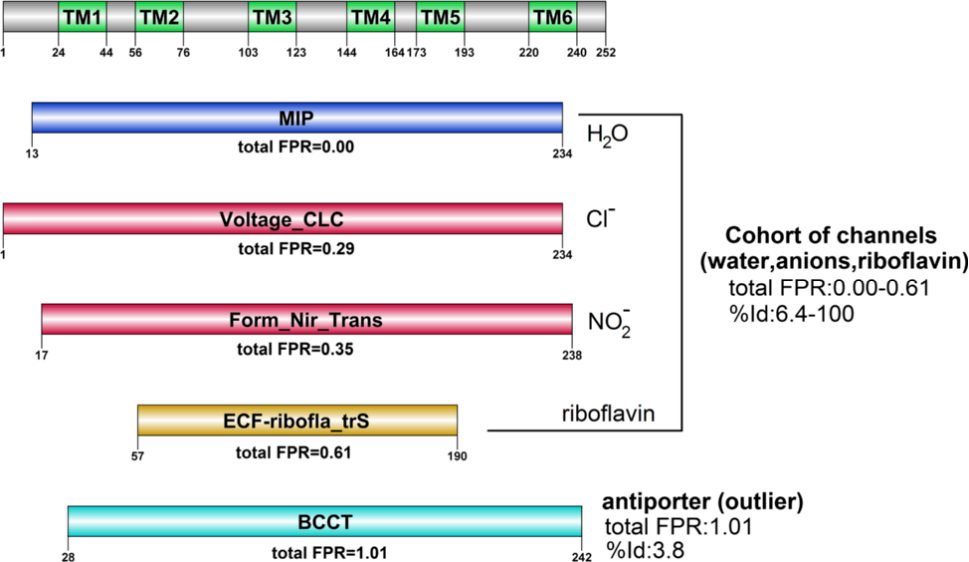
DissectHMMER analysis of TIP12_MAIZE. The most significant domain hit MIP (PF00230) describes a water/glycerol channel that scores a total FPR of 0.0. Interestingly, the next two domain hits Voltage_CLC (PF00654.15) and Form_Nir_tranp (PF01226.12), marks a jump from the MIP domain hit with a total FPR of 0.29 and 0.35 respectively. Their structurally-derived %Ids are 6.6 and 9.5 respectively. This is concurrently accompanied by a change of the transported solute from water (H_2_O) to some anions, chloride (Cl^−^) and nitrite (NO_2_^−^) respectively. The fourth domain ECF-ribofla_trS (PF07155.7) scores a total FPR of 0.61 and structurally-derived %Id of 6.4, and is a 5-TM channel protein that transports riboflavin molecules (generally neutral in charge) across the membrane. Overall, the cohort of domain hits MIP, Voltage_CLC, Form_Nir_tranp and ECF-ribofla_trS generally describes a channel protein that transports its solute across the lipid membrane. In contrast, the last domain hit BCCT (PF02028.12) describes a 12-TM antiporter (concurrent exchange of carnitine and butyrobetaine) that is mechanistically different from a channel protein. This BCCT hit scores an unimpressive total FPR of 1.01. It is further ousted as a positive domain hit by its structural alignment results against TIP12_MAIZE with a bad RMSD of 9.73 and a low %Id of 3.8

In the second example, the analysis of 6-TM (transmembrane) TIP12_MAIZE (sequence length of 252 AA) by dissectHMMER reveals 11 relevant domain hits altogether (see Additional file 2: Table S3 and Additional file 7 for the full list). Out of which, only 5 domain hits have associated structures as listed in Table 3, as sorted by the total FPR measure in ascending order. Furthermore, the 5 domain hits can be sorted into a cohort of 4 channel proteins and a single outlier antiporter protein as depicted in Fig. 9.

**Table 3:** Filtered dissectHMMER results from the analysis of the 6-TM TIP12_MAIZE

Domain description	Sequence range/Domain coverage	Original E-values [HMMER2/HMMER3]	[coverage/FPR]^ratio^ [coverage/FPR]^fc E-value^	Total FPR	RMSD/%Id/Structural alignment range (1YMG|A:pdb)	Function description of representative pdb
PF00230.15	13–234/	1.46e–127/	1.00/0.00	0.00	0.00/	6-TM water/glycerol channel of malarial parasite Plasmodium falciparum [52]
MIP	1	8.50e–73	1.00/0.00		100/	
length:296					6–239:	
pdb:1YMG|A					6–239	
PF00654.15	1–236/	3.89e–01/	0.345/0.28	0.29	4.99/	12-TM chloride channel; 3 Cl^−^ bind sites, each a “pore-like” trajectory transverse to the membrane plane [53]
Voltage_CLC	1	6.52e–05	0.550/0.01		6.6/	
length:730					78–228:	
pdb:2HLF|A					254–383	
PF01226.12	17–238/	3.54e–03/-	0.325/0.32	0.35	3.51/	6-TM nitrite anion channel of bacteria for cytoplasmic detoxification [54]
Form_Nir_trans	1		0.490/0.03		9.5/	
length:366					10–223:	
pdb:4FC4|A					25–249	
PF07155.7	57–190/	8.10e–02/-	0.235/0.48	0.61	3.52/	5-TM pore that transport riboflavin molecules across the lipid bilayer [55]
ECF-ribofla_trS	1		0.435/0.13		6.4/	
ength:196					131–226:	
pdb:4HZU|S					35:162	
PF02028.12	28–242/	7.24e–02/-	0.010/0.98	1.01	9.73/	12-TM carnitine/butyrobetaine antiporter [56]
BCCT	1		0.495/0.03		3.8/	
length:722					6-198:	
pdb:2WSW|A					86–373	

For each domain hit, the Pfam accession, domain name, domain length and representative PDB (if any) are given in column 1. Column 2 gives the sequence range (i.e. sequence stretch covered by the domain) and the domain coverage where 1 indicates full coverage while <1 implies partial coverage by the domain model. Column 3 gives the original (or undissected) HMMER2 and HMMER3 E-values of the sequence-to-domain alignments. Column 4 gives the coverage score, coverage^ratio^ and coverage^fold ‐ critical E ‐ value^ (see Eq. 5) which is the corrected domain coverage score of the HMMER2/HMMER3 sequence-to-domain hit. The expected FPRs (false-positive rates) for the coverage scores are also provided and they were estimated from the relevant dissectHMMER ROC plots in Fig. 6. Column 5 gives the sorted total FPR in ascending order, where the latter is the sum of the two independent FPRs as given in column 3. Column 6 gives the RMSD/%Id and alignment range derived from the structure alignments between 1YMG|A and the representative structures of the domain models. The last column gives the biological function of the representative structures.

With a total FPR of 0.0, TIP12_MAIZE is strongly postulated to be a water/glycerol channel based on the most significant MIP (PF00230.15) domain hit. In turn, the MIP domain has a representative PDB structure 1YMG|A that describes a bovine aquaporin [38]. Then, to validate the relevance of the other 4 domain hits, structural alignments (via the jCE algorithm [34]) were executed for each representative PDB structure of the domains against the PDB structure 1YMG|A. The resulting RMSDs, sequence identities (%Ids), the aligned ranges and biological functions from the structural alignments are tabulated in columns 6 and 7 of Table 3. Interestingly, the subsequent two channel protein domain hits Voltage_CLC (PF00654.15) and Form_Nir_tranp (PF01226.12), marks a jump from the MIP domain hit with a total FPR of 0.29 and 0.35 respectively. Their structurally-derived %Ids are 6.6 and 9.5 respectively. This is concurrently accompanied by a change of the transported solute from water (H_2_O) to some anions, chloride (Cl^−^) and nitrite (NO_2_^−^) respectively. The fourth domain ECF-ribofla_trS (PF07155.7) scores a total FPR of 0.61 with a structurally-derived %Id of 6.4, and is a 5-TM channel protein that transports riboflavin molecules (generally neutral in charge) across the membrane. Despite the differences in the substrate specificity, the cohort of domain hits i.e., MIP, Voltage_CLC, Form_Nir_tranp and ECF-ribofla_trS generally describes a channel protein that transports its solute across the lipid membrane. This cohort ranges a total FPR of between 0.00 and 0.61 and a structurally-derived %Id of between 6.4 and 100. Although the Pfam server was also able to conclude that TIP12_MAIZE is an aquaporin (see Fig. 7b versus Fig. 9), the cohort of 4 fold-related channel proteins, gathered by dissectHMMER, helps to reaffirm that the 6-TM TIP12_MAIZE is indeed a channel protein, and in particular, an aquaporin.

In contrast, the last domain hit BCCT (PF02028.12) describes a 12-TM antiporter (concurrent exchange of carnitine and butyrobetaine) that is mechanistically different from a channel protein. This BCCT hit scores an unimpressive total FPR of 1.01. It is further ousted as a positive domain hit by its structural alignment results against 1YMG|A and scores a bad RMSD of 9.73 and a low structurally-derived %Id of 3.8. Meanwhile, it would have been difficult to identify the other less significant channel domains (i.e., Voltage_CLC, Form_Nir_tranp and ECF-ribofla_trS) via their original HMMER2 E-values since these values can span between a much less significant range of between 3.54e–3 and 3.89e–1 as compared to the very significant E-values of MIP at 1.46e–127. At the same time, the outlier antiporter domain (BCCT) with an E-value of 7.24e–2, complicates the analysis by sitting in the middle of the range. Meanwhile, using HMMER3, only two domains, MIP and Voltage_CLC were detected at E-values of 8.5e–73 and 6.52e–05, respectively.

In the last example, the dissectHMMER analysis of the uncharacterized 11-TM Q9K8K1_BACHD (sequence length of 388 AA) found 16 fold-related domain hits for further consideration (see Additional file 2: Table S4 and Additional file 8 for the full list). After filtering for domains with at least 10-TM, there remains 8 domain hits (out of which, 6 have representative PDB structures) on top of the most significant DUF819 domain hit, as listed in Table 4. And given the lack of function annotation from the most significant domain DUF819 (albeit having a total FPR = 0.0), the closest functional postulation for Q9K8K1_BACHD was proposed by the next most fold-critical significant MFS_1 domain (total FPR = 0.0) with a representative PDB structure 2CFP|A that describes a 12-TM lactose/H+ symporter. Furthermore, to clarify if Q9K8K1_BACHD can be generalized as a sugar transporter, the PDB structure 2CFP|A was used as a surrogate structure to Q9K8K1_BACHD for performing structural alignments (via the jCE algorithm [34]) against the representative PDB structures (if available) of the domain hits in Table 4. The resulting RMSDs, structurally-derived sequence identities (%Id), the aligned ranges from the structural alignments and the biological functions are tabulated in columns 6 and 7 of Table 4.

**Table 4:** Filtered dissectHMMER results from the analysis of the 11-TM Q9K8K1_BACHD

Domain description	Sequence range/Domain coverage	Original E-values [HMMER2/HMMER3]	[coverage/FPR]^ratio^ [coverage/FPR]^fc E-value^	Total FPR	RMSD/%Id/Structural alignment range (2CFP|A:pdb)	Function description of representative pdb
PF05684	10–388/	5.30e–244/	1.00/0.00	0.00	-	Unknown function
DUF819	1	3.50e–162	1.00/0.00			
length:400						
pdb:-						
PF07690.11	13–382/	7.72e–02/	0.845/0.00	0.00	0.0/	12-TM lactose permease (symporter) of E.coli that facilitates lactose and H+ translocation [57].
MFS_1	1	9.98e–05	0.815/0.00		100/	
length:793					1–417:	
pdb:2CFP|A					1–417	
PF00999.16	10–387/	8.25e–03/-	0.415/0.15	0.18	4.99/	12-TM sodium/proton (Na(+)/H(+)) antiporters [58].
Na_H_Exchanger	1		0.490/0.03		6.7/	
length:593					33–180:	
pdb:4BWZ|A					75–238	
PF13347.1	36–382/	8.31e–02/-	0.415/0.15	0.18	3.62/	12-TM glucose/H(+) symporter of Staphylococcus epidermidis [59]
MFS_2	1		0.490/0.03		9.4/	
length:847					11–401:	
pdb:4LDS|A					7–423	
PF00083.19	6–385/	5.20e–02/-	0.225/0.50	0.54	4.79/	12-TM D-xylose or d-glucose transporter [60]
Sugar_tr	1		0.485/0.04		6.5/	
length:605					4–388:	
pdb:4GC0|A					5–428	
PF00115.15	22–381/	7.88e–02/-	0.120/0.70	0.76	7.4/	12-TM mitochondrial cytochrome c oxidase that contains two proton pumps and a water channel [61].
COX1	1		0.475/0.06		1.7/	
length:591					9–417:	
pdb:1 V55|A					9–501	
PF03611.9	7–379/	1.28e–03/-	0.035/0.90	0.94	-	10-TM 3-keto-L-gulonate sugar-specific permease [62].
EIIC-GAT	1		0.485/0.04			
length:642						
pdb:-						
PF03169.10	3–388/	3.73e–02/-	0.050/0.88	0.98	-	12–14 TM oligopeptide transporter protein [63].
OPT	1		0.450/0.10			
length:1010						
pdb:-						
PF02028.12	7–330/	1.18e–02/-	0.005/1.00	1.01	7.73/	12-TM carnitine/butyrobetaine antiporter [56]
BCCT	1		0.500/0.01		3.1/	
length:722					8–212:	
pdb:2WSW|A					48–404	

For each domain hit, the Pfam accession, domain name, domain length and representative PDB (if any) are given in column 1. Column 2 gives the sequence range (i.e. sequence stretch covered by the domain) and the domain coverage where 1 indicates full coverage while <1 implies partial coverage by the domain model. Column 3 gives the original (or undissected) HMMER2 and HMMER3 E-values of the sequence-to-domain alignments. Column 4 gives the coverage score, coverage^ratio^ and coverage^fold ‐ critical E ‐ value^ (see Eq. 5) which is the corrected domain coverage score of the HMMER2/HMMER3 sequence-to-domain hit. The expected FPRs (false-positive rates) for the coverage scores are also provided and they were estimated from the relevant dissectHMMER ROC plots in Fig. 6. Column 5 gives the sorted total FPR in ascending order, where the latter is the sum of the two independent FPRs as given in column 3. Column 6 gives the RMSD/%Id and alignment range derived from the structure alignments between 2CFP|A and the representative structures of the domain models. The last column gives the biological function of the representative structures.

**Fig. 10:**
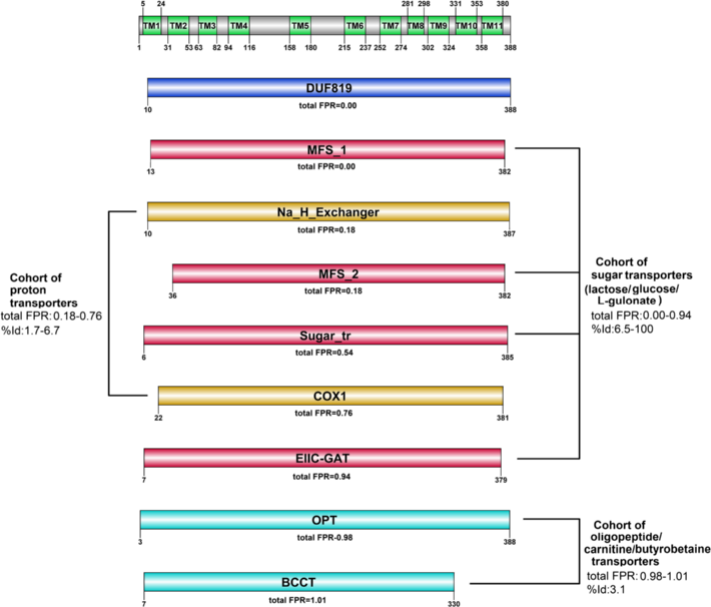
DissectHMMER analysis of Q9K8K1_BACHD. The sugar transporter group contains 4 independent domain hits to the sequence Q9K8K1_BACHD : MFS_1 (a 12-TM lactose/H+ symporter with a total FPR of 0.0), MFS_2 (a 12-TM glucose/H+ symporter with a total FPR of 0.18), Sugar_tr (a 12-TM d-xylose/d-glucose transporter with a total FPR of 0.54) and EIIC-GAT (10-TM L-gulonate sugar-specific transporter with a total FPR of 0.94). This fold-related cohort of sugar transporter domains spans a total FPR of between 0.00 and 0.94 and a structurally-derived %Id range of between 6.5 and 100 (good RMSD between 0 and 4.79). The next group is a cohort of proton transporters made up of the Na_H_Exchanger (a 12-TM sodium/H+ antiporter) and COX1 (a 12-TM dual proton pumps) domains. This group spans between a total FPR of between 0.18 and 0.76 with an unimpressive structurally-derived %Id of between 1.7 and 6.7 (bad RMSD range of between 4.99 and 7.4). Furthermore, the Na_H_Exchanger domain is an antiporter that is structurally different from the sugar symporters. And for the last group of peptide/amino-acid transporter, which is made up of the OPT and BCCT domain, it spans a total FPR of between 0.98 and 1.01 with a low %Id of 3.1. Taken together, Q9K8K1_BACHD is neither a proton transporter nor a peptide/amino-acid transporter given the low sequence identity and structural similarity. Rather, Q9K8K1_BACHD is a sugar transporter and particularly, a lactose/H+ symporter

Overall, the hits in Table 4 can be organized into three functional groups: the sugar transporters, the proton transporters and the peptide/amino-acid transporters as depicted in Fig. 10. The sugar transporter group contains 4 independent domain hits to the sequence Q9K8K1_BACHD : MFS_1 (a 12-TM lactose/H+ symporter with a total FPR of 0.0), MFS_2 (a 12-TM glucose/H+ symporter with a total FPR of 0.18), Sugar_tr (a 12-TM d-xylose/d-glucose transporter with a total FPR of 0.54) and EIIC-GAT (10-TM L-gulonate sugar-specific transporter with a total FPR of 0.94). This fold-related cohort of sugar transporter domains spans a total FPR of between 0.00 and 0.94 and has a structurally-derived sequence identity range of between 6.5 and 100 (good RMSD between 0 and 4.79). Notably, the changes in substrate specificity from lactose, glucose to L-gulonate in these sugar transporters are marked by the increasing total FPRs (0.0– > 0.18, 0.0– > 0.54, 0.0– > 0.94) as stratified by dissectHMMER, when the sequence Q9K8K1_BACHD deviates from the various distant sugar transporters.

The next group is a cohort of proton transporters made up of the Na_H_Exchanger (a 12-TM sodium/H+ antiporter) and COX1 (a 12-TM dual proton pumps) domains. This group spans between a total FPR of between 0.18 and 0.76 with an unimpressive structurally-derived %Id of between 1.7 and 6.7 (bad RMSD range of between 4.99 and 7.4). Furthermore, the Na_H_Exchanger domain is an antiporter that is structurally different from the sugar symporters. And for the last group of peptide/amino-acid transporter, which is made up of the OPT and BCCT domain, it spans a total FPR of between 0.98 and 1.01 with a low %Id of 3.1. Taken together, Q9K8K1_BACHD is neither a proton transporter nor a peptide/amino-acid transporter given the low sequence identity and structural similarity. Rather, the uncharacterized Q9K8K1_BACHD sequence is a 11-TM sugar transporter and particularly, a lactose/H+ symporter.

Based on the original HMMER2 results, the distinction among the sugar transporters (E-values between 1.28e–3 and 8.31e–2), the proton transporters (E-values between 8.25e–3 and 7.88e–2) and the peptide/amino-acid transporters (E-values between 1.18e–2 and 3.73e–2) would have been difficult due to the highly overlapping ranges. And based on the HMMER3 results with the detection of only the DUF819 and MFS_1 domains, conclusions would have been difficult. In this example, the steep jump in E-values between DUF819 and MFS_1 (HMMER2: 5.30e–244 versus 7.72e–02; HMMER3: 3.50e–162 versus 9.98e–5) would likely exclude the MFS_1 domain as a plausible hit, whereas dissectHMMER (via the total FPR measure) rescues it .

In hindsight, the vast range of the NADP-binding domains (HMMER2 E-values between 1.9e–67 and 4.86), channel proteins (HMMER2 E-values of between 1.46e–127 and 3.89e–1) and sugar transporters (HMMER2 E-values between 5.30e-244 and 8.31e-2; presume DUF819 is a sugar transporter domain) in the preceding case studies indicates a high level of sequence divergence within the various homologous groups. This, in turn, downplayed the statistical significance of some of these hits due to the penalty imposed by the non-globular segments, when evaluated over the full alignments.

Instead, by re-evaluating the fold-critical segments of the alignments through score dissection, dissectHMMER exhibits a deeper search depth and maintains a correct search path to elucidate more fold-related and relevant domain hits. And when these properly quantified and functionally-characterized hits are stratified into relevant cohorts, dissectHMMER is minimally able to postulate the generalized function of the query sequence. In the case of HEM1_METKA, its generalized biological function hints at cellular metabolism and specifically at an anabolic reaction as supported by the top hits of NADP+ binding domains (Shikimate_DH, F420_oxidored, 2-Hacid_dh_C, adh_short, NAD_binding_7 and NAD_binding_10 with total FPR ≤0.06). Meanwhile, for TIP12_MAIZE, it can be generalized as a channel pore that conducts some solute and particularly water/glycerol as supported by the MIP domain hit (with total FPR of 0.0). And for the case of the novel sequence Q9K8K1_BACHD, it can be generalized as a sugar transporter and is strongly postulated to be a lactose/H+ symporter as supported by the MFS_1 domain (total FPR of 0.0).

At the same time, the stratification of the quantified domain hits (via the ordered total FPR) helps to guide the amount or level of function transfer from the most significant domain hit to the sequence. To emphasize, the change in substrate specificity between the actual and next best domain hits (from NADP+ to NAD+ for HEM1_HETKA; from water to anions (Cl^-^,NO_2_^-^) for TIP12_MAIZE; from lactose to glucose for Q9K8K1_BACHD) occurs around the total FPR range of between 0.18 and 0.29. Anecdotally speaking, for cases where the total FPR of the most significant domain hit is below this range, the specific function may be inferred from this significant hit. However, if this most significant hit straddles around this range and beyond, the postulation of function should be limited to the level of the generalized biological function. As such, dissectHMMER attempts to balance between over and under-prediction of the biological function while transferring sufficient information to uncharacterized sequences that are currently not linked to any domain hits.

## Conclusions

The basis of the sequence homology concept states the necessity to emphasize on the similarity between the structural pieces of an alignment to ensure reasonable fold similarity (3D-structural) and, hence, the implied biological function. However, high sequence similarity (being the only surrogate measure to homology) does not necessarily imply homology. To add further complexity, current implementation of sequence search algorithms do not consciously differentiate between the 3D-structural sequence segments and the non-globular segments of the alignments. And hence, spurious yet statistically significant alignments can be propagated as homology pieces. The situation is made worse when one attempts to transfer function annotation between two distant sequences in the deeper sequence similarity search space since this implies higher sequence divergence that inevitably corrupts the homology signal.

The proposed framework, dissectHMMER, is built upon our previous work [19] in an attempt to break the limits of current sequence search algorithms (even outperforming a profile-to-profile based method-HHsuite/HHsearch) while maintaining on the correct search path. As fold similarity is the modus operandi of homology and that the fold is defined as the spatial arrangement of secondary structural elements [18], it is not surprising that searching with a query that primarily consists of sequences from the secondary structural elements (i.e., the fold-critical segments) is expected to be more successful. Overall, dissectHMMER is able to achieve a deeper yet relevant search depth through the rescue of statistically evaluated false-negative yet fold-related domain hits to the query sequence. In turn, the subsequent stratification of more fold-related hits (implying similar function) into cohorts of functionally-related domains allows dissectHMMER to minimally propose a generalized function for the query sequence when supported by biological evidence. The latter is crucial for many novel sequences whose current search space cannot be linked to any well-characterized protein sequences. Also, the stratification of the quantified hits via the ordered total FPR measures hints at the amount or level of function transfer from the most significant domain hit to the sequence. This allows a balance between over- and under-prediction of the function. Taken together, dissectHMMER presents an opportunity for current novel protein sequences to be functionally characterized, as illustrated through the real-life application of dissectHMMER on the sequence Q9K8K1_BACHD where it is resolved as a lactose/H+ symporter of the sugar transporter class.

## Methods

### Annotation of Pfam domain models into their fold-critical and remnant residues via a combination of sequence prediction tools that were calibrated using a set of PDB-Pfam mappings

Firstly, a list of Pfam-PDB mappings needs to be derived as a reference set. Based on the mapping file “pdb_pfam_mapping.txt” from the Pfam ftp site (ftp://ftp.ebi.ac.uk/pub/databases/Pfam/mappings/, Nov 2013), there is a total of 6863 Pfam to PDB associations. Using the initial mapping, hmmsearch (HMMER2) was performed for each of the 6863 Pfam (release 27) models against 249,830 PDB sequences extracted from the file “ss.txt” (http://www.rcsb.org/pdb/static.do?p=download/http/index.html, Jan 2014). In addition, the database size was corrected to *n* = 540,261 (UniProt as of April 2013) so that the E-values become more conservative. At E-value ≤1, there were 6599 Pfam-PDB mappings. In each Pfam-PDB mapping, the fold-critical residues (i.e. structural elements) and the remnant residues (i.e. loops, linkers etc.) can be easily defined using the associated DSSP information from the “ss.txt” file. The PDB-Pfam derived residue information serves as a reference to evaluate the performance of selected sequence tools for predicting the fold-critical residues (‘H’,‘E’,‘I’,‘T’,‘S’ based on DSSP annotation) and remnant residues (gaps ‘-’ or ‘?’) for the set of 6599 Pfam domains. These PDB/DSSP-derived Pfam domain annotations is given in Additional file 1.

Meanwhile for the prediction task, each Pfam (release 27) domain model will be converted into a score vector where each alignment position exhibits a score (i.e. a score-residue pair) that ranges between the values from 0 to 1 for each of the following predictors: the quality-score [20] (sequence conservation measure, parameter settings: BLOSUM62), PSIPred [21, 22] (secondary structure prediction, parameter settings:default), GlobPlot [24] (globular segment; smoothframe = 10, domjoinframe = 10, dompeakframe = 40, disjoinframe = 4, dispeakframe = 5) and SEG [23] (low-complexity measure; window size = 25, LOWcut = 3.1, HIGHcut = 3.4). Briefly, for the quality-score [20], the computations follows Eqs. 10–14 from [19] while, for the rest, the domain associated score vector can be computed using Eqs. 1–3 from [31]. It is important to note that for domains with less than 5 sequences, predictions by PSIPred [21, 22], GlobPlot [24] and SEG [23] will not be executed. As such, based on the same set of 6599 Pfam domains, the quality-score [20] would result in a total number of 1,403,175 score-residue pairs, while this is 1,290,923 score-residue pairs for the PSIPred [21, 22], GlobPlot [24] and SEG [23] predictors. In addition, remnant segments of less than 10 AA (amino acids) were considered as globular segments in both cases of PDB-derived and predicted residues.

To evaluate the performance of each of the predictors, a score threshold between 0 and 1 at interval of 0.05 was varied. Above a particular score threshold, a residue is considered as fold-critical while it is otherwise considered remnant. Hence, at each interval, the associated set of score-residue pairs was classified as true-positive (TP), false-positive (FP), false-negative (FN) and true-negative (TN) based on the PDB-derived residues as reference. Consequently, the false-positive rate (FPR) and true-positive rate (TPR) can be computed. The detailed results were tabulated in Additional file 2: Table S1.

Based on the Table, quality-score [20], PSIPred [21, 22], SEG [23] and GlobPlot [24] obtained their best predictive performance of (TPR-FPR) at 0.61, 0.50, 0.41 and 0.39 respectively (see entries marked ‘*’ in the last column). High TPR and low FPR gives the maximized margin. Evidently, the various predictors varied in their predictive power.

To combine the 4 predictor outputs into a single prediction outcome for each score-residue pair, a weighted-scoring scheme equation is given as follows:

1 $$ scor{e}_{weighted}={\displaystyle \sum_{i=1}^{N=4} scor{e}_{predicto{r}_i}\times {w}_{predicto{r}_i}} $$

where predictor = {qualityscore, PSIPred, SEG, GlobPlot} and *w*_qualityscore_ = 0.61, *w*_PSIPred_ = 0.50, *w*_SEG_ = 0.41 and *w*_GlobPlot_ = 0.39 respectively.

Then for each domain model, the score range is normalized to between 0 and 1 so that it is comparable to that of the predictors. This is achieved as follows:

2 $$ normscor{e}_{weighted,k}=\frac{scor{e}_{weighted,k}-{\alpha}_{\min }}{\alpha_{\max }-{\alpha}_{\min }} $$

where *α*_min_ = min{*score*_*weighted*,*k*_ : *k* = 1.. *K*}, *α*_max_ = max{*score*_*weighted*,*k*_ : *k* = 1.. *K*} and *k* is the alignment position of the domain model with length *K*.

Likewise, the performance of the weighted score was evaluated and the results were tabulated in Additional file 2: Table S2. At the score threshold of 0.5, the weighted-score gives one of the best predictive performance.

Finally, using the weighted-scoring scheme (i.e. combination of quality-score [20], PSIPred [21, 22], SEG [23] and GlobPlot [24]), the predictor-derived annotations for all 14,831 models of Pfam domain library were created and is given as Additional file 3.

### Creation of a SCOP superfamilies to Pfam domains mapping set

For the purpose of evaluating the error rates of the dissected fold-critical scores/E-values, a list of SCOP superfamily to Pfam domain mappings needs to be derived as the reference set. In SCOP, a superfamily contains at least one common ancestor protein within the group, hence the sequences within the group share fold similarity and are homologous among one another.

Up till SCOP release 1.75, protein sequences are subdivided into a hierarchical order of class (cl), fold (cf), superfamily (sf), family (fa) and domain(dm). Furthermore, between SCOP and PDB sequences, there is a 1:1 relationship that can be established since SCOP is a subset of the PDB database and herein, the SCOP-PDB entry. Based on the SCOP-PDB entry to SCOP classification file “dir.cla.scop.txt_1.75.txt” (http://scop.mrc-lmb.cam.ac.uk/scop/parse/) and its corresponding sequence file “astral-scopdom-seqres-gd-all-1.75.fasta” (http://scop.berkeley.edu/downloads/scopseq-1.75/astral-scopdom-seqres-gd-all-1.75.fa) from the SCOP website, one can construct a list of 1330 SCOP superfamilies (i.e. cl.cf.sf) with at least 2 sequences per superfamily after removing repeated sequences and substrings.

Then, for each of the 1330 SCOP superfamilies, the corresponding SCOP-PDB entries per superfamily were resolved against the mapping file “pdb_pfam_mapping.txt” from the Pfam ftp site (ftp://ftp.ebi.ac.uk/pub/databases/Pfam/mappings/, Nov 2013) using the common PDB|chain identifier so that the associated Pfam domains per SCOP superfamily could be associated. The list of SCOP superfamily to Pfam domains is provided as Additional file 9. From the list, each SCOP superfamily has an average of 16.5 sequences (standard deviation of 51.2) and can be mapped to an average of 4.8 Pfam domains (standard deviation of 10.0).

It is also noteworthy to mention that the number of mapped domain per SCOP superfamily tends to be optimistic. This is because the current version of “pdb_pfam_mapping.txt” file is computed using HMMER3 which operates only in local alignment mode, and hence, the Pfam domain might not fully cover the whole SCOP-PDB entry. Nevertheless, it should not affect our subsequent purpose of using the SCOP-to-Pfam mappings as a common baseline to evaluate any sequence search algorithms.

### Quantifying the false-positive rates of dissected fold-critical score associated measures using the SCOP superfamilies to Pfam domains mappings

Under the score dissection framework where both HMMER2 (in glocal mode) and HMMER3 (local mode only) algorithms were used, a query sequence can simultaneously generate both HMMER2-specific and HMMER3-specific, yet overlapping sequence-to-domain alignments. The alignments will be subjected to score reconstruction (based on Eqs. 1–2 from [19]) where it first recreates the alignment score by summing up the positional-dependent emission, transition log odd scores and invariant log odd scores, and then re-evaluates the sum for the corresponding E-value via the model’s EVD (extreme value distribution) statistical model. All the associated parameters (i.e. log-odd scores and summary statistic) are retrievable from the Pfam model files. When the score reconstruction is coupled to the dissection step, it allows the fold-critical and the remnant sum to be determined. The dissection step is guided by the residues’ information from the PDB/DSSP-derived Pfam domain annotations (see Additional file 1) that states if a residue is fold-critical or remnant. Consequently, the fold-critical and remnant E-values can also be derived from these sums through the model’s EVD statistical evaluation.

To quantify the false-positive rates associated to these fold-critical scores, two surrogate measures are derived : (i) the fold-critical E-value and (ii) the ratio of fold-critical E-value over remnant E-value. These measures were derived for both HMMER2 and HMMER3 over the 1330 SCOP superfamilies with mapped Pfam domains (see preceding section).

Potentially, each SCOP-PDB sequence within a superfamily can make a hit to any of the superfamily’s mapped Pfam domains. To create a hit list, each sequence in a SCOP superfamily is searched via hmmpfam (for HMMER2) or hmmscan (for HMMER3) against the full Pfam domain library (version 27; 14,831 models) at an E-value cutoff of 20. These domain hits were then checked against the superfamily’s mapped list of Pfam domains (see Additional file 9) and then classified accordingly as positives (if overlapping) or negatives (if non-overlapping). Since we are interested only in quantifying the false-positive rates, only the negative domains hit were subjected to the score/E-value reconstruction and dissection as described earlier.

In summary, the computations over 22,001 SCOP sequences from the 1330 SCOP superfamilies will result in 136,642 HMMER2 and 41,506 HMMER3 fold-critical E-values for the negative sets. This will also correspond to 136,645 HMMER2 and 41,489 HMMER3 ratios (i.e. fold-critical E-values versus remnant E-values) for the negative sets respectively. The total numbers in the ratio sets are slightly smaller than the fold-critical sets since ratios with remnant E-values of zero were excluded (i.e. ratios would have been infinity).

**Fig. 11:**
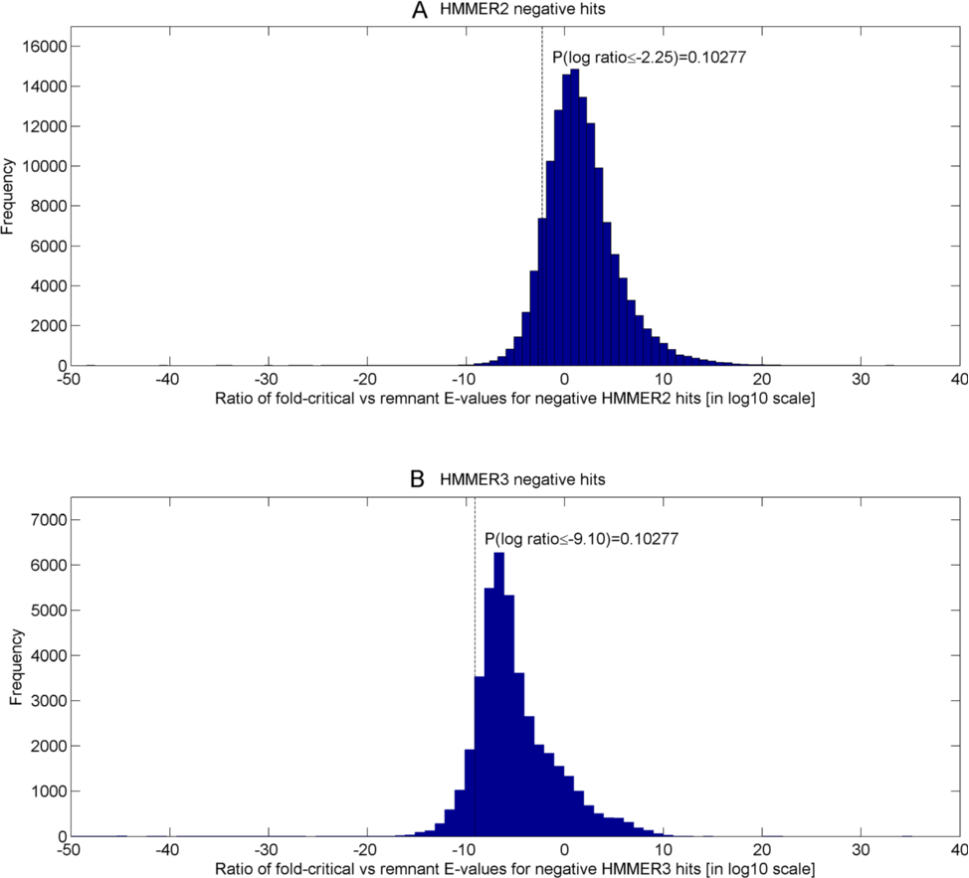
Histograms of fold-critical over remnant E-value ratios for the negative HMMER2 and HMMER3 hits respectively. Given the ratio between fold-critical versus remnant E-values of 0.056 (10^–2.25^), the corresponding FPR for HMMER2 is found to be about 0.1 (see dotted line in Fig. 11a). Correspondingly for the same FPR (see dotted line in Fig. 11b, this ratio for HMMER3 is preset at a much smaller value of 7.9e–10 (10^−9.1^)

**Fig. 12:**
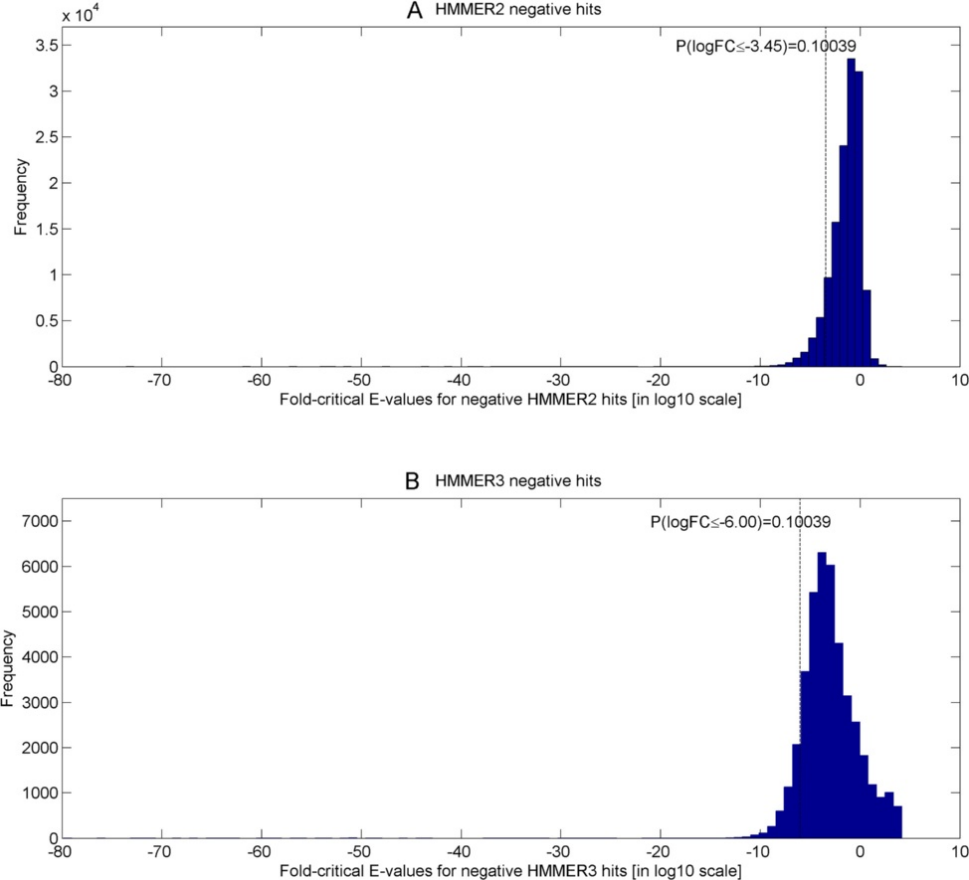
Histograms of fold-critical E-values for the negative HMMER2 and HMMER3 hits respectively. At a fold-critical E-value of 3.6e–4 (10^−3.45^), the FPR is found to be approximately 0.1 (see *dotted line*) for HMMER2 in Fig. 12a. Meanwhile, the fold-critical E-value is set at 1.0e–6 (10^−6^) for the same FPR for HMMER3 based on Fig. 12b (see *dotted line*)

Figures 11 depicts the histograms of fold-critical over remnant E-value ratios for the negative HMMER2 (Fig. 11a) and HMMER3 (Fig. 11b) hits respectively. Similarly, Figs. 12 depicts the histograms of fold-critical E-values for the negative HMMER2 (Fig. 12a) and HMMER3 (Fig. 12b) hits respectively. Based on the histograms of these negative data sets, for a given cutoff (whether a ratio or fold-critical E-value; see dotted lines as example), the area on the left-hand side constitutes the false-positive rate. Computationally, the false-positive rate (FPR) is the summation of the frequencies from the left most extreme interval to the k^th^ interval containing the cutoff value, x as follows:

3 $$ fp{r}_{measure}=P\left( measure\le x\right)={\displaystyle \sum_{i= \min\ \mathrm{interval}}^{\mathrm{x}\in \mathrm{interval}\ \mathrm{k}} frequenc{y}_i} $$

where measure is either a ratio (i.e. fold-critical E-value/remnant E-value) or a fold-critical E-value in log10 scale, frequency_i_ is the height of interval i, x is the observed ratio or fold-critical E-value to be used as the cutoff. Essentially, *P*(*measure* ≤ *x*) denotes the chances of seeing a value smaller than the observed value (i.e. x) under the null hypothesis that assumes all sample observations arise by chance.

As an example, given the ratio between fold-critical versus remnant E-values of 0.056 (10^−2.25^), the corresponding FPR for HMMER2 is found to be about 0.1 (see dotted line in Fig. 11a). Correspondingly for the same FPR (see dotted line in Fig. 11b, this ratio for HMMER3 is preset at a much smaller value of 7.9e-10 (10^−9.1^). Similarly, based on Fig. 12a, at a fold-critical E-value of 3.6e–4 (10^−3.45^), the FPR is found to be approximately 0.1 (see dotted line) for HMMER2. Meanwhile, the fold-critical E-value is set at 1.0e–6 (10^−6^) for the same FPR for HMMER3 based on Fig. 12b (see dotted line).

### Classification criteria of sequence-to-domain alignment hits

In the preceding section, the computations of 22,001 SCOP sequences from the 1330 SCOP superfamilies has resulted in 136,642 HMMER2 and 41,506 HMMER3 fold-critical E-values for the negative hits. And using Eq. 3, the false-positive rates of both HMMER variants associated to the negative fold-critical E-values at each threshold can be determined. For both HMMER2 and HMMER3 negative sets, the threshold is varied between 10^−205^ and 10^3^. The resulting false-positive rates for both HMMER variants is provided in Additional file 5.

To create the criteria to classify each sequence-to-domain hit as true-positive (TP), false-negative (FN), false-positive (FP) and true-negative (TN), the cutoff values have to be determined for the (i) original E-value (i.e. the original undissected alignment) and the (ii) fold-critical E-value.

For the original E-value, the reference is taken from the recommended HMMER2 E-value for trusted hits [32] at 0.1 (false-positive rate of 0.53). This is matched by a HMMER3 E-value of 10^−3^ (false-positive rate of 0.55). For the fold-critical E-value, the HMMER2 and HMMER3 values are 10^–3.45^ and 10^−6^ respectively. These values has an associated false-positive rate of 0.1. As a result, the criteria for the hit classification are summarized in Table 1.

### Error-adjusted domain coverage score: combining HMMER2 and HMMER3 fold-critical measures and domain coverage

In the preceding section, the false-positive rate of either HMMER2’s or HMMER3’s hit can be empirically computed when given the hit’s surrogate measures of either fold-critical over remnant E-value ratio or fold-critical E-value. However, due to the algorithmic, parameterization, alignment mode differences between the HMMER variants, the two derived false-positive rates may not be necessarily comparable for overlapping HMMER2 and HMMER3 sequence-to-domain alignments.

To emphasize, overlapping denotes some coverage of common sequence ranges between the two alignments for the same domain model. In particular, an overlap ratio, *overlap*_*ratio*_ between the two comparable sequence-to-domain alignments where sequence segment i precedes segment j, can be defined as follows :

4 $$ overla{p}_{ratio}= \min \left(\frac{b_i-{a}_j}{b_i-{a}_i},\frac{b_i-{a}_j}{b_j-{a}_j}\right) $$

where (*a*_*i*_, *b*_*i*_) is the start and end positions of sequence segment i, (*a*_*j*_, *b*_*j*_) is the start and end positions of sequence segment j.

Aside that, it is also insufficient for a sequence-to-domain hit to solely achieve some significant fold-critical E-values or low fold-critical over remnant E-values ratio. Concurrently, the hit should exhibit some reasonable level of domain coverage to indicate that the sequence has an overall fold similarity to the domain. Taken together, this necessitates for a scoring scheme that reflects both the statistical results and the domain coverage in the overlapping sequence-to-domain hit of HMMER2 and HMMER3 as a singular measure. As such, an error-adjusted domain coverage measure is proposed as follows :

5 $$ {\mathrm{coverage}}^{\mathrm{measure}}=\frac{1}{2}\left[\left(1-fp{r}_{\mathrm{measure}}^{\mathrm{HMMER}2}\right){\mathrm{coverage}}^{\mathrm{HMMER}2}+\left(1-fp{r}_{\mathrm{measure}}^{\mathrm{HMMER}3}\right){\mathrm{coverage}}^{\mathrm{HMMER}3}\right] $$

where measure is either a ratio (i.e. fold-critical E-value/remnant E-value) or a fold-critical E-value in log10 scale, fpr is the empirically-derived false-positive rate associated to the measure for either HMMER2 or HMMER3, coverage is the alignment length of sequence-to-domain hit over the domain length for either HMMER2 or HMMER3.

Note that for HMMER2, the coverage is always 1 since glocal mode is enforced while coverage for HMMER3 is ≤1. As such, the range of values for coverage_measure_ is between 0 and 1.

### SCOP superfamily-wise evaluation of dissectHMMER

For each SCOP superfamily with N sequences, a multiple sequence alignment (MSA) of length L can be created using the clustalw program as follows :

$$ \begin{array}{cccccc}\hfill {s}_{11}\hfill & \hfill {s}_{12}\hfill & \hfill \dots \hfill & \hfill {s}_{1j}\hfill & \hfill \dots \hfill & \hfill {s}_{1L}\hfill \\ {}\hfill {s}_{21}\hfill & \hfill {s}_{22}\hfill & \hfill \dots \hfill & \hfill {s}_{2j}\hfill & \hfill \dots \hfill & \hfill {s}_{2L}\hfill \\ {}\hfill \vdots \hfill & \hfill \vdots \hfill & \hfill \ddots \hfill & \hfill \vdots \hfill & \hfill \vdots \hfill & \hfill \vdots \hfill \\ {}\hfill {s}_{i1}\hfill & \hfill {s}_{i2}\hfill & \hfill \dots \hfill & \hfill {s}_{ij}\hfill & \hfill \dots \hfill & \hfill {s}_{iL}\hfill \\ {}\hfill \vdots \hfill & \hfill \vdots \hfill & \hfill \ddots \hfill & \hfill \vdots \hfill & \hfill \vdots \hfill & \hfill \vdots \hfill \\ {}\hfill {s}_{N1}\hfill & \hfill {s}_{N2}\hfill & \hfill \dots \hfill & \hfill {s}_{Nj}\hfill & \hfill \dots \hfill & \hfill {s}_{NL}\hfill \end{array} $$

where S_ij_ is the amino acid alphabet or a gap, i is the index of the sequence and j is the alignment position.

For each sequence, dissectHMMER will be applied to generate the sequence-to-domain alignment hits and the associated error-adjusted domain coverage mesasures (for both ratio and fold-critical E-value measures; see Eq. 5).

For the set of generated sequence-to-domain alignments, individual hits belonging to the i^th^ sequence and k^th^ Pfam domain model, ${\left[ sequenc{e}_i-\mathrm{t}\mathrm{o}- domai{n}_k\right]}_{a_i{b}_i}$ are gathered as a group, where a_i_b_i_ denotes the start and end position of the sequence range that is mapped to the MSA alignment positions.
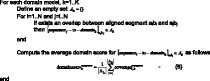


## Reviewers’ comments

### Reviewer’s report 1: Masanori Arita, Metabolome Informatics Research Team, Metabolomics Research Group, RIKEN Center for Sustainable Resource Science

This manuscript is a completion work by the authors regarding the score dissection of HMMER software tools. The concept is reasonable and the improvement is expected (meta tools with dissection analysis usually perform better). Nevertheless, the obtained results are quite notable as in Fig. 6. The improvement is significant and I strongly agree with authors for its worth.

In the background section, it is better to explain the difference between HMMER2 and HMMER3 because their difference is crucial in the analysis. Explanation of the glocal mode and the limitation of HMMER3 because of its statistical model greatly helps readers to understand the merits of using both tools other than speed.

Authors’ response: *We thank the reviewer for his kind comments on our work. We have added some explanation in the background section to better explain the rationale of using both HMMER2 and HMMER3*.

The performance comparison with HHsearch needs more care: although tools were compared on 1199 SCOP families, a part of them could only create marginal HMM representation due to the family size. I would like to know the performance by using large families only, because the number of families for comparison needs not be as much as 1000. If there is a performance difference depending on fold superfamilies, that would also provide important insights for readers.

Authors’ response: *We thank the reviewer for his critical consideration. In fact*, *the issue is slightly more involved. Out of the 131 SCOP superfamilies that were unable to generate HHsearch profiles*, *125 of them indeed contain 6 or less sequences. At the same time, 6 other SCOP superfamilies have between 11 and 1318 sequences. While insufficient sequences can degenerate the quality of a HMM profile, the multiple sequence alignment of a large superfamily can also be challenging at the same time. In hindsight, the quality of the multiple sequence alignment is also an important factor that contributes to the entropy of a HMM profile, especially for profile-to-profile based methods like HHsearch.*

**Fig. 13:**
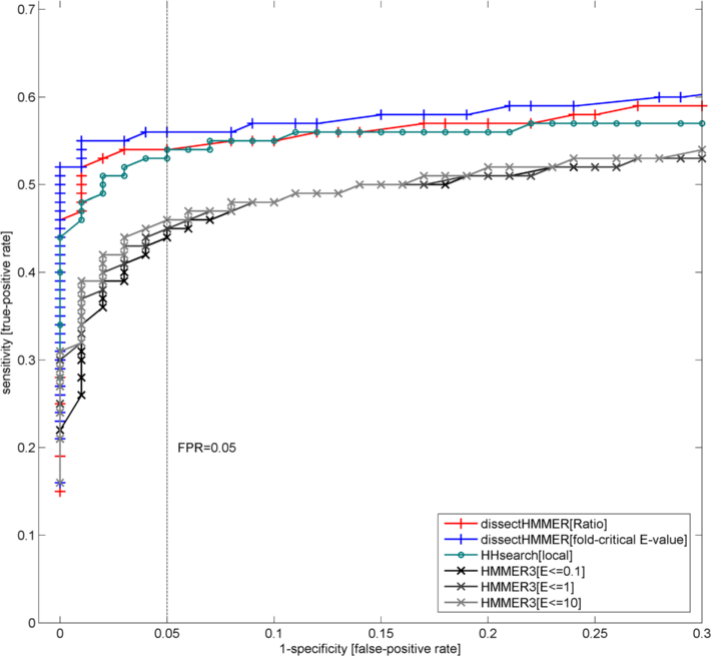
ROC (receiver operator curve) plots for dissectHMMER, HHsearch and HMMER3 against selected 1134 SCOP-to-Pfam mapped superfamily cases. The top 5 % (65 out of 1199) of SCOP superfamilies with large alignments (i.e. the number of sequences >60) were excluded to maximize the results of HHsearch. Compared to Fig. 6, all individual plots (dissectHMMER, HHsearch, HMMER3) has improved sensitivity (i.e. TPR) for the same specificity rate. The plot for HHsearch improved dramatically and almost comparable to that of dissectHMMER[Ratio]. However, at a FPR of ≤0.05, dissectHMMER remains more sensitive than HHsearch. As such, the results reinforced that score dissection can improve fold similarity detection in existing methods, namely, HMMER3, and comparable to a profile-to-profile search method like HHsearch

*To illustrate the point, the top 5 % (65 out of 1199) of SCOP superfamilies with large alignments (i.e. the number of sequences >60) were excluded while the attempt to exclude superfamilies with low number of sequences does not further improve the HHsearch plot (data not shown). Overall, the performance comparison for 1134 SCOP superfamilies is depicted in Fig.*13*.*

*Compared to Fig.*6*, all individual plots (dissectHMMER, HHsearch, HMMER3) were generally shifted towards better sensitivity (i.e. TPR) with the plot of HHsearch being dramatically improved and almost comparable to the dissectHMMER[Ratio] plot. Despite so, at a FPR of ≤0.05, dissectHMMER remains more sensitive than HHsearch, given the latter’s best performance in this comparison. As such, the results again reinforced that score dissection can improve fold similarity detection in existing methods, namely, HMMER3, and comparable to a profile-to-profile search method like HHsearch.*

Minor points:

Page 3 first paragraph: The word “inductive proof” is too strong a word. Since this is not a mathematics paper I would suggest a softer language than a proof.

Page 12 2nd paragraph: “for better or worst” --> worse?

Page 13 2nd paragraph: “profile-to-profile HMMER method” --> “profile-to-profile HMM method”

Page 19 2nd last paragraph: “dissecthmmer” --> “dissectHMMER”

Authors’ response: *The minor points have all been addressed in the revised manuscript.*

### Reviewer’s report 2: Shamil Sunyaev, Division of Genetics, Dept. of Medicine, Brigham & Women’s Hospital and Harvard Medical School

This manuscript offers a framework to evaluate remote homology by focusing on positions critical to protein folds. It combines HMMER search with methods for predicting disordered regions. The method has been successfully validated and implemented as an online tool. This development is of interest and the tool is potentially useful. As a minor comment, the paper can be written in a more economical and focused way. A shorter title may also be better.

Authors’ response: *We thank the reviewer for his kind comments and we have shortened the title. Though the manuscript’s main aim is to provide an improved search tool to evaluate homology, the rationale behind dissectHMMER also needs to be sufficiently justified in the manuscript. As such, the existing text is considered polished and tightened.*

### Reviewer’s report 3: L. Aravind, Protein and Genome Evolution Research Group, Computational Biology Branch, NCBI/NLM/NIH

The paper develops a concept worked out by the authors that the “dissection” of positions into fold critical versus non-globular contributions improves judgement of homology and thereby function prediction. In conceptual terms this proposal might be viewed in two potentially opposing ways: On one hand, the separation of the fold-critical and non-globular parts in the domain model is expected to reduce “false signal” of homology that tends to arise from non-globular regions. This is favorably reflected in the benchmarking experiments presented here by the authors. Indeed, the directed use of the fold-critical parts of the model seem to enhance recognition of correct structural relationship. On the other hand it should be kept in mind that natural selection does not entirely respect the fold-critical-non-globular dichotomy with potentially functionally critical constellations of residues being lodged in regions deemed non-globular. These can provide specific functional information that goes over and beyond the basic fold-recognition, and in some cases tend to be strongly conserved. Hence, the removal of such can potentially hamper function inference. The authors might hence want to clearly specify how this might affect their approach.

Authors’ response: *The reviewer has highlighted an important issue. We agree that the well-conserved non-globular segments might contain useful functional annotations like the cases of many diverse PTM sites/motifs. However, fold similarity (via fold-critical segments) implies an overall emphasize over the full-length domain scoring whereas functional sites/motifs are small and localized signals that covers only part of the larger domain model. As such, the latter remains complementary for further clarifying sequence-to-domain hits that have similar fold-critical scores. But alone, it is likely to attract sequence-to-domain hits of unrelated biological functions, which is the bane of the problem in functional annotation.*

*In practice, dissectHMMER considers short non-globular aligned segments of ≤10 alignment positions as part of the fold-critical segments. These segments are generally well-conserved in the multiple sequence alignment (See methods: Annotation of Pfam domain models…) yet they are in minority. Mostly, the non-globular alignment segments in domain models have many gaps (due to varying inter-linkers lengths) and the supposedly conserved pieces are also not well-aligned. The latter is largely responsible for the spurious sequence-to-domain hits and the situation is made worse when used with the local-mode only HMMER3. To properly capture the non-globular functional sites/motifs in each domain model, the sites/motifs need to be detected via specific PTM predictors and then to construct some well-conserved alignment out of them. Finally, only part of the overall gapped alignment that captures the well-conserved pieces should be considered as part of the fold-critical segments. However, this is beyond the scope of the current work.*

The second aspect of this work pertains to functional inference from fold recognition. Here the key process followed by the authors is the stratification of hits in clusters of related domains. The biological evidence can be of two types:

1) a biochemical function which can have differing levels of precision, like “methylase” or “methylase catalyzing methylation of particular substrate”, etc. and

2) general biological function like “epigenetic modification”. It is not clear how these are distinguished in during the creation of cohorts during analysis of the hits. The former tends to be much more informative in terms of function prediction:

Authors’ response: *Historically, most domain libraries (e.g. SMART and Pfam) started with the collection of models that describe enzymatic/biochemical function and hence characterized by folds (as evident in the early versions of domain libraries). In contrast, biological function like* “*epigenetic modification*” *describes the more localized functional annotations of the models. dissectHMMER works towards detecting sequence-to-domain hits that shares good fold similarity to infer function of the first type. Adding the extra description of localized functional annotations of the second type (as our future work) will help to further segregate the results into more defined sub-cohorts.*

Minor:

-The results in the abstract are presented in the past tense: it might be stylistically better to use the present tense to distinguish current work from previously published work.

-Several figures were invisible in the PDF version for review making some of the statements hard to visualize

-Regretfully - > “Regrettably” would make better sense

-DUF819 is called PF05684.7 but in Pfam it is just PF05684.

Authors’ response: *The preceding minor points have all been addressed in the revised manuscript.*

-DUF819 belongs in “Clan” with several other transported which could be used for functional inference though by itself it is a DUF.

Authors’ response: *Although DUF819 belongs to the CPA superfamily (clan: CL0064) and is suggestive of transporter function, its specific function is more difficult to clarify this way.*

## Additional files

Additional file 1:
**Annotations of fold-critical and remnant residues of a reference set of 6599 Pfam domains with PDB/DSSP information.** (TXT 3708 kb) Additional file 2:
**Table S1.** Sensitivity and specificity of each of the predictors (i.e. quality-score [20], PSIPred [21, 22], SEG [23] and GlobPlot [24]) against a reference set of 6599 Pfam domains with PDB/DSSP information. **Table S2.** Sensitivity and specificity of the weighted-scoring scheme against a reference set of 6599 Pfam domains with PDB/DSSP information. **Table S3.** Full list of dissectHMMER results for the analysis of TIP12_MAIZE. **Table S4.** Full list of dissectHMMER results for the analysis of Q9K8K1_BACHD. (DOC 266 kb) Additional file 3:
**Predicted annotations of fold-critical residue in the 14,831 Pfam domains using the weighted-scoring scheme.** (TXT 4429 kb) Additional file 4:
**The hmmfam results of 8 PDB/DSSP-derived domain models aligned to non-globular region of their associated PDB sequence/DSSP information, implying that these models have no fold-critical residues.** (TXT 9 kb) Additional file 5:
**False–positive rates of the fold-critical E-values computed for the sets of HMMER2 and HMMER3 negative hits over the 1330 SCOP superfamilies set.** (TXT 8 kb) Additional file 6:
**The web output of dissectHMMER search results for the protein sequence HEM1_METKA.** (ZIP 9 kb) Additional file 7:
**The web output of dissectHMMER search results for the protein sequence TIP12_MAIZE.** (ZIP 9 kb) Additional file 8:
**The web output of dissectHMMER search results for the protein sequence Q9K8K1_BACHD.** (ZIP 19 kb) Additional file 9:
**Mapping of each of the 1330 SCOP superfamilies to its associated Pfam domain models.** (TXT 358 kb)

## Abbreviations

HMM: Hidden Markov model
ROC: Receiver operator’s curve
TPR: True positive rate
FPR: False positive rate
AA: Amino acids
