# Supplementary material for: dissectHMMER: a HMMER-based score dissection framework that statistically evaluates fold-critical sequence segments for domain fold similarity
Source: Biol Direct. 2015 Aug 1;10:39. doi: 10.1186/s13062-015-0068-3 (PMC4521371; doi:10.1186/s13062-015-0068-3)
Supplement: Additional file 2: — Table S1. Sensitivity and specificity of each of the predictors (i.e. quality-score [20], PSIPred [21, 22], SEG [23] and GlobPlot [24]) against a reference set of 6599 Pfam domains with PDB/DSSP information. Table S2. Sensitivity and specificity of the weighted-scoring scheme against a reference set of 6599 Pfam domains with PDB/DSSP information. Table S3. Full list of dissectHMMER results for the analysis of TIP12_MAIZE. Table S4. Full list of dissectHMMER results for the analysis of Q9K8K1_BACHD. (DOC 266 kb) [file 13062_2015_68_MOESM2_ESM.doc]

**Additional file 2**

**Table S1**

**Sensitivity and specificity of each of the predictors (i.e. quality‑score [1], PSIPred [2,3], SEG [4] and GlobPlot [5]) against a reference set of 6599 Pfam domains with PDB/DSSP information.**

To evaluate the performance in terms of sensitivity (i.e. true‑positive rate) and specificity (i.e. false‑positive rate) for each of the predictors, they were benchmarked against a reference set of 6599 Pfam domains with PDB/DSSP information (see additional file 1). The respective true‑positive rate (TPR) and false‑positive rate (FPR) at each threshold level are provided in the table below for each predictor (quality‑score, PSIPred, SEG, GlobPlot) separately. Note that FPR, TPR, TP, FN, FP, TN denote false‑positive rate, true‑positive rate, true positive, false negative, false positive and true negative respectively. The score performance is measured by the difference (TPR‑FPR) between the true‑positive rate (TPR) and false‑positive rate (FPR) at each threshold level. The optimal performance is denoted by '*'.

| quality‑score: | | | | | | | | | | | | | | | |  |
| --- | --- | --- | --- | --- | --- | --- | --- | --- | --- | --- | --- | --- | --- | --- | --- | --- |
| Threshold | FPR | TPR | TP | | FN | | FP | | | TN | | | TPR‑FPR | | |  |
| 0.05 | 0.59 | 0.99 | 1071575 | | 6382 | | 190908 | | | 134310 | | | 0.40 | | |  |
| 0.10 | 0.50 | 0.99 | 1066597 | | 11360 | | 161695 | | | 163523 | | | 0.49 | | |  |
| 0.15 | 0.45 | 0.99 | 1062228 | | 15729 | | 146757 | | | 178461 | | | 0.54 | | |  |
| 0.20 | 0.42 | 0.98 | 1057457 | | 20500 | | 136963 | | | 188255 | | | 0.56 | | |  |
| 0.25 | 0.40 | 0.98 | 1051024 | | 26933 | | 129121 | | | 196097 | | | 0.58 | | |  |
| 0.30 | 0.38 | 0.97 | 1042432 | | 35525 | | 122326 | | | 202892 | | | 0.59 | | |  |
| 0.35 | 0.35 | 0.95 | 1028911 | | 49046 | | 114492 | | | 210726 | | | 0.60 | | |  |
| 0.40 | 0.32 | 0.93 | 1006341 | | 71616 | | 105295 | | | 219923 | | | 0.61* | | |  |
| 0.45 | 0.29 | 0.90 | 968851 | | 109106 | | 94791 | | | 230427 | | | 0.61* | | |  |
| 0.50 | 0.25 | 0.85 | 912191 | | 165766 | | 82806 | | | 242412 | | | 0.60 | | |  |
| 0.55 | 0.22 | 0.78 | 840232 | | 237725 | | 70995 | | | 254223 | | | 0.56 | | |  |
| 0.60 | 0.19 | 0.70 | 759506 | | 318451 | | 60794 | | | 264424 | | | 0.51 | | |  |
| 0.65 | 0.16 | 0.63 | 677773 | | 400184 | | 52397 | | | 272821 | | | 0.47 | | |  |
| 0.70 | 0.14 | 0.55 | 596490 | | 481467 | | 44880 | | | 280338 | | | 0.41 | | |  |
| 0.75 | 0.12 | 0.48 | 512935 | | 565022 | | 38422 | | | 286796 | | | 0.36 | | |  |
| 0.80 | 0.10 | 0.40 | 426588 | | 651369 | | 32292 | | | 292926 | | | 0.30 | | |  |
| 0.85 | 0.08 | 0.32 | 349874 | | 728083 | | 27364 | | | 297854 | | | 0.24 | | |  |
| 0.90 | 0.07 | 0.27 | 287514 | | 790443 | | 23467 | | | 301751 | | | 0.20 | | |  |
| 0.95 | 0.06 | 0.22 | 240141 | | 837816 | | 20894 | | | 304324 | | | 0.16 | | |  |
| PSIPred: | | | | | | | | | | | | | | |  | |
| Threshold | FPR | TPR | TP | FN | | FP | | | TN | | | TPR‑FPR | | |  | |
| 0.05 | 0.18 | 0.68 | 663672 | 317977 | | 56440 | | | 252834 | | | 0.50* | | |  | |
| 0.10 | 0.18 | 0.68 | 663672 | 317977 | | 56440 | | | 252834 | | | 0.50* | | |  | |
| 0.15 | 0.18 | 0.68 | 663672 | 317977 | | 56440 | | | 252834 | | | 0.50* | | |  | |
| 0.20 | 0.18 | 0.68 | 663672 | 317977 | | 56440 | | | 252834 | | | 0.50* | | |  | |
| 0.25 | 0.18 | 0.68 | 663672 | 317977 | | 56440 | | | 252834 | | | 0.50* | | |  | |
| 0.30 | 0.18 | 0.68 | 663672 | 317977 | | 56440 | | | 252834 | | | 0.50* | | |  | |
| 0.35 | 0.18 | 0.68 | 663672 | 317977 | | 56440 | | | 252834 | | | 0.50* | | |  | |
| 0.40 | 0.18 | 0.68 | 663672 | 317977 | | 56440 | | | 252834 | | | 0.50* | | |  | |
| 0.45 | 0.18 | 0.68 | 663672 | 317977 | | 56440 | | | 252834 | | | 0.50* | | |  | |
| 0.50 | 0.18 | 0.68 | 663672 | 317977 | | 56440 | | | 252834 | | | 0.50* | | |  | |
| 0.55 | 0.18 | 0.68 | 663286 | 318363 | | 56380 | | | 252894 | | | 0.50* | | |  | |
| 0.60 | 0.18 | 0.67 | 659710 | 321939 | | 55677 | | | 253597 | | | 0.49 | | |  | |
| 0.65 | 0.17 | 0.66 | 650858 | 330791 | | 54091 | | | 255183 | | | 0.49 | | |  | |
| 0.70 | 0.17 | 0.65 | 638760 | 342889 | | 51491 | | | 257783 | | | 0.48 | | |  | |
| 0.75 | 0.15 | 0.63 | 621406 | 360243 | | 47935 | | | 261339 | | | 0.48 | | |  | |
| 0.80 | 0.14 | 0.61 | 597016 | 384633 | | 42246 | | | 267028 | | | 0.47 | | |  | |
| 0.85 | 0.12 | 0.58 | 570794 | 410855 | | 37497 | | | 271777 | | | 0.46 | | |  | |
| 0.90 | 0.10 | 0.54 | 532226 | 449423 | | 32147 | | | 277127 | | | 0.44 | | |  | |
| 0.95 | 0.09 | 0.49 | 481470 | 500179 | | 27301 | | | 281973 | | | 0.40 | | |  | |
| SEG: | | | | | | | | | | | | | | |  | |
| Threshold | FPR | TPR | TP | FN | | FP | | | TN | | | TPR‑FPR | | |  | |
| 0.05 | 0.47 | 0.88 | 863209 | 118440 | | 145911 | | | 163363 | | | 0.41* | | |  | |
| 0.10 | 0.47 | 0.88 | 863209 | 118440 | | 145911 | | | 163363 | | | 0.41* | | |  | |
| 0.15 | 0.47 | 0.88 | 863209 | 118440 | | 145911 | | | 163363 | | | 0.41* | | |  | |
| 0.20 | 0.47 | 0.88 | 863209 | 118440 | | 145911 | | | 163363 | | | 0.41* | | |  | |
| 0.25 | 0.47 | 0.88 | 863209 | 118440 | | 145911 | | | 163363 | | | 0.41* | | |  | |
| 0.30 | 0.47 | 0.88 | 863209 | 118440 | | 145911 | | | 163363 | | | 0.41* | | |  | |
| 0.35 | 0.47 | 0.88 | 863209 | 118440 | | 145911 | | | 163363 | | | 0.41* | | |  | |
| 0.40 | 0.47 | 0.88 | 863209 | 118440 | | 145911 | | | 163363 | | | 0.41* | | |  | |
| 0.45 | 0.47 | 0.88 | 863209 | 118440 | | 145911 | | | 163363 | | | 0.41* | | |  | |
| 0.50 | 0.47 | 0.88 | 863209 | 118440 | | 145911 | | | 163363 | | | 0.41* | | |  | |
| 0.55 | 0.47 | 0.88 | 862593 | 119056 | | 145857 | | | 163417 | | | 0.41* | | |  | |
| 0.60 | 0.47 | 0.87 | 856983 | 124666 | | 145080 | | | 164194 | | | 0.40 | | |  | |
| 0.65 | 0.46 | 0.86 | 841478 | 140171 | | 142262 | | | 167012 | | | 0.40 | | |  | |
| 0.70 | 0.44 | 0.83 | 811320 | 170329 | | 136171 | | | 173103 | | | 0.39 | | |  | |
| 0.75 | 0.41 | 0.77 | 759340 | 222309 | | 125506 | | | 183768 | | | 0.36 | | |  | |
| 0.80 | 0.35 | 0.69 | 678491 | 303158 | | 106929 | | | 202345 | | | 0.34 | | |  | |
| 0.85 | 0.28 | 0.59 | 576821 | 404828 | | 87227 | | | 222047 | | | 0.31 | | |  | |
| 0.90 | 0.20 | 0.44 | 434661 | 546988 | | 60960 | | | 248314 | | | 0.24 | | |  | |
| 0.95 | 0.13 | 0.29 | 284523 | 697126 | | 39843 | | | 269431 | | | 0.16 | | |  | |
| GlobPlot: | | | | | | | | | | | | | | | | |
| Threshold | FPR | TPR | TP | | FN | | | FP | | | TN | | | TPR‑FPR | | |
| 0.05 | 0.47 | 0.86 | 840999 | | 140650 | | | 144343 | | | 164931 | | | 0.39* | | |
| 0.10 | 0.47 | 0.86 | 840999 | | 140650 | | | 144343 | | | 164931 | | | 0.39* | | |
| 0.15 | 0.47 | 0.86 | 840999 | | 140650 | | | 144343 | | | 164931 | | | 0.39* | | |
| 0.20 | 0.47 | 0.86 | 840999 | | 140650 | | | 144343 | | | 164931 | | | 0.39* | | |
| 0.25 | 0.47 | 0.86 | 840999 | | 140650 | | | 144343 | | | 164931 | | | 0.39* | | |
| 0.30 | 0.47 | 0.86 | 840999 | | 140650 | | | 144343 | | | 164931 | | | 0.39* | | |
| 0.35 | 0.47 | 0.86 | 840999 | | 140650 | | | 144343 | | | 164931 | | | 0.39* | | |
| 0.40 | 0.47 | 0.86 | 840999 | | 140650 | | | 144343 | | | 164931 | | | 0.39* | | |
| 0.45 | 0.47 | 0.86 | 840999 | | 140650 | | | 144343 | | | 164931 | | | 0.39* | | |
| 0.50 | 0.47 | 0.86 | 840999 | | 140650 | | | 144343 | | | 164931 | | | 0.39* | | |
| 0.55 | 0.47 | 0.86 | 840614 | | 141035 | | | 144232 | | | 165042 | | | 0.39* | | |
| 0.60 | 0.46 | 0.85 | 836162 | | 145487 | | | 143543 | | | 165731 | | | 0.39* | | |
| 0.65 | 0.46 | 0.84 | 822783 | | 158866 | | | 141090 | | | 168184 | | | 0.38 | | |
| 0.70 | 0.44 | 0.82 | 803695 | | 177954 | | | 135973 | | | 173301 | | | 0.38 | | |
| 0.75 | 0.41 | 0.79 | 772776 | | 208873 | | | 127347 | | | 181927 | | | 0.38 | | |
| 0.80 | 0.37 | 0.74 | 725627 | | 256022 | | | 113557 | | | 195717 | | | 0.37 | | |
| 0.85 | 0.32 | 0.68 | 669390 | | 312259 | | | 99272 | | | 210002 | | | 0.36 | | |
| 0.90 | 0.25 | 0.60 | 584746 | | 396903 | | | 78643 | | | 230631 | | | 0.35 | | |
| 0.95 | 0.20 | 0.48 | 468003 | | 513646 | | | 60602 | | | 248672 | | | 0.28 | | |

**Table S2**

**Sensitivity and specificity of the weighted‑scoring scheme against a reference set of 6599 Pfam domains with PDB/DSSP information.**

Based on the data from Table S1, quality‑score [1], PSIPred [2,3], SEG [4] and GlobPlot [5] obtained their best predictive performance at (TPR‑FPR) of 0.61, 0.50, 0.41 and 0.39 respectively. The latter serves as the predictor‑specific weight variables $w_{predictor}$ in the proposed weighted‑scoring scheme (see equations 1-2) which combines the four predictors' outputs into a singular value $normscore_{weighted}$. As such, the sensitivity and specificity of the weighted‑scoring scheme can also be computed and is provided in the following table.

| Weighted-score (Quality‑score+PSIPred+SEG+GlobPlot): | | | | | | | |
| --- | --- | --- | --- | --- | --- | --- | --- |
| Threshold | FPR | TPR | TP | FN | FP | TN | TPR‑FPR |
| 0.05 | 0.62 | 0.99 | 1067476 | 10481 | 201130 | 124088 | 0.37 |
| 0.10 | 0.62 | 0.99 | 1067465 | 10492 | 201130 | 124088 | 0.37 |
| 0.15 | 0.62 | 0.99 | 1067293 | 10664 | 200668 | 124550 | 0.37 |
| 0.20 | 0.57 | 0.99 | 1062349 | 15608 | 184213 | 141005 | 0.42 |
| 0.25 | 0.51 | 0.98 | 1051531 | 26426 | 165302 | 159916 | 0.47 |
| 0.30 | 0.50 | 0.97 | 1048408 | 29549 | 162002 | 163216 | 0.47 |
| 0.35 | 0.47 | 0.96 | 1038916 | 39041 | 152099 | 173119 | 0.49 |
| 0.40 | 0.39 | 0.93 | 1004806 | 73151 | 125245 | 199973 | 0.54 |
| 0.45 | 0.31 | 0.90 | 966447 | 111510 | 101162 | 224056 | 0.59* |
| 0.50 | 0.28 | 0.87 | 940782 | 137175 | 90462 | 234756 | 0.59* |
| 0.55 | 0.24 | 0.82 | 888936 | 189021 | 78571 | 246647 | 0.58 |
| 0.60 | 0.20 | 0.76 | 815020 | 262937 | 63519 | 261699 | 0.56 |
| 0.65 | 0.15 | 0.68 | 735674 | 342283 | 50205 | 275013 | 0.53 |
| 0.70 | 0.12 | 0.61 | 654631 | 423326 | 39586 | 285632 | 0.49 |
| 0.75 | 0.10 | 0.53 | 575612 | 502345 | 31160 | 294058 | 0.43 |
| 0.80 | 0.08 | 0.47 | 502496 | 575461 | 25303 | 299915 | 0.39 |
| 0.85 | 0.06 | 0.38 | 405194 | 672763 | 20713 | 304505 | 0.32 |
| 0.90 | 0.05 | 0.26 | 284437 | 793520 | 16654 | 308564 | 0.21 |
| 0.95 | 0.04 | 0.16 | 170176 | 907781 | 13112 | 312106 | 0.12 |

**Table S3**

**Full list of dissectHMMER results for the analysis of TIP12_MAIZE**

In all, dissectHMMER found 11 domain hits. For each domain hit, the Pfam accession, domain name, domain length and representative PDB (if any) are given in column 1. Column 2 gives the sequence range (i.e. sequence stretch covered by the domain) and the domain coverage where 1 indicates full coverage while <1 implies partial coverage by the domain model. Column 3 gives the original (or undissected) HMMER2 and HMMER3 E‑values of the sequence‑to‑domain alignments. Column 4 gives the coverage score, $\text{coverage}^{\text{ratio}}$ and $\text{coverage}^{\text{fold-critical E-value}}$ (see equation 5) which is the corrected domain coverage score of the HMMER2/HMMER3 sequence‑to‑domain hit. The expected FPRs (false‑positive rates) for the coverage scores are also provided and they were estimated from the relevant dissectHMMER ROC plots in Figure 6. Column 5 gives the sorted total FPR in ascending order, where the latter is the sum of the two independent FPRs as given in column 3. Column 6 gives the RMSD/%Id and alignment range derived from the structure alignments between 1YMG|A and the representative structures of the domain models. The last column gives the biological function of the representative structures.

| **Domain description** | **Sequence range/**  **Domain coverage** | **Original**  **E‑values**  **[HMMER2/**  **HMMER3]** | **[coverage/**  **FPR]^ratio^**  **[coverage/**  **FPR]^fc E‑value^** | **Total FPR** | **RMSD/**  **%Id/**  **Structural alignment range**  **(1YMG|A:pdb)** | **Function description of representative pdb** |
| --- | --- | --- | --- | --- | --- | --- |
| PF00230.15  MIP  length:296  pdb:1YMG|A | 13-234/  1 | 1.46e‑127/  8.50e‑73 | 1.00/0.00  1.00/0.00 | 0.00 | 1.73/  22.8/  10‑224:  10‑243 | 6‑TM water/glycerol channel of malarial parasite Plasmodium falciparum [6] |
| PF00654.15  Voltage_CLC  length:730  pdb:2HLF|A | 1-236/  1 | 3.89e‑01/  6.52e‑05 | 0.345/0.28  0.550/0.01 | 0.29 | 4.99/  6.6/  78‑228:  254‑383 | 12‑TM chloride channel; 3 Cl^-^ bind sites, each a "pore‑like" trajectory transverse to the membrane plane [7] |
| PF01226.12  Form_Nir_trans  length:366  pdb:4FC4|A | 17-238/  1 | 3.54e‑03/- | 0.325/0.32  0.490/0.03 | 0.35 | 3.51/  9.5/  10‑223:  25‑249 | 6‑TM nitrite anion channel of bacteria for cytoplasmic detoxification [8] |
| PF07331.6  TctB  length:266  pdb:- | 112-243/  1 | 2.96e‑02/- | 0.385/0.21  0.420/0.15 | 0.36 | - | - |
| PF07155.7  ECF-ribofla_trS  ength:196  pdb:4HZU|S | 57-190/  1 | 8.10e‑02/- | 0.235/0.48  0.435/0.13 | 0.61 | 3.52/  6.4/  131‑226:  35:162 | 5‑TM pore that transport riboflavin molecules across the lipid bilayer [9] |
| PF06912.6  DUF1275  length:290  pdb:- | 57-216/  1 | 1.20e‑02/- | 0.225/0.50  0.435/0.13 | 0.63 | - | Postulated to be a membrane protein according to Pfam |
| PF01384.15  PHO4  length:1203  pdb:- | 19-231/  1 | 9.35e‑03/- | 0.150/0.63  0.490/0.03 | 0.66 | - | Phosphate transporter [10] |
| PF09490.5  CbtA  length:333  pdb:- | 44-231/  1 | 6.99e‑02/- | 0.110/0.73  0.470/0.06 | 0.79 | - | Probable cobalt transporter subunit with 5 TM helices according to Pfam |
| PF04211.8  MtrC  length:283  pdb:- | 6-246/  1 | 6.32e‑02/- | 0.075/0.83  0.440/0.11 | 0.94 | - | Membrane associated Na+ translocating N5‑methyltetrahydromethanopterin [11,12] |
| PF02028.12  BCCT  length:722  pdb:2WSW|A | 28-242/  1 | 7.24e‑02/- | 0.010/0.98  0.495/0.03 | 1.01 | 9.73/  3.8/  6‑198:  86‑373 | 12‑TM carnitine/  butyrobetaine antiporter [13] |
| PF13303.1  PTS_EIIC_2  length:464  pdb:- | 19-248/  1 |  | 0.030/0.93  0.435/0.13 | 1.06 | - | Bacteria phosphotransferase according to Pfam |

**Table S4**

**Full list of dissectHMMER results for the analysis of Q9K8K1_BACHD**

In total, dissectHMMER found 16 domain hits for consideration. For each domain hit, the Pfam accession, domain name, domain length and representative PDB (if any) are given in column 1. Column 2 gives the sequence range (i.e. sequence stretch covered by the domain) and the domain coverage where 1 indicates full coverage while <1 implies partial coverage by the domain model. Column 3 gives the original (or undissected) HMMER2 and HMMER3 E‑values of the sequence‑to‑domain alignments. Column 4 gives the coverage score, $\text{coverage}^{\text{ratio}}$ and $\text{coverage}^{\text{fold-critical E-value}}$ (see equation 5) which is the corrected domain coverage score of the HMMER2/HMMER3 sequence‑to‑domain hit. The expected FPRs (false‑positive rates) for the coverage scores are also provided and they were estimated from the relevant dissectHMMER ROC plots in Figure 6. Column 5 gives the sorted total FPR in ascending order, where the latter is the sum of the two independent FPRs as given in column 3. Column 6 gives the RMSD/%Id and alignment range derived from the structure alignments between 2CFP|A and the representative structures of the domain models. The last column gives the biological function of the representative structures.

| **Domain**  **description** | **Sequence range/**  **Domain coverage** | **Original**  **E‑values**  **[HMMER2/**  **HMMER3]** | **[coverage/**  **FPR]^ratio^**  **[coverage/**  **FPR]^fc E‑value^** | **Total FPR** | **RMSD/**  **%Id/**  **Structural alignment range**  **(2CFP|A:pdb)** | **Function description of representative pdb** |
| --- | --- | --- | --- | --- | --- | --- |
| PF05684.7  DUF819  length:400  pdb:- | 10‑388/  1 | 5.30e‑244/  3.50e‑162 | 1.00/0.00  1.00/0.00 | 0.00 | - | Unknown function |
| PF07690.11  MFS_1  length:793  pdb:2CFP|A | 13‑382/  1 | 7.72e‑02/  9.98e‑05 | 0.845/0.00  0.815/0.00 | 0.00 | 0.0/  100/  1‑417:  1‑417 | 12‑TM lactose permease (symporter) of E.coli that facilitates lactose and H+ translocation [14]. |
| PF03706.8  UPF0104  length:626  pdb:- | 108‑388/  1 | 5.15e‑02/  1.07e‑03 | 0.615/0.01  0.630/0.00 | 0.01 | - | Unknown function |
| PF00375.13  SDF  length:618  pdb:3V8G|A | 7‑388/  1 | 8.52e‑02/  9.46e‑05 | 0.450/0.09  0.740/0.00 | 0.09 | 6.2/  3.1/  1‑156:  218‑399 | 7‑TM glutamate transporter homolog from Pyrococcus horikoshii [15]. |
| PF00999.16  Na_H_Exchanger  length:593  pdb:4BWZ|A | 10‑387/  1 | 8.25e‑03/- | 0.415/0.15  0.490/0.03 | 0.18 | 4.99/  6.7/  33‑180:  75‑238 | 12‑TM sodium/proton (Na(+)/H(+)) antiporters [16]. |
| PF13347.1  MFS_2  length:847  pdb:4LDS|A | 36‑382/  1 | 8.31e‑02/- | 0.415/0.15  0.490/0.03 | 0.18 | 3.62/  9.4/  11‑401:  7‑423 | 12‑TM glucose/H(+) symporter of Staphylococcus epidermidis [17] |
| PF07155.7  ECF-ribofla_trS  length:196  pdb:4HZU|S | 251‑387/  1 | 2.18e‑03/- | 0.375/0.23  0.485/0.04 | 0.27 | 9.12/  4.6/  52‑201:  20‑163 | 5‑TM pore that transport riboflavin molecules across the lipid bilayer [9] |
| PF03547.13  Mem_trans  length:746  pdb:- | 29‑379 | 3.11e‑03/- | 0.375/0.23  0.485/0.04 | 0.27 | - | Unknown function |
| PF07884.9  VKOR  length:174  pdb:3KP9|A | 250‑379/  1 | 7.51e‑04/- | 0.395/0.18  0.435/0.13 | 0.31 | 8.2/  2.6/  1‑205:  45‑239 | Vitamin K epoxide reductase (VKOR) with a catalytic core of 4‑TM helix bundle [18] |
| PF00083.19  Sugar_tr  length:605  pdb:4GC0|A | 6‑385/  1 | 5.20e‑02/- | 0.225/0.50  0.485/0.04 | 0.54 | 4.79/  6.5/  4‑388:  5‑428 | 12‑TM D‑xylose or d‑glucose transporter [19] |
| PF00115.15  COX1  length:591  pdb:1V55|A | 22‑381/  1 | 7.88e‑02/- | 0.120/0.70  0.475/0.06 | 0.76 | 7.4/  1.7/  9‑417:  9‑501 | 12‑TM mitochondrial cytochrome c oxidase that contains two proton pumps and a water channel [20]. |
| PF09847.4  DUF2074  length:504  pdb:- | 1‑388/  1 | 8.15e‑02/- | 0.100/0.75  0.420/0.15 | 0.90 | - | Unknown function |
| PF07556.6  DUF1538  length:262  pdb:- | 222‑377/  1 | 1.00e‑01/- | 0.070/0.83  0.450/0.10 | 0.93 | - | Unknown function |
| PF03611.9  EIIC-GAT  length:642  pdb:- | 7‑379/  1 | 1.28e‑03/- | 0.035/0.90  0.485/0.04 | 0.94 | - | 10‑TM 3‑keto‑L‑gulonate sugar‑specific permease [21]. |
| PF03169.10  OPT  length:1010  pdb:- | 3‑388/  1 | 3.73e‑02/- | 0.050/0.88  0.450/0.10 | 0.98 | - | 12‑14 TM oligopeptide transporter protein [22]. |
| PF02028.12  BCCT  length:722  pdb:2WSW|A | 7‑330/  1 | 1.18e‑02/- | 0.005/1.00  0.500/0.01 | 1.01 | 7.73/  3.1/  8‑212:  48‑404 | 12‑TM carnitine/  butyrobetaine antiporter [13] |

**References**

1. Thompson JD, Gibson TJ, Plewniak F, Jeanmougin F, Higgins DG: **The CLUSTAL_X windows interface: flexible strategies for multiple sequence alignment aided by quality analysis tools.** *Nucleic Acids Res* 1997, **25:**4876-4882.

2. Buchan DW, Minneci F, Nugent TC, Bryson K, Jones DT: **Scalable web services for the PSIPRED Protein Analysis Workbench.** *Nucleic Acids Res* 2013, **41:**W349-W357.

3. Jones DT: **Protein secondary structure prediction based on position-specific scoring matrices.** *J Mol Biol* 1999, **292:**195-202.

4. Wootton JC, Federhen S: **Analysis of compositionally biased regions in sequence databases.** *Methods Enzymol* 1996, **266:**554-571.

5. Linding R, Russell RB, Neduva V, Gibson TJ: **GlobPlot: Exploring protein sequences for globularity and disorder.** *Nucleic Acids Res* 2003, **31:**3701-3708.

6. Newby ZE, O'Connell J, III, Robles-Colmenares Y, Khademi S, Miercke LJ, Stroud RM: **Crystal structure of the aquaglyceroporin PfAQP from the malarial parasite Plasmodium falciparum.** *Nat Struct Mol Biol* 2008, **15:**619-625.

7. Accardi A, Lobet S, Williams C, Miller C, Dutzler R: **Synergism between halide binding and proton transport in a CLC-type exchanger.** *J Mol Biol* 2006, **362:**691-699.

8. Lu W, Schwarzer NJ, Du J, Gerbig-Smentek E, Andrade SL, Einsle O: **Structural and functional characterization of the nitrite channel NirC from Salmonella typhimurium.** *Proc Natl Acad Sci U S A* 2012, **109:**18395-18400.

9. Zhang P, Wang J, Shi Y: **Structure and mechanism of the S component of a bacterial ECF transporter.** *Nature* 2010, **468:**717-720.

10. Versaw WK, Metzenberg RL: **Repressible cation-phosphate symporters in Neurospora crassa.** *Proc Natl Acad Sci U S A* 1995, **92:**3884-3887.

11. Lienard T, Becher B, Marschall M, Bowien S, Gottschalk G: **Sodium ion translocation by N5-methyltetrahydromethanopterin: coenzyme M methyltransferase from Methanosarcina mazei Go1 reconstituted in ether lipid liposomes.** *Eur J Biochem* 1996, **239:**857-864.

12. Lienard T, Gottschalk G: **Cloning, sequencing and expression of the genes encoding the sodium translocating N5-methyltetrahydromethanopterin : coenzyme M methyltransferase of the methylotrophic archaeon Methanosarcina mazei Go1.** *FEBS Lett* 1998, **425:**204-208.

13. Schulze S, Koster S, Geldmacher U, Terwisscha van Scheltinga AC, Kuhlbrandt W: **Structural basis of Na(+)-independent and cooperative substrate/product antiport in CaiT.** *Nature* 2010, **467:**233-236.

14. Mirza O, Guan L, Verner G, Iwata S, Kaback HR: **Structural evidence for induced fit and a mechanism for sugar/H+ symport in LacY.** *EMBO J* 2006, **25:**1177-1183.

15. Verdon G, Boudker O: **Crystal structure of an asymmetric trimer of a bacterial glutamate transporter homolog.** *Nat Struct Mol Biol* 2012, **19:**355-357.

16. Lee C, Kang HJ, von BC, Newstead S, Uzdavinys P, Dotson DL, Iwata S, Beckstein O, Cameron AD, Drew D: **A two-domain elevator mechanism for sodium/proton antiport.** *Nature* 2013, **501:**573-577.

17. Iancu CV, Zamoon J, Woo SB, Aleshin A, Choe JY: **Crystal structure of a glucose/H+ symporter and its mechanism of action.** *Proc Natl Acad Sci U S A* 2013, **110:**17862-17867.

18. Li W, Schulman S, Dutton RJ, Boyd D, Beckwith J, Rapoport TA: **Structure of a bacterial homologue of vitamin K epoxide reductase.** *Nature* 2010, **463:**507-512.

19. Sun L, Zeng X, Yan C, Sun X, Gong X, Rao Y, Yan N: **Crystal structure of a bacterial homologue of glucose transporters GLUT1-4.** *Nature* 2012, **490:**361-366.

20. Tsukihara T, Shimokata K, Katayama Y, Shimada H, Muramoto K, Aoyama H, Mochizuki M, Shinzawa-Itoh K, Yamashita E, Yao M et al.: **The low-spin heme of cytochrome c oxidase as the driving element of the proton-pumping process.** *Proc Natl Acad Sci U S A* 2003, **100:**15304-15309.

21. Yew WS, Gerlt JA: **Utilization of L-ascorbate by Escherichia coli K-12: assignments of functions to products of the yjf-sga and yia-sgb operons.** *J Bacteriol* 2002, **184:**302-306.

22. Lubkowitz MA, Barnes D, Breslav M, Burchfield A, Naider F, Becker JM: **Schizosaccharomyces pombe isp4 encodes a transporter representing a novel family of oligopeptide transporters.** *Mol Microbiol* 1998, **28:**729-741.
