# Supplementary material for: dissectHMMER: a HMMER-based score dissection framework that statistically evaluates fold-critical sequence segments for domain fold similarity
Source: Biol Direct. 2015 Aug 1;10:39. doi: 10.1186/s13062-015-0068-3 (PMC4521371; doi:10.1186/s13062-015-0068-3)
Supplement: Additional file 6: — The web output of dissectHMMER search results for the protein sequence HEM1_METKA. (ZIP 9 kb) [file 13062_2015_68_MOESM6_ESM.zip › 5864776041655568_add6.html]

  
  

dissectHMMER


# dissectHMMER results

---

**Overview of Sequence Annotation**  

  
Domain(s) to sequence segment[146,323]  
Domain name?                    &nbspPDB?    &nbspLength?  &nbspSeq range?  &nbspScore[FC/R]?  &nbspFPR[FC/R]?  &nbspScore[FC]?  &nbspFPR[FC]?  &nbspDomaincoverage?   **Total FPR?**   **Hits'classification?**     
PF00106.20\_adh\_short\_seed    ?  &nbsp1IPE|A  &nbsp225      &nbsp168,305     &nbsp0.64          &nbsp0.00        &nbsp0.645       &nbsp0.00      &nbsp1                 **0**   **TP,TP**  
PF01488.15\_Shikimate\_DH\_seed ?  &nbsp1NVT|A  &nbsp170      &nbsp156,292     &nbsp0.995         &nbsp0.00        &nbsp0.995       &nbsp0.00      &nbsp1                 **0**   **TP,TP**  
PF02826.14\_2-Hacid\_dh\_C\_seed ?  &nbsp3ORQ|A  &nbsp260      &nbsp127,269     &nbsp0.645         &nbsp0.00        &nbsp0.67        &nbsp0.00      &nbsp1                 **0**   **TP,TP**  
PF02882.14\_THF\_DHG\_CYH\_C\_seed?  &nbsp1A4I|A  &nbsp205      &nbsp131,258     &nbsp0.78          &nbsp0.00        &nbsp0.795       &nbsp0.00      &nbsp1                 **0**   **FN,FN**  
PF03446.10\_NAD\_binding\_2\_seed?  &nbsp1PGQ|A  &nbsp235      &nbsp167,321     &nbsp0.7           &nbsp0.00        &nbsp0.71        &nbsp0.00      &nbsp1                 **0**   **FN,FN**  
PF03807.12\_F420\_oxidored\_seed?  &nbsp2VNS|A  &nbsp123      &nbsp169,268     &nbsp0.975         &nbsp0.00        &nbsp0.98        &nbsp0.00      &nbsp1                 **0**   **TP,TP**  
PF08659.5\_KR\_seed            ?  &nbsp2VZ9|A  &nbsp257      &nbsp169,281     &nbsp0.57          &nbsp0.01        &nbsp0.725       &nbsp0.00      &nbsp1                 **0.01**   **FN,FN**  
PF13241.1\_NAD\_binding\_7\_seed ?  &nbsp1PJQ|A  &nbsp379      &nbsp162,275     &nbsp0.535         &nbsp0.01        &nbsp0.535       &nbsp0.01      &nbsp1                 **0.02**   **TP,FN**  
PF13460.1\_NAD\_binding\_10\_seed?  &nbsp1HDO|A  &nbsp362      &nbsp170,344     &nbsp0.495         &nbsp0.03        &nbsp0.49        &nbsp0.03      &nbsp1                 **0.06**   **TP**  
PF01408.17\_GFO\_IDH\_MocA\_seed ?  &nbsp2IXB|A  &nbsp188      &nbsp168,265     &nbsp0.445         &nbsp0.11        &nbsp0.45        &nbsp0.10      &nbsp1                 **0.21**   **TP**  
PF02254.13\_TrkA\_N\_seed       ?  &nbsp1LSS|A  &nbsp195      &nbsp170,285     &nbsp0.4           &nbsp0.18        &nbsp0.42        &nbsp0.15      &nbsp1                 **0.33**   **TP**  
PF03949.10\_Malic\_M\_seed      ?  &nbsp1DO8|A  &nbsp324      &nbsp146,323     &nbsp0.14          &nbsp0.66        &nbsp0.485       &nbsp0.04      &nbsp1                 **0.7**   **TP**  
PF07991.7\_IlvN\_seed          ?  &nbsp1YVE|A  &nbsp177      &nbsp165,233     &nbsp0.205         &nbsp0.53        &nbsp0.205       &nbsp0.54      &nbsp0.42              **1.07**   **TP**  
  

  
Domain(s) to sequence segment[7,142]  
Domain name?              &nbspPDB?    &nbspLength?  &nbspSeq range?  &nbspScore[FC/R]?  &nbspFPR[FC/R]?  &nbspScore[FC]?  &nbspFPR[FC]?  &nbspDomaincoverage?   **Total FPR?**   **Hits'classification?**     
PF05201.10\_GlutR\_N\_seed?  &nbsp1GPJ|A  &nbsp222      &nbsp7,142       &nbsp1             &nbsp0.00        &nbsp1           &nbsp0.00      &nbsp1                 **0**   **TP,TP**  
  

  
Domain(s) to sequence segment[305,403]  
Domain name?                  &nbspPDB?    &nbspLength?  &nbspSeq range?  &nbspScore[FC/R]?  &nbspFPR[FC/R]?  &nbspScore[FC]?  &nbspFPR[FC]?  &nbspDomaincoverage?   **Total FPR?**   **Hits'classification?**     
PF00745.15\_GlutR\_dimer\_seed?  &nbsp1GPJ|A  &nbsp132      &nbsp305,403     &nbsp0.97          &nbsp0.00        &nbsp0.975       &nbsp0.00      &nbsp1                 **0**   **TP,TP**

---

**Alignments**  
  
**PF00106.20\_adh\_short\_seed:**  

**E:1.10e-02[Original]  &nbspE:3.62e-07[Fold-critical]  &nbspE:2.07e-02[Remnant]  &nbspRatio[FC/R]:1.75e-05  &nbspClassification:TP**

STRUCT   SEEEEET?SSHHHHHHHHHHHHT-T?SEEEEEESS-------------GGGSTTHHHH-----HHHHH---HTT--?EEEEEE-??---TT?H-HHHHHHHHT??---TTS?EEEEEE????---??-S???TTT?-----?HHHHH--HHHHTT----HHHHHHHHHHHTT----S?------?SEEEEEEEHHHHTT?TT?HHHHHHHHHHHHHHHHHHHTT?  &nbspPDB/DSSP  
MODEL    GTVLITGGTGGLGLAVARWLVAEHGARHLVLVSRRGLPDRSKWARRSLGPDAPGAAELEEKRRVAELEALEALGPEAEVTVAAQCDACVVADRLDALAALLAAIPKAAAEGPLTGVVHAAGVAFKLDGGDGVLESLESLTSTPERLDDGRVLAPKVNGVVDGAWHLHELTRDLMIKLDKGKDGELDAFVLFSSAAGVLGSPGQANYAAANAFLDALAEHRRAEGL  &nbspHMMER2[PF00106.20\_adh\_short\_seed]  
QUERY    KTVLVVG-AGEMGKTVAKSLVDR-GVRAVLVANRT--YERAVELARDLGGEAVRFDEL-----VDHLA--RSDV-VVSATAAP-HP---VIHV-DDVREALRKRD---RRSPILIID----I---AN--PRDVEEG------VENIE-----DVE----VRTIDDLRVIARE---NLE----------------------------------------RRRKEIP   Q9UXR8|Glutamyl-tRNA\_reductase|HEM1\_METKA[168 305]  
  

**E:5.53e-05[Original]  &nbspE:3.41e-11[Fold-critical]  &nbspE:1.42e+04[Remnant]  &nbspRatio[FC/R]:2.40e-15  &nbspClassification:TP**

STRUCT   SEEEEET?SSHHHHHHHHHHHHT-T?SEEEEEESS-------------GGGSTTHHHH-----HHHHH---HTT--?EEEEEE-??---TT?H-HHHHHHHHT??---TTS?EEEEEE????---??-S???TTT?-----?HHHHH--HHHHTT----HHHHHHHHHHHTT----S?------?SEEEEEEEHHHHTT?TT?HHHHHHHHHHHHHHHHHHHTT?  &nbspPDB/DSSP  
MODEL    GTVLITGGLGGLGLALARWLVEEHGARHLVLVSRRALPERSKWAEKSLGEDAPGAAELEEKLAIAELExxxxxxxxxxxxxxxxxxxxxxxxxxxxxxxxxxxxxxxxxxxxxxxxxxxxxxxxxxxxxxxxxxxxxxxxxxxxxxxxxxxxxxxxxxxxxxxxxxxxxxxxxxxxxxxxxxxxxxxxxxxxxxxxxxxxxxxxxxxxxxxxxxxxxxxxxxxxx  &nbspHMMER3[PF00106.20\_adh\_short\_seed]  
QUERY    KTVLVVG-AGEMGKTVAKSLVDR-GVRAVLVANRT--YERAVELARDLGGEAVRFDEL-----VDHLAxxxxxxxxxxxxxxxxxxxxxxxxxxxxxxxxxxxxxxxxxxxxxxxxxxxxxxxxxxxxxxxxxxxxxxxxxxxxxxxxxxxxxxxxxxxxxxxxxxxxxxxxxxxxxxxxxxxxxxxxxxxxxxxxxxxxxxxxxxxxxxxxxxxxxxxxxxxxx   Q9UXR8|Glutamyl-tRNA\_reductase|HEM1\_METKA[168 226]  
  
  
**PF01488.15\_Shikimate\_DH\_seed:**  

**E:1.90e-67[Original]  &nbspE:5.07e-22[Fold-critical]  &nbspE:6.54e-10[Remnant]  &nbspRatio[FC/R]:7.75e-13  &nbspClassification:TP**

STRUCT   HHHHHHHS---??T-T?EEEEES??HHHHHHHHHHHHH-??SEEEEE?SSHHHHHHHHHHH-----T?E---E??GGGHH---HHHHT?SEEE-E??SSSS?------------?B?HHHHHHHH---HH?SS???E-EEEE??SS?SB?TTGGGSTTEEEEEHHHHHHH  &nbspPDB/DSSP  
MODEL    ELAKKIFGKSEDLKEGKKVLLIGAGEMAELVAKHLLAKLGAKKITIANRTYERAKELAEEFKERRNGGVEIEALPLDELESIEELLAEADIVIASATSAPEPPEEDIRARELVPIITKEMVERALKAFKKRKRRKPLRLFVDIAVPRDIEPEVGELEGVYVYTVDDLKEV  &nbspHMMER2[PF01488.15\_Shikimate\_DH\_seed]  
QUERY    ELAERELG---SLH-DKTVLVVGAGEMGKTVAKSLVDR-GVRAVLVANRTYERAVELARDL-----GGE---AVRFDELV---DHLARSDVVV-SATAAPHP------------VIHVDDVREAL---RKRDRRSPI-LIIDIANPRDVEEGVENIEDVEVRTIDDLRVI   Q9UXR8|Glutamyl-tRNA\_reductase|HEM1\_METKA[156 292]  
  

**E:3.21e-40[Original]  &nbspE:7.49e-37[Fold-critical]  &nbspE:1.25e-06[Remnant]  &nbspRatio[FC/R]:5.99e-31  &nbspClassification:TP**

STRUCT   HHHHHHHS---??T-T?EEEEES??HHHHHHHHHHHHH-??SEEEEE?SSHHHHHHHHHHH-----T?E---E??GGGHH---HHHHT?SEEE-E??SSSS?------------?B?HHHHHHHH---HH?SS???E-EEEE??SS?SB?TTGGGSTTEEEEEHHHHHHH  &nbspPDB/DSSP  
MODEL    ELAKKKFEKSESLKEGKKVLVIGAGEMARLAAKHLLSKLGVKKIVIANRTAEKAKELAEELKERRNGGEEVEALPLEELEAIAELLAEADVVIASATSAPTPTEEEIRARELVRIIEKEAVEEAKKASKKRKAEKPLGLLVDIAVPRDIEPEVAELEDVRVYNVDDLKEx  &nbspHMMER3[PF01488.15\_Shikimate\_DH\_seed]  
QUERY    ELAERELG---SLH-DKTVLVVGAGEMGKTVAKSLVDR-GVRAVLVANRTYERAVELARDL-----GGE---AVRFDELV---DHLARSDVVV-SATAAPHP------------VIHVDDVREAL---RKRDRRSPI-LIIDIANPRDVEEGVENIEDVEVRTIDDLRVx   Q9UXR8|Glutamyl-tRNA\_reductase|HEM1\_METKA[156 291]  
  
  
**PF02826.14\_2-Hacid\_dh\_C\_seed:**  

**E:8.79e-03[Original]  &nbspE:7.17e-08[Fold-critical]  &nbspE:3.51e-04[Remnant]  &nbspRatio[FC/R]:2.04e-04  &nbspClassification:TP**

STRUCT   HHHHHHHHTTH---HHHHHHH----TTT----??--------?TT---T??B?----??TT?EEEEE??SHHHHHHHHHHH-HTT?-EEEEE?SS----??----HHH---HHHTT?-EE?-?HHHH---HHH?SEEEE???-?STTTTT?B?HHHHHHS?TT?-----EEEE?S?GGGB?HHHHHHHHHHTSSSEEEES??SSS-----------S??----TT--------?---GG----GG--?TT-----EEE?SS?T  &nbspPDB/DSSP  
MODEL    LALLLALARRIINGPEADRQVKRWKRAGRRDDKWESSGLGAWRPGCYFALLGRAGLRELSGKTVGIIGLGRIGRAVARRAKHAFGMKKVIAYDRYWSSDPKKKKEPEEAEEAEALGATRYVDSLDEL---LAESDVVSLHLPYLTPETRHLINAERLAKMKPGAPLGEPILINTARGGLVDEDALIDALKSGRIAGAALDVFEPESGYFFKDRKTKPLPAEVVADAKAFQKKLHTLKPLLKLLLESSLPNTIADKVILTPHIA  &nbspHMMER2[PF02826.14\_2-Hacid\_dh\_C\_seed]  
QUERY    FRRAINLGKRA---REETRIS-----EG----AVSIG-SAAVELA---ERELG----SLHDKTVLVVGAGEMGKTVAKSLV-DRGVRAVLVANRT----YE----RAV-ELARDLGG-EAV-RFDELVDHLARSDVVVSATA------------------APH--------------PVIHVDDVREALRK-RDR----------------------------RS------------PILIIDIA--NPR-------------   Q9UXR8|Glutamyl-tRNA\_reductase|HEM1\_METKA[127 269]  
  

**E:5.29e-09[Original]  &nbspE:3.25e-10[Fold-critical]  &nbspE:5.77e+00[Remnant]  &nbspRatio[FC/R]:5.63e-11  &nbspClassification:TP**

STRUCT   HHHHHHHHTTH---HHHHHHH----TTT----??--------?TT---T??B?----??TT?EEEEE??SHHHHHHHHHHH-HTT?-EEEEE?SS----??----HHH---HHHTT?-EE?-?HHHHHHH?SEEEE???-?STTTTT?B?HHHHHHS?TT?-----EEEE?S?GGGB?HHHHHHHHHHTSSSEEEES??SSS-----------S??----TT--------?---GG----GG--?TT-----EEE?SS?T  &nbspPDB/DSSP  
MODEL    xxxxxxxxxxxxxxxxxxxxxxxxxxxxxxxxxxxxxxxxxxxxxxxxALLGKAGLEELSGKTVGIIGLGRIGQAVAKRLKKAFGMKKVLAYDRSWSSDPKKKKEPEEAEEEEELGAERYVKSLDELLAESDIVSLHLxxxxxxxxxxxxxxxxxxxxxxxxxxxxxxxxxxxxxxxxxxxxxxxxxxxxxxxxxxxxxxxxxxxxxxxxxxxxxxxxxxxxxxxxxxxxxxxxxxxxxxxxxxxxxxxxxxxxxxxxxx  &nbspHMMER3[PF02826.14\_2-Hacid\_dh\_C\_seed]  
QUERY    xxxxxxxxxxxxxxxxxxxxxxxxxxxxxxxxxxxxxxxxxxxxxxxxELAER-ELGSLHDKTVLVVGAGEMGKTVAKSLV-DRGVRAVLVANRT----YE--RAVELARDLGGEAV-RFD-ELVDHLARSDVVVSATxxxxxxxxxxxxxxxxxxxxxxxxxxxxxxxxxxxxxxxxxxxxxxxxxxxxxxxxxxxxxxxxxxxxxxxxxxxxxxxxxxxxxxxxxxxxxxxxxxxxxxxxxxxxxxxxxxxxxxxxxx   Q9UXR8|Glutamyl-tRNA\_reductase|HEM1\_METKA[156 235]  
  
  
**PF02882.14\_THF\_DHG\_CYH\_C\_seed:**  

**E:5.34e-01[Original]  &nbspE:1.45e-09[Fold-critical]  &nbspE:7.73e-06[Remnant]  &nbspRatio[FC/R]:1.88e-04  &nbspClassification:FN**

STRUCT   SHHHHHHHHTT--??----SS??HHHHHHHHHHHH-TT??---?TT?EEEEE???TTTHHHHHHHHHT---------T--------T?EEEEE?S-----------------S?---S?HHHHHHH?SEEEE?S??TT?B?TTTS---?TT?EEEE???EE?----------TTS?-EE?SB?HHHHHHH-?SEE??SSSSSHHHHHHHHHHHHHHHHHHTT  &nbspPDB/DSSP  
MODEL    HPLNVGRLALGDGEPGDIDGFVPCTPAGIMELLKRAYGIDANFLAGKNAVVIGRSNIVGKPLALLLLNKGSTCGPAQAGPYDTKGGNATVTVCHS-----------------KTPPQKDLAEITRQADILVVAVGKPNLVKADWVANTKPGAVVIDVGINRVPDEDPEASKKGGGKILVGDVDFEEVKEKPASAITPVPGGVGPMTVAMLLKNTLEAAKRQL  &nbspHMMER2[PF02882.14\_THF\_DHG\_CYH\_C\_seed]  
QUERY    TRISEGAVSIG--SA-------------AVELAER-ELGS---LHDKTVLVVGAGE-MGKTVAKSLVD---------R------GVRA-VLVANRTYERAVELARDLGGEAVRF---DELVDHLARSDVVVSATAAPHP-----V-------------IHVD------------------DVR-EALRKR----------------------------DRRS   Q9UXR8|Glutamyl-tRNA\_reductase|HEM1\_METKA[141 258]  
  

**E:8.87e-03[Original]  &nbspE:2.45e-09[Fold-critical]  &nbspE:1.48e+04[Remnant]  &nbspRatio[FC/R]:1.66e-13  &nbspClassification:FN**

STRUCT   SHHHHHHHHTT--??----SS??HHHHHHHHHHHH-TT??---?TT?EEEEE???TTTHHHHHHHHHT---------T--------T?EEEEE?SS?------S?HHHHHHH?SEEEE?S??TT?B?TTTS---?TT?EEEE???EE?----------TTS?-EE?SB?HHHHHHH-?SEE??SSSSSHHHHHHHHHHHHHHHHHHTT  &nbspPDB/DSSP  
MODEL    xxVNLGRLVLGEEEEGDLDALLPCTPKGIVELLKRAYGIEANLLEGKKVVVVGRSNIVGKPLALLLLNKDSTAGPAKAAKKATAAVNATVTICHSKT---PPKKDLAEITREADIVVVAVGKPELVxxxxxxxxxxxxxxxxxxxxxxxxxxxxxxxxxxxxxxxxxxxxxxxxxxxxxxxxxxxxxxxxxxxxxxxxxxxxxxxxxx  &nbspHMMER3[PF02882.14\_THF\_DHG\_CYH\_C\_seed]  
QUERY    xxINLGKRARE--ET-RISEGAVSIGSAAVELAER-ELGS---LHDKTVLVVGAGE-MGKTVAKSLVDRGVRAVLVA-----NRTYERAVELARDLGGEAVRFDELVDHLARSDVVVSATAAPHPVxxxxxxxxxxxxxxxxxxxxxxxxxxxxxxxxxxxxxxxxxxxxxxxxxxxxxxxxxxxxxxxxxxxxxxxxxxxxxxxxxx   Q9UXR8|Glutamyl-tRNA\_reductase|HEM1\_METKA[131 241]  
  
  
**PF03446.10\_NAD\_binding\_2\_seed:**  

**E:3.00e-01[Original]  &nbspE:4.04e-07[Fold-critical]  &nbspE:6.43e-03[Remnant]  &nbspRatio[FC/R]:6.28e-05  &nbspClassification:FN**

STRUCT   ?BSEEEE??SHHHHHHHHHH---H-HTT??-EEEE?-SS--H-----HHHHHHH-H---HHT-----TSSEEE?SSHH-HHHHTB?---SS?EEEE?S?TTHHHHHHHHH--------HT--TT?--------?TT--?-----EEEE?S???HHHHHHHHHH-HHHHT--?EEEEEEEESHHHHHHH??-EEEEE---E?HH-HHHHHHH-HHHHHS?EET---TEESB?-------??BS  &nbspPDB/DSSP  
MODEL    MAKIGFIGLGVMGSPMALNLYNKAGKAGYT-VTVYNIRTLEPPGVEEEKTEELVAEFAGGAKEKPAGGKNAVGAASPAVEFVASLEGEPKPDVVITMVPAGAAVDAVILGPEDGSGGGLLEGPGLKKCATDISEPGHRD-----IIIDGSTSDPEDTRRRAKELLAEKGPDIHFLDAPVSGGEEGARNGTLSIMVGR-EGDEEKAFERVKPYILEAIGAKVDKNDGEPCVTAPDRGGHYIGP  &nbspHMMER2[PF03446.10\_NAD\_binding\_2\_seed]  
QUERY    DKTVLVVGAGEMGKTVAKSL---V-DRGVRAVLVAN-RT--Y-----ERAVELARD--LGGE--------AVRFDELV-DHLARS------DVVVSATAAPHPVIHVDD---------VR--EAL--------RKR--DRRSPILIIDIAN--PRDVEEGVEN-IEDVE--VRTID------DLRVIARE------NLERRRK-EIPKVEK-LIEEELS---------TVE-------E--E   Q9UXR8|Glutamyl-tRNA\_reductase|HEM1\_METKA[167 321]  
  

**E:1.27e-03[Original]  &nbspE:3.07e-09[Fold-critical]  &nbspE:1.48e+04[Remnant]  &nbspRatio[FC/R]:2.07e-13  &nbspClassification:FN**

STRUCT   ?BSEEEE??SHHHHHHHHHH---H-HTT?-?EEEE?-SS--H-----HHHHHHH-H---HHT-----TSSEEE?SSHH-HHHHTB?---SS?EEEE?S?TTHHHHHHHHH--------HT--TT?--------?TT--?EEEE?S???HHHHHHHHHH-HHHHT--?EEEEEEEESHHHHHHH??-EEEEE--E?HH-HHHHHHH-HHHHHS?EET---TEESB?-------??BS  &nbspPDB/DSSP  
MODEL    xxKIGFIGLGVMGSPMALNLYNKLGKAGY-EVAVYNLRTLEPPGVEEEKVEELVAEFAAGAKASPANKKKAKAAESIELEFVASLESEPKPDVVITMVPAGAAVDEVxxxxxxxxxxxxxxxxxxxxxxxxxxxxxxxxxxxxxxxxxxxxxxxxxxxxxxxxxxxxxxxxxxxxxxxxxxxxxxxxxxxxxxxxxxxxxxxxxxxxxxxxxxxxxxxxxxxxxxxxxxxxxxxxx  &nbspHMMER3[PF03446.10\_NAD\_binding\_2\_seed]  
QUERY    xxTVLVVGAGEMGKTVAKSL---V-DRGVRAVLVAN-RT--Y-----ERAVELARD--LGG--------EAVRFDELV-DHLARS------DVVVSATAAPHPVIHVxxxxxxxxxxxxxxxxxxxxxxxxxxxxxxxxxxxxxxxxxxxxxxxxxxxxxxxxxxxxxxxxxxxxxxxxxxxxxxxxxxxxxxxxxxxxxxxxxxxxxxxxxxxxxxxxxxxxxxxxxxxxxxxxx   Q9UXR8|Glutamyl-tRNA\_reductase|HEM1\_METKA[169 244]  
  
  
**PF03807.12\_F420\_oxidored\_seed:**  

**E:8.73e-06[Original]  &nbspE:2.58e-07[Fold-critical]  &nbspE:5.19e-03[Remnant]  &nbspRatio[FC/R]:4.97e-05  &nbspClassification:TP**

STRUCT   EEEEETTTSHHHHHHHHHHHHTT-------?EEEEE-ESSHHHHHHHH-HHHHHH-------H?S??EEEEEHHHHHH---H?SEEEE?-------S?GGGHHHHHHHT-------HHHHTTSEEEE????  &nbspPDB/DSSP  
MODEL    KIGIIGGAGNMGEALARGLAAAGVTPSLPPHEVIIANSRNPEKAAALALEEYGEILAECGDEGSDVKATAVSNEEAAE-LIEADVVFLA-------VPPQAVPEVLSELGNATAAAADLLKGKLVISITNG  &nbspHMMER2[PF03807.12\_F420\_oxidored\_seed]  
QUERY    TVLVV-GAGEMGKTVAKSLVDRGV------RAVLVA-NRTYERAVELA-RDLG--------------GEAVRFDELVDHLARSDVVVSATAAPHPVIHVDDVREALRKR--------DRRSPILIIDIANP   Q9UXR8|Glutamyl-tRNA\_reductase|HEM1\_METKA[169 268]  
  

**E:2.93e-07[Original]  &nbspE:3.28e-11[Fold-critical]  &nbspE:1.14e+03[Remnant]  &nbspRatio[FC/R]:2.88e-14  &nbspClassification:TP**

STRUCT   EEEEETTTSHHHHHHHHHHHHTT-------?EEEEE-ESSHHHHHHHH-HHHHHH-------H?S??EEEEEHHHHHH--H?SEEEE?S----?GGGHHHHHHHT-------HHHHTTSEEEE????  &nbspPDB/DSSP  
MODEL    xxxIIGGTGNIGEALARGLAAAGATPELPKHEVIIANSRSPEKAEELALEELGEILENCGDEKSDVKVTGVSNEEAAELIEADVVILAV----KPEDVEEVLSELANATAAAKEALKGKLVISILAG  &nbspHMMER3[PF03807.12\_F420\_oxidored\_seed]  
QUERY    xxxVV-GAGEMGKTVAKSLVDRGV------RAVLVA-NRTYERAVELA-RDLG---------GEAVR--FDELVDHLA--RSDVVVSATAAPHPVIHVDDVREAL-----RKRDRRSPILIIDIANP   Q9UXR8|Glutamyl-tRNA\_reductase|HEM1\_METKA[172 268]  
  
  
**PF08659.5\_KR\_seed:**  

**E:4.86e+00[Original]  &nbspE:1.10e-04[Fold-critical]  &nbspE:4.77e-05[Remnant]  &nbspRatio[FC/R]:2.31e+00  &nbspClassification:FN**

STRUCT   SEEEEETTTSH---HHHHHHHHHH-H----TT-?SEEEEEESS-G-------------GGSTTHH-----H------------HH----HHHHH-T-T?----EEEEEE??--TT?HHHHHHH---HHTS?SSS----?EEEEEE??????S??-?TTT??HHHHHHHHHTTHHHHHHHHHHHTT-------S??SEEEEEEEGGG-TT??T--T?HHHHHHHHHHHHHHHHHHHTT??------?EEEEE??BSSS  &nbspPDB/DSSP  
MODEL    GTYLVTGGLGGTGTLGLELARWLANEGIGGRGPARHLVLLSRSPGLPPRRAWEWLEASAPDPEAALASRAAESERRETRARIRLLHPAFAELEADRYGALDIGEVTVVACDEMVADRDAVRALFDLLAEIRADGEELPPLRGVIHAAGVLRRDAILLANMTAEDFARVLAPKVTGAWNLHEATRDADPLLLGRPLDFFVLFSSIAGKVLGSAKFGQANYAAANAFLDALAHYRRAQGLPGRRRGRALSINWGPWADG  &nbspHMMER2[PF08659.5\_KR\_seed]  
QUERY    -TVLVVG-AGE---MGKTVAKSLV-D----RG-VRAVLVANRT-------------------YER------------------AV----ELARD-L-GG----EAVRFD--ELVDHLARSDVV---VSATAAPHP-------VIHVDDVRE----ALRKRDRRSPI---------------LIID------------------IAN-----P------------------RDVEEGVEN--------------IEDV   Q9UXR8|Glutamyl-tRNA\_reductase|HEM1\_METKA[169 281]  
  

**E:6.51e-03[Original]  &nbspE:1.70e-11[Fold-critical]  &nbspE:1.48e+04[Remnant]  &nbspRatio[FC/R]:1.15e-15  &nbspClassification:FN**

STRUCT   SEEEEETTTSH---HHHHHHHHHH-H----TT-?SEEEEEESS-G-------------GGSTTHH-----H------------HH----HHHHH-T-T?----EEEEEE??--TT?HHHHHHH---HHTS?SSS----?EEEEEE??????S??-?TTT??HHHHHHHHHTTHHHHHHHHHHHTT-------S??SEEEEEEEGGG-TT??T--T?HHHHHHHHHHHHHHHHHHHTT??------?EEEEE??BSSS  &nbspPDB/DSSP  
MODEL    xTYLVTGGLGGTGTLGLELARWLAEEGIGGRGEARHLVLLSRSAALPEEEAEKLLEESAPDAEAELASEAAESEAAETKARIALLHPAFAELEADLAGALDIIEVTVVAADELVADRDAVRALLDLLEEVEAEGxxxxxxxxxxxxxxxxxxxxxxxxxxxxxxxxxxxxxxxxxxxxxxxxxxxxxxxxxxxxxxxxxxxxxxxxxxxxxxxxxxxxxxxxxxxxxxxxxxxxxxxxxxxxxxxxxxxxxxxxxxx  &nbspHMMER3[PF08659.5\_KR\_seed]  
QUERY    xTVLVVG-AGE---MGKTVAKSLV-D----RG-VRAVLVANRT-Y---ER--AVELARDLGGEAV--------------RFDELV----DHLAR-S-DV--VVSATAAPHP--VIHVDDVREA---LRKRDRRSxxxxxxxxxxxxxxxxxxxxxxxxxxxxxxxxxxxxxxxxxxxxxxxxxxxxxxxxxxxxxxxxxxxxxxxxxxxxxxxxxxxxxxxxxxxxxxxxxxxxxxxxxxxxxxxxxxxxxxxxxxx   Q9UXR8|Glutamyl-tRNA\_reductase|HEM1\_METKA[169 258]  
  
  
**PF13241.1\_NAD\_binding\_7\_seed:**  

**E:9.13e-02[Original]  &nbspE:1.07e-04[Fold-critical]  &nbspE:9.26e-02[Remnant]  &nbspRatio[FC/R]:1.16e-03  &nbspClassification:TP**

STRUCT   EEE-??TT--?EEEEEEESHHHH--HHHHHHGG-GT--?-E-E--EE--------E-E---EE---------E?TTHHHHH?G-------------------------------GG????-??-------------??-----????--------?---------EE-------------------------------------------------E-----------?TT??TTS???SEEE?----------SS??GGGG??SST-T-----?----------?EE---------EEE---E??----S?--HHHHHH--HHH----HH--HHHH?TTS--EEE-ETT--------?G---GGBS-----------EE??E  &nbspPDB/DSSP  
MODEL    LFLTDLEGIEKRVLVVGGGEVALCKRKIRSLLEGAGPDAGKEVQLTVAWNAPVAVVFSDIIPEITPNDAAKELLTPELEELAALDADFDGASDKAILKNQRAGNGKVTKYDNKLLAEAGLPIRKGQQARFFEADNVELPDQPCVRFAPRLGIELLRLGAISVRSARENIVLENKIDTGANIENTELENTLDKRYSSNSNLKNISDDLEIITILRRNFITLIKDQRAYREPGDLLTLEEILKDKIDALSGVFDNIEEDKEEELQLNELEDPNESFGNDKSLSLACVADPSRVMNIINALVIKDNAAT----DDRTPELNERTIIAAILAKLAAERAKFGRGIYLLVNDVADVSAADRSGDPGGRELCDKENRALSTALEFIFPA  &nbspHMMER2[PF13241.1\_NAD\_binding\_7\_seed]  
QUERY    LG--SLHD--KTVLVVGAGEMGK--TVAKSLVD-RG--V-R-A--VL--------V-A---NR-------------TYE---------------------------------------RA-VE--------------L--------A-------RDLGGEAVRFDEL-------------------------------------------------V-------------DH----------------------------------L-A-----R----------SD----------VVV---SATAAPHPV--IHVDDV--REA----LR-KRD---RRS--PIL-IID--------IA---NPRD-----------VEEGV   Q9UXR8|Glutamyl-tRNA\_reductase|HEM1\_METKA[162 275]  
  

**E:7.38e-03[Original]  &nbspE:8.65e-08[Fold-critical]  &nbspE:3.39e+02[Remnant]  &nbspRatio[FC/R]:2.55e-10  &nbspClassification:FN**

STRUCT   EEE-??TT--?EEEEEEESHHHH--HHHHHHGG-GT--?-E-E--EE--------E-E---EE---------E?TTHHHHH?G-------------------------------GG????-??-------------??-----????--------?---------EE-------------------------------------------------E-----------?TT??TTS???SEEE?----------SS??GGGG??SST-T-----?----------?EE---------EEE---E??S?--HHHHHH--HHH----HH--HHHH?TTS--EEE-ETT--------?G---GGBS-----------EE??E  &nbspPDB/DSSP  
MODEL    xxxxxLKGIEKRVLVVGGGEVAACKRKVRKLLEGAGPEAGKEVQLTVAWNAPVANVFSDIIPExxxxxxxxxxxxxxxxxxxxxxxxxxxxxxxxxxxxxxxxxxxxxxxxxxxxxxxxxxxxxxxxxxxxxxxxxxxxxxxxxxxxxxxxxxxxxxxxxxxxxxxxxxxxxxxxxxxxxxxxxxxxxxxxxxxxxxxxxxxxxxxxxxxxxxxxxxxxxxxxxxxxxxxxxxxxxxxxxxxxxxxxxxxxxxxxxxxxxxxxxxxxxxxxxxxxxxxxxxxxxxxxxxxxxxxxxxxxxxxxxxxxxxxxxxxxxxxxxxxxxxxxxxxxxxxxxxxxxxxxxxxxxxxxxxxxxxxxxxxxxxxxxxxxxxxxxxxx  &nbspHMMER3[PF13241.1\_NAD\_binding\_7\_seed]  
QUERY    xxxxxLHD--KTVLVVGAGEMGK--TVAKSLVD-RG--V-R-A--VL--------V-A---NRxxxxxxxxxxxxxxxxxxxxxxxxxxxxxxxxxxxxxxxxxxxxxxxxxxxxxxxxxxxxxxxxxxxxxxxxxxxxxxxxxxxxxxxxxxxxxxxxxxxxxxxxxxxxxxxxxxxxxxxxxxxxxxxxxxxxxxxxxxxxxxxxxxxxxxxxxxxxxxxxxxxxxxxxxxxxxxxxxxxxxxxxxxxxxxxxxxxxxxxxxxxxxxxxxxxxxxxxxxxxxxxxxxxxxxxxxxxxxxxxxxxxxxxxxxxxxxxxxxxxxxxxxxxxxxxxxxxxxxxxxxxxxxxxxxxxxxxxxxxxxxxxxxxxxxxxxxxx   Q9UXR8|Glutamyl-tRNA\_reductase|HEM1\_METKA[165 199]  
  
  
**PF13460.1\_NAD\_binding\_10\_seed:**  

**E:2.40e-02[Original]  &nbspE:2.69e-06[Fold-critical]  &nbspE:8.56e-01[Remnant]  &nbspRatio[FC/R]:3.14e-06  &nbspClassification:TP**

STRUCT   EEEESTTSHHHHHHHHHHHHTT----?EEEEEE---S?-??----???---------?SSEEEE??T--T?H---HHHHHHHT---T--?SEEEE??------?TT---T??GG--------G-------HHH--H--HH----HHHHHHH-H---H-T--??-E--EE---E--E-??GG---G?E-----EETT-----EEG--GGSTT?------?GGGHHH-H----HHH--H-HH-----H-----HHH----T--T??--SS-EEEEEE??S-EEES-------?---??--????EE-----ESS--------B????-----T-T-----S?-----?EEEHHHHHHHHHHHHHH  &nbspPDB/DSSP  
MODEL    IAVIGATGKTGRRIVKEALARGKFKVHEVTALVLHGRNAPSVVPSKLPELNEASLFGPGVTPVQKDLALFDLSIVEDLAEALAMLPGKPFDAVVDAFRPLKAAGARSWEPPIDLTDSGPAKVDTLAVLPLFRDSLGGSVKTSLSHLLDALASGLIDARGESVRMR-PLVWAGVGGVLSSAGSLRLYRQSGHVDVPGLELVFFGLNIDDTPLFPELKRIREAIKPYSARPLARAKILALAAYGDSLEKEALKELLEQLLRAAAVSAKGLEDWTIVRPAADLFDALSESAATEGPGGGTETGTYELKTRSWGTEDEGNMVSILLIGDVLRLYPSATLNSRGEKEAGESPISRADVAAALVDELEN  &nbspHMMER2[PF13460.1\_NAD\_binding\_10\_seed]  
QUERY    VLVVGA-GEMGKTVAKSLVDRG----VRAVLVA--NRT-YE----RAVEL--ARD--LGGEAVRF----DEL-----VDH-LA---R--SDVVVSAT------AAP---HP--------------------VI--H--VD----DVREALR-K---R-D---R-RSPIL---I--I-DIAN---PRD-----VEEG-----VEN-------------IEDVEVRT-I----DDL--RVIA----RE-----NLE----R--R-R-KEI---------P-KVEKL------I---EE----ELST-----VEE-----------EL-----E-K-----LK-----ERRLVADVAKSLHEIKDR   Q9UXR8|Glutamyl-tRNA\_reductase|HEM1\_METKA[170 344]  
  
  
**PF01408.17\_GFO\_IDH\_MocA\_seed:**  

**E:8.97e-02[Original]  &nbspE:3.09e-04[Fold-critical]  &nbspE:4.18e-02[Remnant]  &nbspRatio[FC/R]:7.39e-03  &nbspClassification:TP**

STRUCT   EEEEEE??-SHH---HHHT---HHHHGGG?SSE-------EEEEEE?S?HHHHHHH----HHHHT??GGGEE?SSS----------GG-GGGG?T--T---??EEEE?S?T------TH----HHHHHHHHHHTT?--EEEE?SSS?S------SHHHHHHHHHHHHHHT----------??-EEE??  &nbspPDB/DSSP  
MODEL    LRVGIVGAAGRINLFGRRHCDRLRALLESQDGAKKLGPRLELVAVLDPDPARAEAVLKEKAESFGVPARKEKVYSDTKVYPSVEEYLEYELLADPAKDDDGIDAVIVATPNAVHGRGGLTPSPHFELALAALEAGKNAHVLCEKPLATGVPAEGTLEEAKELVELARKKGCKDEKRGKDAVVVLQVGF  &nbspHMMER2[PF01408.17\_GFO\_IDH\_MocA\_seed]  
QUERY    KTVLVVGA-GEM---GKTV---AKSLVDR--GV-------RAVLVANRTYERAVEL----ARDLGGE---AVRFDE----------LV-DHLA----R---SDVVVSATAA------PH-------------------PVI-------------HVDDVREALRKRDRRS----------PI-LIIDI   Q9UXR8|Glutamyl-tRNA\_reductase|HEM1\_METKA[168 265]  
  
  
**PF02254.13\_TrkA\_N\_seed:**  

**E:3.55e-02[Original]  &nbspE:1.01e-03[Fold-critical]  &nbspE:1.15e-02[Remnant]  &nbspRatio[FC/R]:8.78e-02  &nbspClassification:TP**

STRUCT   EEEE?-?SHHHHHHHHHHHHTT-?------------------EEEEEES?H--HH--HHHH--H-HH?--SSE--------EEE-S?TTSHHHHHHT-TTTT?S--EEEE?---?S-------?H----HHHHHH--HHHH-HH---TT???---EE--EE?SSTTH-----HHHH-HH--TT-----?--SEEE?H  &nbspPDB/DSSP  
MODEL    IIIIGAYGRVGRSLAEELSEEGHGVEKSFLSKLLPIDLEQIPDVVVIDKDEIDERKGVEELLEREEEGPDDTLVPGEAGAYVVVRGDATDEEVLEEALGIEDADCKAVIAASLPTGIEKNELDDDLLKIEANILI--VLLAKKELNPDGVKKPFYIILPARANDPEHLKPESAEKLKRRENLGRLIFMASIDEVISP  &nbspHMMER2[PF02254.13\_TrkA\_N\_seed]  
QUERY    VLVVG-AGEMGKTVAKSLVD-R-G----------------VR-AVLVANRTYERA--VELA--R-DLG-----------------GEAVRFDELVDH--LARSD--VVVSA---TA-------AP----HPVIHVDDVREA-LRKRDRRSPI---LI--IDIANPRDV----EEGV-EN--IE--------DVEVRT   Q9UXR8|Glutamyl-tRNA\_reductase|HEM1\_METKA[170 285]  
  
  
**PF03949.10\_Malic\_M\_seed:**  

**E:3.44e-02[Original]  &nbspE:1.75e-05[Fold-critical]  &nbspE:1.38e-08[Remnant]  &nbspRatio[FC/R]:1.27e+03  &nbspClassification:TP**

TM       ----------------------------------------ccc--ccccccc----------------------------------------------------------------------------------------------------------------------------------------------------------------------------------------------------------------------------------------------------------------------------------  &nbspTMSOC  
STRUCT   THHHHHHHHHHHHHHHHHHTS?---G------GG??EEEE??S--HHHHHHHHHHHHHHHHH-T??H---HHHHTTEEEEETTEE?BTT?-SS--?------?-----TTGGG----G?BS?????--------------?HHHHHHHH??SEEEE?S?-?TT-?S?HHHHHHHHHH?S------S?E-EEE??SSGGG?SS?HHHHHHHTTT??EEEESS????EE?TTS?EE??EE??GGGTHH--HHHHHHHHHT?SS??HHHHHHHHHHHHHT??HH--------------HHHH-T?SS??GGGHHHHHHHHHHHHHHHHH  &nbspPDB/DSSP  
MODEL    IQGTAAVVLAGLLNALKITGKPLIDLNRTAKASDQKIVFFGAGRCAAGIGIAELLVAAMVRETGLSESAFEEARKNIWMVDRKGLLTEDRKEDGKLDRLMLLNNGTMPPFKKPGLRYFARKTNEVKGWGRDDKHDDGDGGTLAEVVKGAKPDVLIGVSGPVPG-AFTEEIVRAMAEHTEAGGLGTRPI-IFALSNPTPKAEITPEDAYKWTAGRVLFATGSPFPPVELPNGRSDYPNQVNNVLIFPVSGIGLGALDVRARRITDEMFLAAAEALAELVTEEVPDVVASAYGGEKLELGEYGYIIPPLFDIREVSPRVAVAVAKAAV  &nbspHMMER2[PF03949.10\_Malic\_M\_seed]  
QUERY    ----GAVSIGS--AAVELAERELGSL------HDKTVLVVGAG--EMGKTVAKSLVDR-----GVR----A-------------VLVANR--T---------Y-----ERAVE----LARDLGGEAVR-FD---------ELVDHLARS--DVVVSATA-APHPVIHVDDVREALRKRD----RRSPILIIDIANPRD---------VEEGVENI--------EDVEV-------------------------------RTIDDL------RVIARENLER--------------RRKE----IPKVEKLI---EEELSTVEEELE   Q9UXR8|Glutamyl-tRNA\_reductase|HEM1\_METKA[146 323]  
  
  
**PF07991.7\_IlvN\_seed:**  

**E:1.65e-04[Original]  &nbspE:1.84e-09[Fold-critical]  &nbspE:4.47e+02[Remnant]  &nbspRatio[FC/R]:4.12e-12  &nbspClassification:TP**

STRUCT   HHHT-S?EEEE??SHHHHHHHHHHHHTT?-?EEEE??TT?----H-HHHHHHHTT?EEE?HHHHHH---T?SEEEE?S?HHHHHHHHHHHTGGG??TT?--EEEES??HHHHTTSS???TT?EEEEEEESS?SHHHHHHHHTT????EEEEEE--E?SS-S?HHHHHHHHHHHTTHHHH?  &nbspPDB/DSSP  
MODEL    xLKGAKKIAVIGYGSQGHAQALNLRDSGLKDVVVGLREGSILEKKKSVEKAKKDGFEVLTVAEAVK---KADVVMIxxxxxxxxxxxxxxxxxxxxxxxxxxxxxxxxxxxxxxxxxxxxxxxxxxxxxxxxxxxxxxxxxxxxxxxxxxxxxxxxxxxxxxxxxxxxxxxxxxxxxxxx  &nbspHMMER3[PF07991.7\_IlvN\_seed]  
QUERY    xLHD-KTVLVVGAGEMGKTVAKSLVDRGVRAVLVANRTYE----R-AVELARDLGGEAVRFDELVDHLARSDVVVSxxxxxxxxxxxxxxxxxxxxxxxxxxxxxxxxxxxxxxxxxxxxxxxxxxxxxxxxxxxxxxxxxxxxxxxxxxxxxxxxxxxxxxxxxxxxxxxxxxxxxxxx   Q9UXR8|Glutamyl-tRNA\_reductase|HEM1\_METKA[165 233]  
  
  
**PF05201.10\_GlutR\_N\_seed:**  

**E:1.85e-56[Original]  &nbspE:9.89e-26[Fold-critical]  &nbspE:7.73e-07[Remnant]  &nbspRatio[FC/R]:1.28e-19  &nbspClassification:TP**

STRUCT   EEEETTTS?HHHHHHHS???-TTHHH------HH-HHHHT?S----EEEEEEETTEEEEE----EES?TT-?H-------------------------------------------HHHHH-----TT---?T-T-----?EEEE---HHHHHHHHHHHHTTTT----SSSTT?HHHHHHHHHHHHHHH-----HHT???HHHHHHHHHHHHHHHHHHHHSS  &nbspPDB/DSSP  
MODEL    VGLNHKTAPVEIREKLAFSPDEELEENQALQVELKKALPGVDYASREAVILSTCNRTEIYFSSEAYSSVDLDPLSAVSEAGLEKKNDLILIDESVSANNSANLSSTIIVDLILKLKEWLAEIFHGLLDGASLEPEVLEPYLYVYEVYEGEEAVRHLFRVASGLDLVWISMVLGEPQILGQVKEAYALARNFYQSEAGTVGKILNRLFQKAFSVAKRVRTETA  &nbspHMMER2[PF05201.10\_GlutR\_N\_seed]  
QUERY    VGITHKEAEVEELEKARFES-DEAVR------DI-VESFGLS----GCVLLQTCNRVEVY----ASGARD-RA-------------------------------------------EELGD-----LI---HD-D-----AWVKR---GSEAVRHLFRVACGLE----SMMVGEQEILRQVKKAYDRAA-----RLGTLDEALKIVFRRAINLGKRAREETR   Q9UXR8|Glutamyl-tRNA\_reductase|HEM1\_METKA[7 142]  
  

**E:4.90e-39[Original]  &nbspE:3.47e-48[Fold-critical]  &nbspE:1.48e+04[Remnant]  &nbspRatio[FC/R]:2.34e-52  &nbspClassification:TP**

STRUCT   EEEETTTS?HHHHHHHS???-TTHHH------HH-HHHHT?S----EEEEEEETTEEEEE----EES?TT-?H-------------------------------------------HHHHH-----TT---?T-T-----?EEEE---HHHHHHHHHHHHTTTT----SSSTT?HHHHHHHHHHHHHHH-----HHT???HHHHHHHHHHHHHHHHHHHHSS  &nbspPDB/DSSP  
MODEL    IGVNHKTAPVELREKLAFSEEEELEESLALAEQLKKSEEGIEKASAEAVILSTCNRTEIYALVEASSSSESDALLENSEAAVEKKSELSVIESSVTANNSANLSSTIISAHILKIKEWLAEIFKQLLSEESLELEELEEYLYVLELYEGEEAVRHLFRVASGLDLVWISMVLGEPQILGQVKRAYELAKNFYQSEAGTLGKILNRLFQKAFAVAKRVRTETA  &nbspHMMER3[PF05201.10\_GlutR\_N\_seed]  
QUERY    VGITHKEAEVEELEKARFESDEAVRD--IVE------SFGLS----GCVLLQTCNRVEVY----ASGARD-----------RA---------------------------------EELGD-------------L-IHDDAWVKR---GSEAVRHLFRVACGLE----SMMVGEQEILRQVKKAYDRAA-----RLGTLDEALKIVFRRAINLGKRAREETR   Q9UXR8|Glutamyl-tRNA\_reductase|HEM1\_METKA[7 142]  
  
  
**PF00745.15\_GlutR\_dimer\_seed:**  

**E:1.57e-18[Original]  &nbspE:3.51e-10[Fold-critical]  &nbspE:3.56e-04[Remnant]  &nbspRatio[FC/R]:9.86e-07  &nbspClassification:TP**

STRUCT   HHHHHHHH---HHHHHHHHHHHHH-H----HHHHHHHHHHHHHHHHHHHHHHHHS?---S?----??-??-????TTHHHHHHHHHHHHHHHHHHS-S?-TTTT--THH-----HHHHHHHHH--H-----T  &nbspPDB/DSSP  
MODEL    EKAEAIIESINEEVEEFMEWLRSLQEPVGGVVPTIRALREKAEEIREEELERALKKLGLLDKYGNLGEDPVEEVLEKLARSLTNKLLHAPTVALKECAARAEGDGFDDLTLVENLEALRRLFGSDLAQSNHD  &nbspHMMER2[PF00745.15\_GlutR\_dimer\_seed]  
QUERY    PKVEKLIE---EELSTVEEELEKL-K----ERRLVADVAKSLHEIKDRELERALRR---LK----TG-DP-ENVLQDFAEAYTKRLINVLTSAIME-LP-DEYR--RAA-----CRALRRASE--L-----N   Q9UXR8|Glutamyl-tRNA\_reductase|HEM1\_METKA[305 403]  
  

**E:1.51e-14[Original]  &nbspE:7.45e-19[Fold-critical]  &nbspE:4.69e+02[Remnant]  &nbspRatio[FC/R]:1.59e-21  &nbspClassification:TP**

STRUCT   HHHHHHHH---HHHHHHHHHHHHH-H----HHHHHHHHHHHHHHHHHHHHHHHHS?---S?----??-??-????TTHHHHHHHHHHHHHHHHHHS-S?-TTTT--THH-----HHHHHHHHH--H-----T  &nbspPDB/DSSP  
MODEL    xQAEAIIESINEEVEEFEEWLKSLQEPVGGVVPLIRALREKAEEIREEELEKALKKLGLLAKKAELGEDPVEEVLEKLARSLTNKLLHGPTKALREAAARADGDAADELTLVENLEALRRLFDSDLxxxxxx  &nbspHMMER3[PF00745.15\_GlutR\_dimer\_seed]  
QUERY    xKVEKLIE---EELSTVEEELEKL-K----ERRLVADVAKSLHEIKDRELERALRR---LK-----TGDP-ENVLQDFAEAYTKRLINVLTSAIME-LP-DEYR--RAA-----CRALRRASE--Lxxxxxx   Q9UXR8|Glutamyl-tRNA\_reductase|HEM1\_METKA[306 402]

---

**User sequence**  

Q9UXR8|Glutamyl-tRNA\_reductase|HEM1\_METKA  
MEDLVCVGITHKEAEVEELEKARFESDEAVRDIVESFGLSGCVLLQTCNRVEVYASGARDRAEELGDLIHDDAWVKRGSEA  
AVRHLFRVACGLESMMVGEQEILRQVKKAYDRAARLGTLDEALKIVFRRAINLGKRAREETRISEGAVSIGSAAVELAERE  
ELGSLHDKTVLVVGAGEMGKTVAKSLVDRGVRAVLVANRTYERAVELARDLGGEAVRFDELVDHLARSDVVVSATAAPHPV  
VIHVDDVREALRKRDRRSPILIIDIANPRDVEEGVENIEDVEVRTIDDLRVIARENLERRRKEIPKVEKLIEEELSTVEEE  
ELEKLKERRLVADVAKSLHEIKDRELERALRRLKTGDPENVLQDFAEAYTKRLINVLTSAIMELPDEYRRAACRALRRASE  
ELNG

---

**Main reference**  

1) Wing-Cheong Wong, Sebastian Maurer-Stroh, Birgit Eisenhaber, Frank Eisenhaber, 2014,
*The necessity of dissecting similarity scores for inferring homology: an essential issue in protein function prediction and annotation*
, BMC Bioinformatics, 15(1):166, doi:10.1186/1471-2105-15-166

**Relevant references**  

1) Wing-Cheong Wong, Sebastian Maurer-Stroh, Georg Schneider, Frank Eisenhaber, 2012,
*Transmembrane helix: simple or complex*
, Nucleic Acids Research (Web Server issue), doi:10.1093/nar/gks379  
  
2) Wing-Cheong Wong, Sebastian Maurer-Stroh, Frank Eisenhaber, 2011,
*Not all transmembrane helices are born equal: Towards the extension of the sequence homology concept to membrane proteins*
, Biology Direct, 6(57), doi:10.1186/1745-6150-6-57  
  
3) Wing-Cheong Wong, Sebastian Maurer-Stroh, Frank Eisenhaber, 2011,
*The Janus-faced E-values of HMMER2: Extreme value distribution or logistic function*
, Journal of Bioinformatics and Computational Biology, 9(1), doi:10.1142/S0219720011005264  
  
4) Wing-Cheong Wong, Sebastian Maurer-Stroh, Frank Eisenhaber, 2010,
*More than 1001 problems with protein domain databases: transmembrane regions, signalpeptides and the issue of sequence homology*
, PLoS Computational Biology, 6(7), doi:10.1371/journal.pcbi.1000867

**Contact**  

wongwc@bii.a-star.edu.sg

  

24-03-2015 17:51:43 Copyrights Bioinformatics Institute A\*STAR

  
