# Supplementary material for: dissectHMMER: a HMMER-based score dissection framework that statistically evaluates fold-critical sequence segments for domain fold similarity
Source: Biol Direct. 2015 Aug 1;10:39. doi: 10.1186/s13062-015-0068-3 (PMC4521371; doi:10.1186/s13062-015-0068-3)
Supplement: Additional file 7: — The web output of dissectHMMER search results for the protein sequence TIP12_MAIZE. (ZIP 9 kb) [file 13062_2015_68_MOESM7_ESM.zip › 5864776041655568_add7.html]

  
  

dissectHMMER


# dissectHMMER results

---

**Overview of Sequence Annotation**  

  
Domain(s) to sequence segment[112,243]  
Domain name?                     &nbspPDB?    &nbspLength?  &nbspSeq range?  &nbspScore[FC/R]?  &nbspFPR[FC/R]?  &nbspScore[FC]?  &nbspFPR[FC]?  &nbspDomaincoverage?   **Total FPR?**   **Hits'classification?**     
PF00230.15\_MIP\_seed           ?  &nbsp1YMG|A  &nbsp296      &nbsp13,234      &nbsp1             &nbsp0.00        &nbsp1           &nbsp0.00      &nbsp1                 **0**   **TP,TP**  
PF00654.15\_Voltage\_CLC\_seed   ?  &nbsp2HLF|A  &nbsp730      &nbsp1,236       &nbsp0.345         &nbsp0.28        &nbsp0.55        &nbsp0.01      &nbsp1                 **0.29**   **FN,TP**  
PF01226.12\_Form\_Nir\_trans\_seed?  &nbsp4FC4|A  &nbsp366      &nbsp17,238      &nbsp0.325         &nbsp0.32        &nbsp0.49        &nbsp0.03      &nbsp1                 **0.35**   **TP**  
PF07331.6\_TctB\_seed           ?  &nbsp-       &nbsp266      &nbsp112,243     &nbsp0.385         &nbsp0.21        &nbsp0.42        &nbsp0.15      &nbsp1                 **0.36**   **TP**  
PF07155.7\_ECF-ribofla\_trS\_seed?  &nbsp4HZU|S  &nbsp196      &nbsp57,190      &nbsp0.235         &nbsp0.48        &nbsp0.435       &nbsp0.13      &nbsp1                 **0.61**   **TP**  
PF06912.6\_DUF1275\_seed        ?  &nbsp-       &nbsp290      &nbsp57,216      &nbsp0.225         &nbsp0.50        &nbsp0.435       &nbsp0.13      &nbsp1                 **0.63**   **TP**  
PF01384.15\_PHO4\_seed          ?  &nbsp-       &nbsp1203     &nbsp19,231      &nbsp0.15          &nbsp0.63        &nbsp0.49        &nbsp0.03      &nbsp1                 **0.66**   **TP**  
PF09490.5\_CbtA\_seed           ?  &nbsp-       &nbsp333      &nbsp44,231      &nbsp0.11          &nbsp0.73        &nbsp0.47        &nbsp0.06      &nbsp1                 **0.79**   **TP**  
PF04211.8\_MtrC\_seed           ?  &nbsp-       &nbsp283      &nbsp6,246       &nbsp0.075         &nbsp0.83        &nbsp0.44        &nbsp0.11      &nbsp1                 **0.94**   **TP**  
PF02028.12\_BCCT\_seed          ?  &nbsp2WSW|A  &nbsp722      &nbsp28,242      &nbsp0.01          &nbsp0.98        &nbsp0.495       &nbsp0.03      &nbsp1                 **1.01**   **TP**  
PF13303.1\_PTS\_EIIC\_2\_seed     ?  &nbsp-       &nbsp464      &nbsp19,248      &nbsp0.03          &nbsp0.93        &nbsp0.435       &nbsp0.13      &nbsp1                 **1.06**   **TP**

---

**Alignments**  
  
**PF00230.15\_MIP\_seed:**  

**E:1.46e-127[Original]  &nbspE:7.46e-33[Fold-critical]  &nbspE:1.05e-12[Remnant]  &nbspRatio[FC/R]:7.10e-21  &nbspClassification:TP**

TM       -------------cccccccccccccccc--ccccc-------------------------------------------ccccccccccccccccccccc--cccc--------------------cccccccccccccccccccccc------------------------------------ccccccccccccccccccccccc--------------ccccccccccccccccccccccc---------------------------------cccccccccccccccccc-  &nbspTMSOC  
STRUCT   ??????H--HHHHHHHHHHHHHHHHHHHH--HHHHHHHS??-------------------------???---------HHHHHHH-HHHHHHHHHHHHHH--HH?????HHHHHHHHHHS???GGGHHHHHHHHHHHHHHHHHHHHHHTHHHHHHHHHHTT??TTSGGGHHHHTTTS????TT??HHHHHHHHHHHHHHHHHHHHHHH?TTSSS??GG---GHHHHHHHHHHHHHHHTHHHH?????HHHHHHHHHHHHHTTTTTHHHH-?SSSS?TTHHHHHHHHHHHHHHHHHH  &nbspPDB/DSSP  
MODEL    ELRSVSFTYLRAVIAEFLATLLFVFAGVGVVSALGVKKAYSQDNFNVALDNLNVTGSSAETIDAMKSLATLVSSCAGVGLLAVALAFGLALFVLVYCVGHSAISGAHLNPAVTLALLVGRRISLLRAIYYIIAQLLGAIVAAALLKGVTNGLLFEAESDWWIVRGSVESYLRAGGFANSLAPGVNAGQAFVVEIILTFQLVYTVFATTDDRRRGSLGSIPILAPLAIGFIVALNHLAGGPYTGASMNPARSFGPAVVTWKAGWGFVAKTLGVRDWDDHWVYWVGPLVGAALAALVY  &nbspHMMER2[PF00230.15\_MIP\_seed]  
QUERY    ELSHPDT--AKAAVAEFISTLIFVFAGSG--SGMAFSKLTD-------------------------GGAATPA-----GLIAASLAHALALFVAVSVGAN--ISGGHVNPAVTFGAFVGGNISLLKALVYWVAQLLGSVVACLLLKIATGG-------------------AALGAFS--LSAGVGAMNAVVLEMVMTFGLVYTVYATAVDPKKGDLGVI---APIAIGFIVGANILAGGAFDGASMNPAVSFGPAVVTGV--------------WENHWVYWVGPLAGAAIAALVY   Q9ATM0|Aquaporin|TIP12\_MAIZE[13 234]  
  

**E:8.50e-73[Original]  &nbspE:1.43e-72[Fold-critical]  &nbspE:4.54e-01[Remnant]  &nbspRatio[FC/R]:3.15e-72  &nbspClassification:TP**

TM       -------------cccccccccccccccc--ccccc-------------------------------------------ccccccccccccccccccccc--cccc--------------------cccccccccccccccccccccc------------------------------------ccccccccccccccccccccccc--------------ccccccccccccccccccccccc---------------------------------cccccccccccccccccc-  &nbspTMSOC  
STRUCT   ??????H--HHHHHHHHHHHHHHHHHHHH--HHHHHHHS??-------------------------???---------HHHHHHH-HHHHHHHHHHHHHH--HH?????HHHHHHHHHHS???GGGHHHHHHHHHHHHHHHHHHHHHHTHHHHHHHHHHTT??TTSGGGHHHHTTTS????TT??HHHHHHHHHHHHHHHHHHHHHHH?TTSSS??GG---GHHHHHHHHHHHHHHHTHHHH?????HHHHHHHHHHHHHTTTTTHHHH-?SSSS?TTHHHHHHHHHHHHHHHHHH  &nbspPDB/DSSP  
MODEL    xLSSVKFTYLRAVLAEFLATLLFVFIGVGVVSALALKKKIQQDNFNVALDNLNVTGSSAETIDAMKSLASLVSSVAGVTLLAVALAFGLALAVLVYLAAGSAISGAHLNPAVTLALLVARKISLLRAILYIVAQLLGAIVAAALLKLLTKGLLQEAESDWWLNRGSVESESRAGLFANSLKPGLSAGQAFVVEIILTFVLVLTVFALTDDKRKLSLGSIPILAPLAIGLLVALIILVGIPLTGAAMNPARSLGPAVVLNKVGWGFDAKMLWVRDFEDHWVYLVGPLIGALLGALVY  &nbspHMMER3[PF00230.15\_MIP\_seed]  
QUERY    xLSHPDT--AKAAVAEFISTLIFVFAGSG--SGMAFSKLTD-------------------------GGA-----ATPAGLIAASLAHALALFVAVSVGAN--ISGGHVNPAVTFGAFVGGNISLLKALVYWVAQLLGSVVACLLLKIATGGA-------------------ALGAFS--LSAGVGAMNAVVLEMVMTFGLVYTVYATAVDPKKGDLGV---IAPIAIGFIVGANILAGGAFDGASMNPAVSFGPAVVTGV--------------WENHWVYWVGPLAGAAIAALVY   Q9ATM0|Aquaporin|TIP12\_MAIZE[14 234]  
  
  
**PF00654.15\_Voltage\_CLC\_seed:**  

**E:3.89e-01[Original]  &nbspE:7.03e-05[Fold-critical]  &nbspE:4.50e-06[Remnant]  &nbspRatio[FC/R]:1.56e+01  &nbspClassification:FN**

TM       ----------------------------------------------------------------cccccccc--cccccccccc-----------------------------------------------------ccccccccccccccccccccccccc---------------------------------cc----cccccccccccccccccc--------cc---------------------------------------------cccccccccccccccccccccccccc------------------------------------cccccccccc---cc----cccc----cccccccc-------------------cccccc-----------------------------------------------------------------------------------------------------------------------------------------------------------------------------------cccccccccccccccccccccccccccccccccccccccccccccc-----cccccccc--------------------------------------------ccccccccccccccccccccccccccc-----ccccccccccccccccccc--  &nbspTMSOC  
STRUCT   HHHHHH-------HHHHHHH-?GGG---S---S-?SHHHHHHHH---TT?S?-?-----?HHHHHHHHHHHH--HHHHHTT?S?BTHHHHHHHHHHHHHHH--H-----------HT------TT?--?SHH-HHHHHHHHHHHHHHHHHHT?HHHHHHHHHH-------------T-TS-?SSS???-------??----HHHHHHHHHHHHHHHHHH--------H?S?--?S-----------??-----?????----------?---??GGGHHHHHHHHHHHHH-HHHHHHHHHHHHH-H-----HHHHHHTT?HHH-------------HHHHHHHHHH---HH----HHHH----HHS-----------------------G-G--GSS?ST-------TH---------------------------------------------------------------------------------------------------------HHHHH---HT?---------------------------------------------------S?HHHHHHHHHHHHHHHHHHHHTT?SSBSHHHHHHHHHHHHH-HH-----H-HHHHHH-------------------------?GG-------GT??T----HHHHHHHHTTHHHHTT??HHHHHHHHHHHH?-?GGGHHHHHHHHHHHHHHHHT  &nbspPDB/DSSP  
MODEL    IGGLLALVSLIAIGLLVKRFRAPEAFIAARRRGESGIPQVIAALLLHHGKKGYRRDRRLLPLRVLLVKFLGSICLLTIGSGGSLGREGPSVQIGAAIGSGLPSGQIFSKRYRSKIRRCHVHFLLKLFGSRNDRRRRELIAAGAAAGIAAAFNAPLAGVLFALEVGKFIEILTNSFKELLLGRDFRYSLFNESSPVRAGWGQLLPVLVASVVAALVSRLLLKLDNRGSFGNELGPLFPSFDLTGLFNFGEISAAVPSLFEVNFPFPYDLLSSKLSLLELPLFILLGILCGLGLGALFVRLLLKVEARGLSPGLFRRLRRKKPIPLLCNLLKVDYKPPPLLRPALGGLGMGLVGIVLGLLGYLGILLLASLLGAAPFQDMEDRGDVAVAGAPLEGRVLGGGYLIASVGNGLLLSKLFTPSSLFLSGSLDTLKYSSSSAVADVYVDKVSRPVVAVVRSGRNVTTIGFYGMNTLLDSADSIVVSDIVDITNVAEDPNNTEKVLKNLKAFLCDNCTGSPIQLLLCEDSGNTSMQLLSVLASDAASSAAASPWNLLAGGGNVSGSASTAVAAVFNESSTTGLLLLLLLLLLLLLKLLATALSLGSGAPGGIFAPSLFIGAALGATLLWRLLEGLLLLALLQFLSRKSSSSSSAFSTLSL-PAAFPFPIIFGEIACGGIAPSIPPGAFALVGMAAFLAAVTRAPLTAIVLVFELTGVSYSLLLPLMLAVLIAYLVSRL  &nbspHMMER2[PF00654.15\_Voltage\_CLC\_seed]  
QUERY    MPVSRI------------AV--GAP-------G---------EL---SHPDT-A--------KAAVAEFIST--LI-------------FVFAGSGSGMAF--S-----------KL-------------TD-----------------------GGAATPAG-------------LIAA------SL-------AH----ALALFVAVSVGANISGGH--------VNPA--VT-----------FG----AFVG----------------GNISLLK----------A-LVYWVAQLLGSVV-A--------------CLL--------------L-KIATGGA---AL----GAFS----LS--------------------------A------GV-------GA---------------------------------------------------------------------------------------------------------MNAVV---LE-----------------------------------------------------MVMT---FGLVYTVYATAVDPKKGDLGVIAPI-----AIGF-IV-----GANILAGG------------AFDGASMNPAVSFGP-----------------------------AVVTG--------VWE--N-HWVYWVGPLAGAAIAALVYDI   Q9ATM0|Aquaporin|TIP12\_MAIZE[1 236]  
  

**E:6.52e-05[Original]  &nbspE:2.47e-08[Fold-critical]  &nbspE:1.48e+04[Remnant]  &nbspRatio[FC/R]:1.67e-12  &nbspClassification:TP**

TM       ----------------------------------------------------------------cccccccc--cccccccccc-----------------------------------------------------ccccccccccccccccccccccccc---------------------------------cc----cccccccccccccccccc--------cc---------------------------------------------cccccccccccccccccccccccccc------------------------------------cccccccccc---cc----cccc----cccccccc-------------------cccccc-----------------------------------------------------------------------------------------------------------------------------------------------------------------------------------cccccccccccccccccccccccccccccccccccccccccccccc-----cccccccc-------------------------------------------ccccccccccccccccccccccccccc-----ccccccccccccccccccc--  &nbspTMSOC  
STRUCT   HHHHHH-------HHHHHHH-?GGG---S---S-?SHHHHHHHH---TT?S?-?-----?HHHHHHHHHHHH--HHHHHTT?S?BTHHHHHHHHHHHHHHH--H-----------HT------TT?--?SHH-HHHHHHHHHHHHHHHHHHT?HHHHHHHHHH-------------T-TS-?SSS???-------??----HHHHHHHHHHHHHHHHHH--------H?S?--?S-----------??-----?????----------?---??GGGHHHHHHHHHHHHH-HHHHHHHHHHHHH-H-----HHHHHHTT?HHH-------------HHHHHHHHHH---HH----HHHH----HHS-----------------------G-G--GSS?ST-------TH---------------------------------------------------------------------------------------------------------HHHHH---HT?---------------------------------------------------S?HHHHHHHHHHHHHHHHHHHHTT?SSBSHHHHHHHHHHHHH-HH-----H-HHHHHH------------------------?GG-------GT??T----HHHHHHHHTTHHHHTT??HHHHHHHHHHHH?-?GGGHHHHHHHHHHHHHHHHT  &nbspPDB/DSSP  
MODEL    xxxxxxxxxxxxxxxxxxxxxxxxxxxxxxxxxxxxxxxxxxxxxxxxxxxxxxxxxxxxxxxxxxxxxxxxxxxxxxxxxxxxxxxxxxxxxxxxxxxxxxxxxxxxxxxxxxxxxxxxxxxxxxxxxxxxxxxxxxxxxxxxxxxxxxxxxxxxxxxxxxxxxxxxxxxxxxxxxxxxxxxxxxxxxxxxxxxxxxxxxxxxxxxxxxxxxxxxxxxxxxxxxxxxxxxxxxxxxxxxxxxxxxxxxxxxxxxxxxxxxxxxxxxxxxxxxxxxxxxxxxxxxxxxxxxxxxxxxxxxxxxxxxxxxxxxxxxxxxxxxxxxxxxxxxxxxxxxxxxxxxxxxxxxxxxxxxxxxxxxxxxxxxxxxxxxxxxxxxxxxxxxxxxxxxxxxxxxxxxxxxxxxxxxxxxxxxxxxxxxxxxxxxxxxxxxxxxxxxxxxxxxxxxxxxxxxxxxxxxxxxxxxxxxxxxxxxxxxxxxxxxxxxxxxxxxxxxxxxxxxxxxxxxxxxxxxxxxxxxxxxxxxxxxxxxxxxxxxxxxxxxxxxxxxxxxxxxxxxxxxxxxxxxxxxxxxxxxxxxxxxxxLLVLKFLATALTLGSGLPGGIFVPSLVIGAALGRTLVWRLLEGLLVLALLESLSRKSSSSSSAFSTLSLPARKLFPVLLGESASKGDASSIDPGAYALIGAAAFLAGVTRAPLTAIVIVFELTGADxxxxxxxxxxxxxxxxxxxx  &nbspHMMER3[PF00654.15\_Voltage\_CLC\_seed]  
QUERY    xxxxxxxxxxxxxxxxxxxxxxxxxxxxxxxxxxxxxxxxxxxxxxxxxxxxxxxxxxxxxxxxxxxxxxxxxxxxxxxxxxxxxxxxxxxxxxxxxxxxxxxxxxxxxxxxxxxxxxxxxxxxxxxxxxxxxxxxxxxxxxxxxxxxxxxxxxxxxxxxxxxxxxxxxxxxxxxxxxxxxxxxxxxxxxxxxxxxxxxxxxxxxxxxxxxxxxxxxxxxxxxxxxxxxxxxxxxxxxxxxxxxxxxxxxxxxxxxxxxxxxxxxxxxxxxxxxxxxxxxxxxxxxxxxxxxxxxxxxxxxxxxxxxxxxxxxxxxxxxxxxxxxxxxxxxxxxxxxxxxxxxxxxxxxxxxxxxxxxxxxxxxxxxxxxxxxxxxxxxxxxxxxxxxxxxxxxxxxxxxxxxxxxxxxxxxxxxxxxxxxxxxxxxxxxxxxxxxxxxxxxxxxxxxxxxxxxxxxxxxxxxxxxxxxxxxxxxxxxxxxxxxxxxxxxxxxxxxxxxxxxxxxxxxxxxxxxxxxxxxxxxxxxxxxxxxxxxxxxxxxxxxxxxxxxxxxxxxxxxxxxxxxxxxxxxxxxxxxxxxxxxxLAHALALFVAVSVGANISGGHVNPAVTFGAFVGG-NI-SLLKA-LV-YWV--------------------AQLLGSVVAC----LL--LKI-ATGGAALGAFSLSAGVGA--MNAVVLEMVMTF-Gxxxxxxxxxxxxxxxxxxxx   Q9ATM0|Aquaporin|TIP12\_MAIZE[63 154]  
  
  
**PF01226.12\_Form\_Nir\_trans\_seed:**  

**E:3.54e-03[Original]  &nbspE:3.71e-06[Fold-critical]  &nbspE:2.29e-06[Remnant]  &nbspRatio[FC/R]:1.62e+00  &nbspClassification:TP**

TM       ------------------------ccccccccccccccccccccccccc-----------------------------------cccccccccccccccccccccccccccccc--------------------ccccccccccccccccccccccccc-------------------------------------------------------------------------cccccccccccccccccccccccc---------------ccccccccccccccccccccccccccccc-----------------------------------------------ccccccccccccccccccccccccccc-  &nbspTMSOC  
STRUCT   ???????????-????---????HHHHHHHHHHHHHHHHHHHHHHHHHHTTT--------------TSS?H----------HHHHHHHHHH-HTHHHHHHHHHT???TTTTHH-HHH--HHTTT?--??H-HHHTTTHHHHHHHHHHHHHHHHHHHHHH---TGGG---------TTTT--------HHHHHHHHHHH------HT--T------?S-?-------------HHHHHHHHHHHHHHHHHHHHHHT-T?-----?SH--HHHHHTTHHHHHHHHHTT???HHHHHHHHHHH-------HH--H-HHH??HHHHHHTT------??GGGGTT?S-HHHHHHHTHHHHHHHHHHHHHHHHHHHHHHHH  &nbspPDB/DSSP  
MODEL    PAEIAEAAVAAIGVKKEALARLPLLKLLLLGILAGAFIGFGALLATVVATGLPTTKVTAADAAAGAAAAGPLIGIDSVEFAGLAKLLGGLVGFPVGLILVVLGGAELFTGNTLTLTL--ALLEKKTLITLFGDLLRNWGIVYIGNLIGALFVAYLLAYSGPNGGLTGTAAKSTGALDDGDHGAFTQLAVGAAAIKIAELYCSWKHKVTVSYEAAGSLISTGESISAGLVIGTFWEAFLRGILCNWLVCLAVWLALLAA-----KDVPNAGKILAIWLPIMAFVASGFEHSVANMFLIPAGIFHGDNPIF--LGGANVPVVFAAAIGQAAAGAVSPAQLADLTIWGGFILKNLIPVTLGNIVGGAVFVGLLYWFIY  &nbspHMMER2[PF01226.12\_Form\_Nir\_trans\_seed]  
QUERY    PDT-----AKA-AVAE---------------FISTLIFVFAGSGSGMAFSKL------------TDGGAAT----------PAGLIAASLA-HALALFVAVSVGANISGGHVNPAVTFGAFVGGN--ISL-LKALVYWV----AQLLGSVVACLLLKIA---TGGA----------ALG--------AFSLSAGVGAM------NA--V------VL-E-------------MVMTF-----------GLVYTVY-ATAVDPKKGD------LGVIAPI----AIGF---IVGANILAGG-------AFDGA-SMNPAVSFGPAVVT--------------GVWENHWVYWVGP------LAGAAIAALVYDIIF   Q9ATM0|Aquaporin|TIP12\_MAIZE[17 238]  
  
  
**PF07331.6\_TctB\_seed:**  

**E:2.96e-02[Original]  &nbspE:1.25e-03[Fold-critical]  &nbspE:1.00e-02[Remnant]  &nbspRatio[FC/R]:1.25e-01  &nbspClassification:TP**

TM       ----cccccc--ccccccccccccccc----------------ccccccccccccccccccccc-------------------------------------------------------------cccccccccccccccccccccc------------ccccc----------ccccccccc------------cccccccccc--------------------ccc---cccc------cccccc--cccc---cccccc----  &nbspTMSOC  
STRUCT   CCHHHHHHHH11HHHHHHHHHHH---------------------HHHHHHHHHHHHHHHHHHHHH-----------------------------------------H11111111HH11111111HHHHHHHHH11HHHHHHH1111------------1111H1111111111HHHHHHHHH------------HHH1HHHHHH------------------HHHHH111HHHH111111HHHHHH11HHHH111HHHHCCCC1C  &nbspPredictedSECSTR  
MODEL    RADFVAGLLLAFLALGLFFAWGALLDLPIGTAGAVKEDRMGPGYFPFILGILLALLGAALLVQALRGGRKEAPPSMDLDESEAIATVADELAEAEEREDGEPLGKVDERELLATVWRVRALPAVIRVLLVLGALALLLFALLLAGLPLRIPREGLPDLISIGLEVFALGSLFAPPLGFVIATFPFRGTSCGNAVDLLLGFIAALALGEAEGQLDGGERGEKKRWLKALRLVLIAVSAIAWLVLSLLVVFYLVFGSLVYGLGLPLKP  &nbspHMMER2[PF07331.6\_TctB\_seed]  
QUERY    LGSVVACLLL--KIATGGAALGA-FSLSAGVGA-----------MNAVVLEMVMTFGLVYTVYATAV-----------------------------DPKKGDLGVIA--------PI--------AIGFIVGAN--ILAGGAF--------------------D----------GASMNPAVS------------FGP-AVVTGVWE---------------NHWVYW---VGPL------AGAAIA--ALVY---DIIFIGQR-P   Q9ATM0|Aquaporin|TIP12\_MAIZE[112 243]  
  
  
**PF07155.7\_ECF-ribofla\_trS\_seed:**  

**E:8.10e-02[Original]  &nbspE:8.07e-04[Fold-critical]  &nbspE:2.41e-05[Remnant]  &nbspRatio[FC/R]:3.35e+01  &nbspClassification:TP**

TM       ------cccccccccccccccccc---ccccc------------cccccccccccccccccccccccccc--------cccccccccccccccccccccccc----------------------ccccccccccccccccccc---ccccc--------------cccccccccccccccccccccccc--ccc--  &nbspTMSOC  
STRUCT   ??-?TTHHHHHHHHHHHHHHHHH-H--???B?S--SS?B-??TTHHHHHHHHHHSS??STHHHHHHHHHHHHHHHSS-?G-GGHHHHHHHHHHHHHHHHHH??S?S?SSH-------HHHHH--HHHHHHHHHHHHHHHHHHH-----------------S?HHHHHHHHHHHHH-HHHHHHHHHHHHH--HHHHH  &nbspPDB/DSSP  
MODEL    KLSSTKKVVATAILAALFVVLGRAFPFINIPTPIPNTGGVYINLGDAGIALAAVLFGPKVGFLVGGIGHALKDLLSGVYGSIWAPFTLIIKGLEGLIAGLIAKKLKKNLESHDKNGIFNLLGTFLLLFNIVMVIGYFIAWGLLNPAAAPIGDILIYQPSEPAWKVALQSIPGNIVGQAVVGAIVGLPLLTYKALAK  &nbspHMMER2[PF07155.7\_ECF-ribofla\_trS\_seed]  
QUERY    GL----------IAASLAHALAL-F--VAVSVGANISGG-HVNPAVTF------------GAFVGGNISLLKAL-------VYWVAQLLGS----VVACLLLKIATGGAA---------LGA--FSLSAGVGAMNAVVLEMVM----TFGLVYTVY----ATAVDPKKGDLGVIA-PIAIGFIVGANIL-----AG   Q9ATM0|Aquaporin|TIP12\_MAIZE[57 190]  
  
  
**PF06912.6\_DUF1275\_seed:**  

**E:1.20e-02[Original]  &nbspE:7.57e-04[Fold-critical]  &nbspE:1.61e-05[Remnant]  &nbspRatio[FC/R]:4.70e+01  &nbspClassification:TP**

TM       -----ccccccccccccccccccccccccc----------------------cccccccccccccccccccccccccc----------------------cccccccccccccccccccccccccc----------------------cccccccccccccccccccccc---------------------------------------------------------------------ccccccccccccccc-ccccccccccccccccccccccccccccccccc---  &nbspTMSOC  
STRUCT   CHHHHHHH1HHHHHHHHHHHHHH1CCHHHHH1HHHHHHHHHHHHH11CCCHHHHHHH1111HHHHHHHHHHHHHHHHH1111111111HHHHHHHCCHHHHHHHHHHHHHHHHHHHH111H------------------------------1HHHHHHHHHHHHHHHHHHH11HHCCCCC1CHHHHHHHHH1111HHHHHHH111HHH1111111-----------------111HHHHHHHHHHHHHHHHHHH-HHHHHHHHHHH1H11HHHHHHHHHHHHHHH1HC11C  &nbspPredictedSECSTR  
MODEL    LRLALLLAGFVAGFVDAYGFLALKGGVFASHPMTGNVVLLGVALAGKGGNWALALHLWAKSLLALLAFVLGAALAGLLLRHLGGARVRINFGRRRRLRRWYALLLLLEAVLLLAAGLALLLPAALPPPGTPEDPLFESGSIDTLTGGDALAVLLAILLLAFAMGLQNAAFRSSKLSGAEIGRTTMMTGNLTLVMSDLGIDLGLFARLLRGKAKRDRAEPRERRRTQALWAPPVRADRRRLLLYLLIILGFLLGA-VLGALLFRHLGSFMAWALWLPAALLLILALWLYLSL  &nbspHMMER2[PF06912.6\_DUF1275\_seed]  
QUERY    GLIAASLA------------HAL--ALFVAV-------SVGANIS--GGHVNPAVTF-------------GAFVGGNI-------------SLLKALVYWVAQLLGSVVACLLLKIA---TGGAA----------------------------LGAFSLSAGVGAMNAVVL------EMV--------MTF----GLVYTVY---A--------TA----------------VDPKKGDLGVIAPIAIGFIVGANILAGGAFDGAS--MNPAVSFGPAVVTGV--WEN--H   Q9ATM0|Aquaporin|TIP12\_MAIZE[57 216]  
  
  
**PF01384.15\_PHO4\_seed:**  

**E:9.35e-03[Original]  &nbspE:2.97e-06[Fold-critical]  &nbspE:2.99e-09[Remnant]  &nbspRatio[FC/R]:9.93e+02  &nbspClassification:TP**

TM       ---------------------cccccccccccccccccccccccc--ccccccc-----------------------------------cc--------------------------cccccccccccccccccccccccc----cc----cccccccccccccccccccccccc------------------------cc---cccc-------------cccccccccccccccccccc-------cccccccccccccccccccccccccccc---ccc------------------------------------------------------------cccccccccccccccc-------------------ccccccccccccccccc-------------------------------------------------------------------------------------------------------------------------------------------------------------------------------------------------------------------------------------------------------------------------------------------------------------cccccccccccccccccccc-----ccccccccccc---------------------------------------------------------------------------------------------------------------------------------------------------------------------------------cc-------------------ccc------ccccccccccccccccc-------------------ccccccccccccccccc---------ccc------ccccccccccccccccc--------------------------------------------------------------------------------------------------------------cccccccc----------------------------------------------ccccccc-----cccc-  &nbspTMSOC  
STRUCT   CCCCCCCHHHHHHHHCCCCCHHH1HHHHHHHHHHHH1111HHHH11111HHHHHHHHH1111--------------------------HHH-------------------------1HHHHHHHHHHHHHHHHHHHHHHCCCCHHHH----HHHHHHHHHHHHHH------------------1111111111111111CC111CCHH-----------1111111111111111111111111111HHHHH111HHHHHHHHHHHHH1HHHHHHH111HHHHHHHHHCCC11111HHHH111-----------------------------------HHHHHH1111111111111------------1111111111111111111111111111111---------------------------------------------------------------------------------------------------------------------------------------------------------------------------------------------------------------------------------------------------------111------------------111111111111111HHHHH1111111HHHHHHHHHHHHHHHHCCHHHHHHHHHHHHHHH111111HHH--------------11111111111111------------------------------------------------------1111111111111111111111------------------------111111111111111111111111111111111111111CCHH-----------------H11HH111111HHHHHHHHHHHHHHHHHHHHHHHHCCCCC11CCCHHHHHHHHHHHHHHHHHHH111111111111111111111HHHHHHHHHHHHHHH--------------11----------------------------------------------------------------------------------------H111HHHHHHHHH----------------------------------------------HHHHHHH11111HHHCC  &nbspPredictedSECSTR  
MODEL    IGANDVANAFATAVGSRALTPRQVAVIIAAIFEFLGSVASAVLATYILAGGAVAKTIGKLGIVDPSLFTATAQYNATTNQYDTVDANDPAVLVGGWKSPDIMPGAPPVECAKYTSILLMLGMLAALLAAALWLLIATYLGLPVSTTH----AIVGGLIGAGLAAGLMNTGGSLVFSAVIWPSLSMILGVILYGLLGAVIGALSMVNWGWKADILKDPVKLALLLGAGFIGAVLAIPLAIIFGEFPFSGLVGKLLYIVASWIISPLLGGLLLAALLFRGLLLLRRLVLRRKNSKKPLKRALTLDRIELGALLIITIVFVYYQMIVYYLIDYPVVRSGAIRPVPFLVAVTAGIFSFFIVLKGLKSLLLDSLPLAAIAAAIVGAGIVVALLVLLFLLPYRRRKIKKRLKTRKAEHEIYGPSLWRREPPPPPPRGALRNKDYRYYDGFETASESATESVESQEDRKQPGAADTANAKESNQGGENKKPKNLTKDVAPVVTLDSLSLDRRNDGAADHDSSESSFTYGCPTHSRSKTSTDLVDLTNLVSSSISNSKSKKGDKNGKDSLANSDLSGTSPEGKSADSDEEDAGTDNEKDASDASSSSISGSSSKESSESEEDSRSAAGSNEKASTLEAKAKRLSLVSKASALLEAKSALKSGLLIGLAAALLGILVLRPDVRRVHARAENFDNRVEKLKPRLPFWFRRLQILSAALMSFAHGANDVANAIGPLAAILIGTYAPAIYSTGITGSVSSSKEIDRTRDAIANFEEYYQRNYELLKTILIENVLEAKLLEPLKPLKVNSASLNSSKLRFSDIIENIARNLLLSSSELKGAYASIKIQLRVDLRILRSALLLLENLDNYAQLNIDQRSYNRRYLLSISDFLAEKAALPEVSAKDKNFLKKLRKDLLHTTSVVPSWLAFMHPDAHAGDGIALGYWVHTGGRALLLGGLAIALGLLTGGYRVIKTVGKKITKRELTPSRGFSAELGAAITVLLASLQELSNFVVVLGLPPIPLIPVSTTHVIVGAIVGVGLAREWLKRKYERSLQYIREKSIELDPKNEKEFLDRFKKAGQRNLDSANAEDTVSTSQESDKKDESVSELKRIVKLLKKDADESKITKKERKNIKKRGGTLKTGAVNWINAGVVRKIVLAEKPEMSVNALTAETDTSVPDVGEESSQELTNQTLFDPGTTGRVIFDWILTLPAVGFSTAALLS  &nbspHMMER2[PF01384.15\_PHO4\_seed]  
QUERY    -------------------TAKA------AVAEFIS----TLIF--------VFAGSGS-GMAFSKLTD-----------------GGAAT---------------------------PAGLIAASLAHALALFVAVSVGANISGGHVNPAVTFGAFVGGNISLL----------------------------------KA---LVYW-----------------------------------------VAQ------------LLGS-VVACLLL---KIATGGA--------ALGAFS----------LSAG--------------------VGAMNAVV--------------------------------------------------------------------------------------------------------------------------------------------------------------------------------------------------------------------------------------------------------------------------------------------------------------------------------------------------------LEMV-------MTFGLV-------------------------YT------VYA---TA-VDPKK------------------------------------------------------------------------------------------------------------------------------------------------------------GD-------------------L--GV------IAPIAIGFIVGA--------NILAGGAFD--------------------GASM----NPAVSFGP---------------------AVVT----------------------------------------------------------------------------------------------G-VW--ENHW----------VY----------------------------------------------WVGPLAG-----AAIAA   Q9ATM0|Aquaporin|TIP12\_MAIZE[19 231]  
  
  
**PF09490.5\_CbtA\_seed:**  

**E:6.99e-02[Original]  &nbspE:7.32e-05[Fold-critical]  &nbspE:8.62e-09[Remnant]  &nbspRatio[FC/R]:8.49e+03  &nbspClassification:TP**

TM       ----ccccccc--cccccccccccccccc-----------------------------------------------------------------------------------------------------------ccc----cccccccccccccccccccccc-------------ccccccccccccccccccccc-------------------------------------------ccccccccccccc-----cccccccccccccccccccccccccccc--------------------------cccccccccccccccccccccccc--  &nbspTMSOC  
STRUCT   CHHHHHHHHHH--HHHHHHHHHHHHHHHHHHHHHHHHHHH1--------------------------------------------------------------------------------------------1HHHHH1111HHHHHHHHHHHH1HHHHHHHHHH-----------HHHHHHHHHHHHHHH11-------------1H-----------------------------HHHHHHHHHHHHHHHHH-----HHHHHHHHHHHHHHHHHHHHHHHHHH-----------------------HHHHHHHHHHHHHHHHHHHHHHHHHHHHHCC  &nbspPredictedSECSTR  
MODEL    MFRRILTSALF--AGLLAGLLATLLQLLFVTPLILEAETYESAGEAAPAAPSRDETSFAPVIVEVHFHAEAGATHEHAASGTAMSSAPPAANSMSAADHAGAAAAAHEHEHDAAEHDHAEAEWAPADGLEEPVSRTLLTTLGLVLANVLVAVGFALLLLAALMALAEGRGGAKGTARQGLLWGLAGFAAFHLAPALGLPPELPGMAAADTITRRTVLYFLMLLISLIVVVLAFVIARLLAARQIWWLATVAATAL-----GLALIAFGRSWWLKALALVLLVAPHVIGAPQVDAPPLAAPESHAGPAPAELAAQFAAASLGTSAVFWAVLGLLAGWFWQR  &nbspHMMER2[PF09490.5\_CbtA\_seed]  
QUERY    -FSKLTDGGAATPAGLIAASLAHALALFVAVSV--------GANI----------------------------------------------------------SGGHV--N-------------PAVTFGA-FVGGNIS----LLKALVYWV--AQLLGSVVACLL-LKI-ATGGAALG---------------AFSLSA---GVGAMN------------------AVVLEMVMTFGLVY-------TVYATAVDPKKGDLGVI-------------APIAIGFIVGANILAG---GAFDGASMNPAVSFGPAVVTGVWENHWVYWV--GPLAGAAIAA   Q9ATM0|Aquaporin|TIP12\_MAIZE[44 231]  
  
  
**PF04211.8\_MtrC\_seed:**  

**E:6.32e-02[Original]  &nbspE:5.91e-04[Fold-critical]  &nbspE:5.46e-09[Remnant]  &nbspRatio[FC/R]:1.08e+05  &nbspClassification:TP**

STRUCT   111111111------11111111111111111111111111111111-----11111111111111-1111111---111111111111111111111111111111--111111111111----111111111111111111------111111--11111111111111111111111111111111-11111111111---1111111111111111111111111111111111111111111111111111111111111111111111111111111111111111111111111111111111111111  &nbspPredictedSECSTR  
MODEL    MSAVAAGGE------AEEVEVAVAIPEEKLMALGIIGGLVGIYLANF-----APPYGVGPLIGGLG-AICAVVW---GADAVRRVASYGLGTGVPSIGMMSLGMGIL--AAVAGLAVSGVF----TLPALAAPIVGLVVAAVI------GAVVGV--LAKKVVKMKIPIMERCMTEISAAGALALLGLS-VAIAGSFTFQA---VVSYVIATGLIALLFIIAGMAILHPFNACLGPNEKQERTLKLAVECGFIALVVAGFASSLHEGLLKAGLVPGWWLVLLVGIILWLVAFVKFVELSKRDAAAVVWSGLLPKEE  &nbspHMMER2[PF04211.8\_MtrC\_seed]  
QUERY    IAV-GAPGELSHPDTAKA---AVAEFISTLIFV-FAGSGSGMAFSKLTDGGAATP--AGLIAASLAHALALFVAVSVGAN---------------------ISGGHVNPAVTFGAFVGGNISLLKALVYWVAQLLGSVVACLLLKIATGGAALGAFSLSAGVGAMNAVVLEMVMTF----------GLVYTVYATAVDPKKGDLGVIAPIAIGFIVGANILAGGAFD---GASMNP----------AVSFG--PAVVT--------GVWENHWV--YWVGPLAGAAIAALVY------------DIIFIGQRPHQQ   Q9ATM0|Aquaporin|TIP12\_MAIZE[6 246]  
  
  
**PF02028.12\_BCCT\_seed:**  

**E:7.24e-02[Original]  &nbspE:1.05e-07[Fold-critical]  &nbspE:2.31e-19[Remnant]  &nbspRatio[FC/R]:4.55e+11  &nbspClassification:TP**

STRUCT   HHHHHHHHHHHHHHHHH-----SS-SHHHHHHHHHTTHHHHHHTHHHHHHHHHHHHHHHHHHHHSG-GGG??S-SSTT???SS?HHHHHHHHHHHH??HHHHHHHHHHHHHHHHT?----------------?--TTS?-----------SS?H-HHHHHHHHHHHSHHHHHHH-HHHHHHHHHHTTT-S???----------------SSGGGGG-TTTS?-T-TT-??SS----SSHHHHHHHHHHHHHHHHHHHHHHHHHHHHHHTTTS-?-------?--??-?------??HH-HHHHHHHHHHHTTGGGTTS??S--HHHHHHHHHHHHHHHHHHHHHHHS?-HHHHHHHHHHHHHHHHHHH------------HHHHT??---TT---TT------SS?H----------------------------------------------HHHHHTTHHHHHHHHHTTHHHHHHHHHHHTTT??HHHHHHHHHHHHHHHHHHHHHHHHHHHH---------HHHH-----HT?--????--------------TT?S-HHHHHHHHHTS-------------TTHHHH----HHHHHHHHHHHHHHHHHHHHHHHHHHTTTT---?S--S??HHHHHHHHHHHHHHHHH-HHHH--HGG----GHHHHHHHHHHHHHHHHHHHHHHHHHHH---------------------HHHHHT?  &nbspPDB/DSSP  
MODEL    VFIISALIILLFVLWGILPESLIFDPEAAGAVFNTLFAWITNNFGWFYLLLVLFFLVFLLFLAFSRKYGNIRLVGGDDEKPEFSTFSWFAMLFSAGMGIGLVFWGVAEPLYHFLSPFKLLGTAENTNYIAGTPDIPGGGAELGIEPGTIGPEAAAREAMAYTFFHWGLHAWAIYGALVGLALAYFAYRRKGLPGEKDEEDEEDEEDKGELLISSALRYPLLGDEKKRRIYGPASRVIGKAIDILAVFATVFGVATSLGLGVLQINAGLSYLFPGADDDDDWIMDPPENAGSGSLSLTVAQLIIIAIITALATISAVSGLDKEVGIKRLSNLNMVLALLLLLFVLIVGPMTLFILNTFVQSLGDYLQNFLSISFGAYGPQDVRMSFRTGAADPLGRFGGLGTDGDDGAGDALRGGATNAWGSFEGFKSGLEGEAAALSDEVLAAAYAAGEPGALAEWLGGWTIFYWAWWIAWAPFVGMFIARISRGRTIREFVLGVLLVPTLFTFLWFSVFGGTAILAEQLDRQTDLELSASWNNGGGGDLADDKAKYAAGTVLVLVAADVVEAALFALLEQLLFRLNLGGADMSGPLGTILALLASVLAIVLIIIFFVTSADSATLVLAMLTSGGLDSDLDLNPPRWQRVFWGVLLGAVAAVLLLLAGEGGTKGDADALDALQTASIIAALPFSVILLLMCISLDFLQIQWVGALVIILYFCCFTLKALREE  &nbspHMMER2[PF02028.12\_BCCT\_seed]  
QUERY    IS------TLIFVFAGS-----GS------------------------------------GMAFSK------L--TDGGAA---------------------------------T-----------------P--AGL-------------IAA-SLA----------HALALF---VAVSVG----------------------------------ANISG-G--H-VNPA----------------VTFG----------------AFVG-G-----------N-I------S-LL-KALVYWVAQLLGSVVACLLL-K------------IATGGAALGAFSLSA-GVGAMNAVVLEMVMTFGLV--------------YTVYA---TA---VD------PKKGDL-------------------------------------------------------------------------------------GVIAPIAIGF----------I------------------VGAN-ILAG-------------GAFDG---------ASMN------------PAVSFG----PAVVT------------------------GVWENH-----------------------WVYW--VG--------------------PLAGAAIAALVYD---------------------IIFIGQR   Q9ATM0|Aquaporin|TIP12\_MAIZE[28 242]  
  
  
**PF13303.1\_PTS\_EIIC\_2\_seed:**  

**E:2.48e-02[Original]  &nbspE:7.21e-04[Fold-critical]  &nbspE:1.16e-11[Remnant]  &nbspRatio[FC/R]:6.22e+07  &nbspClassification:TP**

STRUCT   CCCHHHHHHHHHHHHHHHHHHHHHHHHHCCC1111111111H-------------------HHHHHHHHHHHHHHHHHHHHHHHHCCCCHHHHHHHHHHHHHHCCCEEE---------------------------EECCCCHHHHHHHHHHHHHHHHHH11111111111EEEHHHHHHHHHHHHHHH1HHHHHHHHHHHHHHHHHHHHHHCHHHHHHHHHHHHHHHHHCH1HHHHHHHHHHCCCC---------------------HHHHHHHHHHHHHHH11HHHHHHHCCCCCCCHHHHHHCCCCCCCHHHHHCCH1HHHHHHHHHHHHHHHHHHH1--------------------------HHHHHHHHH11------------------H11HH1HHHHHHHHHHHHHHHHHHHHHHH------------------------HHHH11111111111  &nbspPredictedSECSTR  
MODEL    IKVLNGMALGIFVTLIPGAILGTIGKLLGILFPIFALALVSSTGELAAKVAGLSMLNRFCWFLVQIGTLAQSLLGPAIGVAVAYQLKANPLVSFSAGIAGFIGSGAVKFVQTASVATATKTATGGVNATEGGVVDPLAGTGDPINAFIAAAIAVLVGKLISKNLGGKTKLDIILVPIVVILVGGLIGLLVILPYVKPITTAIGNVINSATDLQPLLMGILIAVIFGILITSPFISSAAIAIALGLTGFGVGNALAIPDPIAIVWNGLALAAGAAAIGCCAQMVMNGLAVASLKVNGLGGLIAQGLGTPKLQMPNILKNPIPILLPPLLAAAILGPIATLLFDGYQWFIGIQPNGTPASAVWAGFGTSGLVGPIAALNLMAVNGTVRDVADKMAGTGPSAGTVILLIILVFFVLPAVLALLIYKLFKGFAKKDYKLDEIPTNEINEKNDDIKKLGLIKPGDTLKL  &nbspHMMER2[PF13303.1\_PTS\_EIIC\_2\_seed]  
QUERY    ------TAKAAVA-----EFISTLIFVFAGSGSG-------M-------------------AFSKLTDGGAATPAGLIAASLAHAL---ALFVAVSVGA----------------------------NISGGHV-------NP-----AVTFGAFVGGNIS----LLKAL------------VYWVAQL-LGSVVACLLLKIATGGAALGAFS-------------------L--SAGVGAM-----NAVV-----------------LEM------VMTFGL--VYTVYATAVDPKKGDLGVIA---------------PIAIGFIVGANILAGGAFDGA-------SMN----PAVS---------------------------------FGPAV--VTGVWENHWVYWVGPLAGAAIAALVY------------------------DIIFIGQRPHQ-QLP   Q9ATM0|Aquaporin|TIP12\_MAIZE[19 248]

---

**User sequence**  

Q9ATM0|Aquaporin|TIP12\_MAIZE  
MPVSRIAVGAPGELSHPDTAKAAVAEFISTLIFVFAGSGSGMAFSKLTDGGAATPAGLIAASLAHALALFVAVSVGANISG  
GGHVNPAVTFGAFVGGNISLLKALVYWVAQLLGSVVACLLLKIATGGAALGAFSLSAGVGAMNAVVLEMVMTFGLVYTVYA  
ATAVDPKKGDLGVIAPIAIGFIVGANILAGGAFDGASMNPAVSFGPAVVTGVWENHWVYWVGPLAGAAIAALVYDIIFIGQ  
QRPHQQLPTTAADY

---

**Main reference**  

1) Wing-Cheong Wong, Sebastian Maurer-Stroh, Birgit Eisenhaber, Frank Eisenhaber, 2014,
*The necessity of dissecting similarity scores for inferring homology: an essential issue in protein function prediction and annotation*
, BMC Bioinformatics, 15(1):166, doi:10.1186/1471-2105-15-166

**Relevant references**  

1) Wing-Cheong Wong, Sebastian Maurer-Stroh, Georg Schneider, Frank Eisenhaber, 2012,
*Transmembrane helix: simple or complex*
, Nucleic Acids Research (Web Server issue), doi:10.1093/nar/gks379  
  
2) Wing-Cheong Wong, Sebastian Maurer-Stroh, Frank Eisenhaber, 2011,
*Not all transmembrane helices are born equal: Towards the extension of the sequence homology concept to membrane proteins*
, Biology Direct, 6(57), doi:10.1186/1745-6150-6-57  
  
3) Wing-Cheong Wong, Sebastian Maurer-Stroh, Frank Eisenhaber, 2011,
*The Janus-faced E-values of HMMER2: Extreme value distribution or logistic function*
, Journal of Bioinformatics and Computational Biology, 9(1), doi:10.1142/S0219720011005264  
  
4) Wing-Cheong Wong, Sebastian Maurer-Stroh, Frank Eisenhaber, 2010,
*More than 1001 problems with protein domain databases: transmembrane regions, signalpeptides and the issue of sequence homology*
, PLoS Computational Biology, 6(7), doi:10.1371/journal.pcbi.1000867

**Contact**  

wongwc@bii.a-star.edu.sg

  

24-03-2015 17:06:31 Copyrights Bioinformatics Institute A\*STAR

  
