# Supplementary material for: dissectHMMER: a HMMER-based score dissection framework that statistically evaluates fold-critical sequence segments for domain fold similarity
Source: Biol Direct. 2015 Aug 1;10:39. doi: 10.1186/s13062-015-0068-3 (PMC4521371; doi:10.1186/s13062-015-0068-3)
Supplement: Additional file 8: — The web output of dissectHMMER search results for the protein sequence Q9K8K1_BACHD. (ZIP 19 kb) [file 13062_2015_68_MOESM8_ESM.zip › 5864776041655568_add8.html]

  
  

dissectHMMER


# dissectHMMER results

---

**Overview of Sequence Annotation**  

  
Domain(s) to sequence segment[29,379]  
Domain name?                     &nbspPDB?    &nbspLength?  &nbspSeq range?  &nbspScore[FC/R]?  &nbspFPR[FC/R]?  &nbspScore[FC]?  &nbspFPR[FC]?  &nbspDomaincoverage?   **Total FPR?**   **Hits'classification?**     
PF05684.7\_DUF819\_seed         ?  &nbsp-       &nbsp400      &nbsp10,388      &nbsp1             &nbsp0.00        &nbsp1           &nbsp0.00      &nbsp1                 **0**   **TP,TP**  
PF07690.11\_MFS\_1\_seed         ?  &nbsp2CFP|A  &nbsp793      &nbsp13,382      &nbsp0.845         &nbsp0.00        &nbsp0.815       &nbsp0.00      &nbsp1                 **0**   **TP,TP**  
PF03706.8\_UPF0104\_seed        ?  &nbsp-       &nbsp626      &nbsp97,383      &nbsp0.615         &nbsp0.01        &nbsp0.63        &nbsp0.00      &nbsp1                 **0.01**   **TP,FN**  
PF00375.13\_SDF\_seed           ?  &nbsp3V8G|A  &nbsp618      &nbsp7,388       &nbsp0.45          &nbsp0.09        &nbsp0.74        &nbsp0.00      &nbsp1                 **0.09**   **TP,TP**  
PF00999.16\_Na\_H\_Exchanger\_seed?  &nbsp4BWZ|A  &nbsp593      &nbsp10,387      &nbsp0.415         &nbsp0.15        &nbsp0.49        &nbsp0.03      &nbsp1                 **0.18**   **TP**  
PF13347.1\_MFS\_2\_seed          ?  &nbsp4LDS|A  &nbsp847      &nbsp36,382      &nbsp0.415         &nbsp0.15        &nbsp0.49        &nbsp0.03      &nbsp1                 **0.18**   **TP**  
PF07155.7\_ECF-ribofla\_trS\_seed?  &nbsp4HZU|S  &nbsp196      &nbsp251,387     &nbsp0.375         &nbsp0.23        &nbsp0.485       &nbsp0.04      &nbsp1                 **0.27**   **TP**  
PF03547.13\_Mem\_trans\_seed     ?  &nbsp-       &nbsp746      &nbsp29,379      &nbsp0.36          &nbsp0.25        &nbsp0.485       &nbsp0.04      &nbsp1                 **0.29**   **TP**  
PF07884.9\_VKOR\_seed           ?  &nbsp3KP9|A  &nbsp174      &nbsp250,379     &nbsp0.395         &nbsp0.18        &nbsp0.435       &nbsp0.13      &nbsp1                 **0.31**   **TP**  
PF00083.19\_Sugar\_tr\_seed      ?  &nbsp4GC0|A  &nbsp605      &nbsp6,385       &nbsp0.225         &nbsp0.50        &nbsp0.485       &nbsp0.04      &nbsp1                 **0.54**   **TP**  
PF00115.15\_COX1\_seed          ?  &nbsp1V55|A  &nbsp591      &nbsp22,381      &nbsp0.12          &nbsp0.70        &nbsp0.475       &nbsp0.06      &nbsp1                 **0.76**   **TP**  
PF09847.4\_DUF2074\_seed        ?  &nbsp-       &nbsp504      &nbsp1,388       &nbsp0.1           &nbsp0.75        &nbsp0.42        &nbsp0.15      &nbsp1                 **0.9**   **TP**  
PF07556.6\_DUF1538\_seed        ?  &nbsp-       &nbsp262      &nbsp222,377     &nbsp0.07          &nbsp0.83        &nbsp0.45        &nbsp0.10      &nbsp1                 **0.93**   **TP**  
PF03611.9\_EIIC-GAT\_seed       ?  &nbsp-       &nbsp642      &nbsp7,379       &nbsp0.035         &nbsp0.90        &nbsp0.485       &nbsp0.04      &nbsp1                 **0.94**   **TP**  
PF03169.10\_OPT\_seed           ?  &nbsp-       &nbsp1010     &nbsp3,388       &nbsp0.05          &nbsp0.88        &nbsp0.45        &nbsp0.10      &nbsp1                 **0.98**   **TP**  
PF02028.12\_BCCT\_seed          ?  &nbsp2WSW|A  &nbsp722      &nbsp7,330       &nbsp0.005         &nbsp1.00        &nbsp0.5         &nbsp0.01      &nbsp1                 **1.01**   **TP**  
PF13303.1\_PTS\_EIIC\_2\_seed     ?  &nbsp-       &nbsp464      &nbsp195,387     &nbsp0.035         &nbsp0.90        &nbsp0.435       &nbsp0.13      &nbsp1                 **1.03**   **TP**  
PF03806.8\_ABG\_transport\_seed  ?  &nbsp-       &nbsp518      &nbsp25,386      &nbsp0             &nbsp1.00        &nbsp0.475       &nbsp0.06      &nbsp1                 **1.06**   **TP**  
PF01654.12\_Bac\_Ubq\_Cox\_seed   ?  &nbsp-       &nbsp809      &nbsp6,325       &nbsp0.005         &nbsp1.00        &nbsp0.44        &nbsp0.11      &nbsp1                 **1.11**   **TP**  
PF01970.11\_TctA\_seed          ?  &nbsp-       &nbsp570      &nbsp97,385      &nbsp0.015         &nbsp0.98        &nbsp0.435       &nbsp0.13      &nbsp1                 **1.11**   **TP**  
PF11299.3\_DUF3100\_seed        ?  &nbsp-       &nbsp252      &nbsp230,385     &nbsp0.005         &nbsp1.00        &nbsp0.445       &nbsp0.11      &nbsp1                 **1.11**   **TP**

---

**Alignments**  
  
**PF05684.7\_DUF819\_seed:**  

**E:5.30e-244[Original]  &nbspE:4.37e-35[Fold-critical]  &nbspE:5.72e-15[Remnant]  &nbspRatio[FC/R]:7.64e-21  &nbspClassification:TP**

TM       -------------------------cccccccccccccccccccccc----------------------------------------ccccccccccccccccccccccc------------------------------------------cccccccccccccccccccccccc----------------------------------------ccccccccccccccccccc----------------------cccccccccccccccccc---------------cccccccccccccccccccc-----cccccccccccccccccccccc-------cccccccccccccccccccc------cccccccccccccccccccccccc------  &nbspTMSOC  
STRUCT   1111111111111111111111H11HHHHHHHHHHHHHHHHH--------------HHHHHHHHHHHHHHHHHHHHHHHHHHHHHHHHHHHHHHHHHHHHHHHHHHHHHH-----------HHHHHHHHHHCCCCCHHHHHHHHHHHCCCHHHHHHHHHHHHHHHHHHHHHHHHH-----------------------------HHHHCCCCH1HHHHHHHHHHHHHHHHHHHHHHHH------------------------HHHHHHHHH---------------HHHHHHHHHHHHHHCCCCCHHHHHH1HHHHHHHHHHHHHHHHHHHHHHHHHHHHHHHHHHHHHHCCCCCCCHHHHHHHHCCCCCCCHHHHHHHHHHHHHHHHHHHHHHHEEHCC  &nbspPredictedSECSTR  
MODEL    GFLAATAALGLWLSAREEKTKWKFFSVVPAALLIYLLAMFFNTLGLIGDSESESPAYSVVRNFLLPAAIPLLLLRIDLRKIIKLGGKLLLIFLIASVSTVLGFILAFLLMKSFHPASLGPDTWKIAAALAGSWIGGSANMVAMQEALAVPASVFSAALVVDNVIYAVWFAVLFALASLKRSIDAKTKADTSKLKALSEDAAKEEAEEKRNPIALVDIIFLIGVSLAIVAVATVLGEYLKSIFKVNLLTGIQLELDTGGTLTVVTATIVGLLLAFTRFFELLAPGSEEIGTVLLYVFFAVIGSTADLWSILTTAPSIFLFGLIIIAVHLAVLLGLGKLFRVDLFLLLLASNANIGGPTSAPVMATAKGWRSLVPVGVLMGVLGYAIGTFLGILVGVILSKL  &nbspHMMER2[PF05684.7\_DUF819\_seed]  
QUERY    SILIGMTALIVWA---EYKIGGKFFKYVPAIVLIYLGAAFMNTFGLFGDSESLSNTSYGVRNALLPAMILLMLLQCDLRKIIKLGPKLLITYFVAAFSIVLGFTLTYLVMQSF----FLDDTWRAFSALAGSWTGGSANMVALQDILAVPETIFGYALIMDTINYSFWVMVMFWLVPFERMFNRWTKADTSKLESMSQEIAATVTDEKREPTTFVHMIGLLGFSLFIAALATVIGENLP-----QIGTGI-----NAMTWTILIVSIVGLLLALTPFASI--PGSMDIGRVMLYTIVAIIASGADFSSIGE-IPVYIIAGFMVLLFHGLILFGFAKLFKLDLFTLGVASLANIGGMVSAPVLAGAFN-RALIPVGVIMALIGGFMGTWFGVLTAEILSRL   Q9K8K1|Unknown|Q9K8K1\_BACHD[10 388]  
  

**E:3.50e-162[Original]  &nbspE:1.03e-143[Fold-critical]  &nbspE:1.17e-22[Remnant]  &nbspRatio[FC/R]:8.80e-122  &nbspClassification:TP**

TM       -------------------------cccccccccccccccccccccc----------------------------------------ccccccccccccccccccccccc------------------------------------------cccccccccccccccccccccccc----------------------------------------ccccccccccccccccccc----------------------cccccccccccccccccc---------------cccccccccccccccccccc-----cccccccccccccccccccccc-------cccccccccccccccccccc------cccccccccccccccccccccccc------  &nbspTMSOC  
STRUCT   1111111111111111111111H11HHHHHHHHHHHHHHHHH--------------HHHHHHHHHHHHHHHHHHHHHHHHHHHHHHHHHHHHHHHHHHHHHHHHHHHHHH-----------HHHHHHHHHHCCCCCHHHHHHHHHHHCCCHHHHHHHHHHHHHHHHHHHHHHHHH-----------------------------HHHHCCCCH1HHHHHHHHHHHHHHHHHHHHHHHH------------------------HHHHHHHHH---------------HHHHHHHHHHHHHHCCCCCHHHHHH1HHHHHHHHHHHHHHHHHHHHHHHHHHHHHHHHHHHHHHCCCCCCCHHHHHHHHCCCCCCCHHHHHHHHHHHHHHHHHHHHHHHEEHCC  &nbspPredictedSECSTR  
MODEL    ALLAALAALVLWLSAREEKTKVKFFAVVSALLLIYLLALVASTLGLIGDSESESKAYSVVRNVLLPAAILLLLLRIDLRKIIKLGGKLLLIFLIASVSIVLGTVLAFLLLKSLHPASLGPDTWKIAAALAGSYIGGSVNMVALQEALAVDASVLSAALVVDNVIYAVWFAVLLLLASLKRSIDAKTKADTSKLEALAEEAAKEEAEEKRKKIALVDLIVLIGVSLAIVAVAAVLGEYLKSIFKVNLLTGIQLELDSGVTLTVVLATIVGLLLAFTRFFELLAPGAEELGTVLLYVLVAVIGATADLLSILETAPLILLFGLIIIAVHLVVLLGLGKLFRVDLELLLLASNANIGGPTSAPVVATAKGWRSLVAVGVLLGVLGVAIGTFLGILVGEILSKL  &nbspHMMER3[PF05684.7\_DUF819\_seed]  
QUERY    SILIGMTALIVWA---EYKIGGKFFKYVPAIVLIYLGAAFMNTFGLFGDSESLSNTSYGVRNALLPAMILLMLLQCDLRKIIKLGPKLLITYFVAAFSIVLGFTLTYLVMQSF----FLDDTWRAFSALAGSWTGGSANMVALQDILAVPETIFGYALIMDTINYSFWVMVMFWLVPFERMFNRWTKADTSKLESMSQEIAATVTDEKREPTTFVHMIGLLGFSLFIAALATVIGENLP-----QIGTGI-----NAMTWTILIVSIVGLLLALTPFASI--PGSMDIGRVMLYTIVAIIASGADFSSIGE-IPVYIIAGFMVLLFHGLILFGFAKLFKLDLFTLGVASLANIGGMVSAPVLAGAFN-RALIPVGVIMALIGGFMGTWFGVLTAEILSRL   Q9K8K1|Unknown|Q9K8K1\_BACHD[10 388]  
  
  
**PF07690.11\_MFS\_1\_seed:**  

**E:7.72e-02[Original]  &nbspE:4.87e-04[Fold-critical]  &nbspE:6.97e-01[Remnant]  &nbspRatio[FC/R]:6.99e-04  &nbspClassification:TP**

TM       -----------------------------------------------------------------------------------------------------------------------------------------------------------ccc----cccccccccccccccccccccccccccc----------------------cccccccccccccccccccc-----ccccccccccc------cccc------cccccc---ccccccccccccccccccc----------------cc--ccccc---cccccccccccccccc----cccccc--------------cccc----cccccccccccccccccccccccc---------------------------------------------------------------------------------------------------------------------------------------------cccccccccc----cccccccccccccccccc------------------------------------------ccccccccccccccccccccccccccccc----------------------cccccccccccccccccccccc-----------------ccccccccc--------cccccccccccccccccccccccccccccc-----------------------------ccccccccccccccccccc-  &nbspTMSOC  
STRUCT   HHHHHHHHHHHHHH-------------HHHHH-------H--HH--------------HTT------------------------------------------SS----------------------------------------STTHH-----HHH----HHHH---HHH--HH---HHHHTTH-HHH--HT----T-S-----?--III-IIHHHHHHHHHH-HHHHH-----------H---HH------HHHH------HHHHHH---HHHHHH-HHHHHHHHHHHHHHHHT-S-----S??SHH--HHHHH---HHHHHH-HHH-HHHHH----HSS-?HHH------------HHHH----HHHHHHHHHHH---H?--????SS------------------------------???STTT??-------------------------------------------------???TTT?---------STHHHHHH-------HH--------------------HHHHHHHHHH-----HHHHHH----HHHH?S?------S--------SHHHHHHHHH------------------HHHHHHHTHHHHHHHHHH-----HHHTTT-TH-------HHH--------HHHHHHHHHHHTSSSSSHHHHH-------------------HHHHHH-----------HHHHHHHHHHH-HHHHT----SSTTHHHHHTHH----H----------HHHH-----HHHHHHH-HHHH-HHHH??S  &nbspPDB/DSSP  
MODEL    LLLAAFLAALGRSLNVDTLGHGKKVSVLGPALLKMVMPRPLDLLVQKAALSRETELESAEDGNPGSNRSALTNLENMEMTLVNASGSLTDNVENMEHKSADVLLGISVSSLESISYSDESISDNQCYVLDEALDTSLELETTNLAISPTEDVKNYIGLIEAQLLTALISFSLGTGYILAALAQPLALGRLKESDKTETRKFRLGKLGISRRRLVLLLGLLLFALGSLLLLLGLALLFAFLTGAASAASLALAVVLWALEDVLVGLLVLRVLLVLQGLGASGALFPAAAALIADWFPPGEKKEGKERGRALFSGIVSAGNLGFGLGAAALGAPLLGGLIKRLLAASLFGVGIAILTLVLLGWRAASVLYFLILAILALLAALLAVFALALLLLIAEKFGDESVRIDWIGAALVVAGLTKVFPEPRPPPESKRKKPAEELGLLALLSLALVLGLSNNGGKEKAILLVLAAAEGALLGLGEGGAPAPLLPLAVLLALGAWKALLRDPTALAPVLVLLRLRLWKVRRFELTKDPLLALLLLLALLLFKAIFAGFAFFASFGALLTLLPLESADAEYSTDTRLVALQEVLGLSPAGVVVAYLVQLAEAKRGQGLLLAGLLLGLAALLGAIGLVSRLLLGRLSLDRLTIYRGVLGRGGLQEARRRLLLGLLLLLLAALGLALLSLTSSHLNLLTNGVEATEDPESSLWLLVLLEVVELKLVLALLLGAALLGFVGFGLVQSVLFPALNALASDLAAKAAPKYELEHPTFFEERGYKKKTTASGLYNGTAGSNLGGALGP  &nbspHMMER2[PF07690.11\_MFS\_1\_seed]  
QUERY    IGMTALIVWAEYKI-------GGKFFKYVPAI-------VLIYL--------------GAA------------------------------------------FM----------------------------------------NTFGL-----FGDSESLSNTS---YGVRNAL---LPAMILL-MLL--------Q-C-----D--LRK-IIKLGPKLLITY--FVAA-------F---S---IV------LG-F------TLTYLV---MQSFFL-------------------D-----DTWRAF--SALAG---SWTGGS-ANM-VALQD----ILA-----V-----------PETI----FGYALIMDTINYSFWV--MVMFWL----------------------------VPFERMFNRWTKADTSKLESMSQEIAATV--------------------------TDEKREPTTFVHMIGLLGFSLFIAALATV----IGENLPQIGTG------------INAMTWTILIVS-------IVGL----LLALTPF-------------------ASIP-------------------G-SMDIGRVMLYTIVAIIA-----SGADFS-SI---------G------EIPVYIIAGFMVLLFHGLILFGFA---------------KLFKLDLFTL----------GVASL----ANI-GGMVS----APVLAGAFNRAL---------------IPVG-----VIMALIGGFMGT-WFGVLTA   Q9K8K1|Unknown|Q9K8K1\_BACHD[13 382]  
  

**E:9.98e-05[Original]  &nbspE:6.49e-12[Fold-critical]  &nbspE:1.48e+04[Remnant]  &nbspRatio[FC/R]:4.39e-16  &nbspClassification:TP**

TM       -----------------------------------------------------------------------------------------------------------------------------------------------------------ccc----cccccccccccccccccccccccccccc----------------------cccccccccccccccccccc-----ccccccccccc------cccc------cccccc---ccccccccccccccccccc----------------cc--ccccc---cccccccccccccccc----cccccc--------------cccc----cccccccccccccccccccccccc---------------------------------------------------------------------------------------------------------------------------------------------cccccccccc----cccccccccccccccccc------------------------------------------ccccccccccccccccccccccccccccc----------------------cccccccccccccccccccccc-----------------ccccccccc--------cccccccccccccccccccccccccccccc-----------------------------ccccccccccccccccccc-  &nbspTMSOC  
STRUCT   HHHHHHHHHHHHHH-------------HHHHH-------H--HH--------------HTT------------------------------------------SS----------------------------------------STTHH-----HHH----HHHH---HHH--HH---HHHHTTH-HHH--HT----T-S-----?--III-IIHHHHHHHHHH-HHHHH-----------H---HH------HHHH------HHHHHH---HHHHHH-HHHHHHHHHHHHHHHHT-S-----S??SHH--HHHHH---HHHHHH-HHH-HHHHH----HSS-?HHH------------HHHH----HHHHHHHHHHH---H?--????SS------------------------------???STTT??-------------------------------------------------???TTT?---------STHHHHHH-------HH--------------------HHHHHHHHHH-----HHHHHH----HHHH?S?------S--------SHHHHHHHHH------------------HHHHHHHTHHHHHHHHHH-----HHHTTT-TH-------HHH--------HHHHHHHHHHHTSSSSSHHHHH-------------------HHHHHH-----------HHHHHHHHHHH-HHHHT----SSTTHHHHHTHH----H----------HHHH-----HHHHHHH-HHHH-HHHH??S  &nbspPDB/DSSP  
MODEL    xxxxxxxxxxxxxxxxxxxxxxxxxxxxxxxxxxxxxxxxxxxxxxxxxxxxxxxxxxxxxxxxxxxxxxxxxxxxxxxxxxxxxxxxxxxxxxxxxxxxxxxxxxxxxxxxxxxxxxxxxxxxxxxxxxxxxxxxxxxxxxxxxxxxxxxxxxxxxxxxxxxxxxxxxxxxxxxxxxxxxxxxxxxxxxxxxxxxxxxxFRLGKLGISRRRIVLLLGLLLFALGALLLLLILALLVAFLELAASASSLVSKEFLWALTDVLVGLLVLRVLAVLQGLGASGALFPAGAALIADWFPPGEKKEGKERGRAINSGLLSAGNLGFSLGAAILGAPLLGGLMKLLLATSSLGNGISILGLNILGWRAVSVSVFYILAIVSLLALLVFVACLFLLLLLPESHGDESVRIDWIGAALVVAGLCSFMLAPEEPPERKRVSPKEEEKILLALVQILVEGAAINDRSEKIPSDVRQALEQALLGLGKGTGPAPLVPLAVNVGTGAWKALLKDPKALAPVKVLLRLRLWEVREKELTKDKLLALILLIALLLFKVIFAFFGFSGSFLALLTLLPLESIDSAYSTDTRLVFLQEVLGLSPSGVTVAYLEQLQESKRGQGLLLAGLLLALAALVGAILVVMALLAGRLSEDRARCCTPQLGRDQLNEVRRRLLLALLLLILAALGLALLAVTSSHLQLLTNGSEANEDDSSAVLLLVLTEVTEVVLSVVLVLSLAVIGFAGLGFVRSLLFPSLLALASDLAAKAAPPYELEHPTFFEERGMKRETTASGLFNVTAGSxxxxxxxx  &nbspHMMER3[PF07690.11\_MFS\_1\_seed]  
QUERY    xxxxxxxxxxxxxxxxxxxxxxxxxxxxxxxxxxxxxxxxxxxxxxxxxxxxxxxxxxxxxxxxxxxxxxxxxxxxxxxxxxxxxxxxxxxxxxxxxxxxxxxxxxxxxxxxxxxxxxxxxxxxxxxxxxxxxxxxxxxxxxxxxxxxxxxxxxxxxxxxxxxxxxxxxxxxxxxxxxxxxxxxxxxxxxxxxxxxxxxxY-----G--VRN-ALLPAMILLMLL--QCDL------------RKIIK------LG-P-----KLLITYF---VAAFSI-VLGFTLTYLVMQSFFLD-------DTWRAF--SALAG---SWTGGS-ANM-VALQD----ILA--VPE----------TIFGYA----LIMDTINYSFW----V--MVMFWL----------------------------VPFERMFNRWTKADTSKLESMSQEIA---------------------------------------AT---------VTDEKREP-----TTF----------------------VHMIGLLGF-----SLFIAALATVIGENLPQ------I-----------GTGIN------AMTWTIL------IVSIVGLLLALTPFASIPG---SMDIGRVM-LYTIVAIIASGADFSSIGEIPVYIIAGFMVLLFHGLILFGFA---------------KLFKLDLFTL-----------GVAS----LAN-IGGMV----SAPVLAGAFNRA--L------------IPVG-----VIMALIGGFMGTxxxxxxxx   Q9K8K1|Unknown|Q9K8K1\_BACHD[64 375]  
  
  
**PF03706.8\_UPF0104\_seed:**  

**E:5.15e-02[Original]  &nbspE:7.87e-04[Fold-critical]  &nbspE:2.09e-02[Remnant]  &nbspRatio[FC/R]:3.77e-02  &nbspClassification:TP**

TM       -------------------------------------------------------------ccccc--------ccccccccccccccccc------------------------------------------cccccccc---------------------------------------------------c----cccccccc----ccc---cccccccccc-----------------------------------------------------ccc--------cccccccccc----cccccccccccccc---------------------cccc---------------ccccccccccccccccc-------------------------------------------------------------------------------------------ccccccccccccccccccccccccccc--------------cccccccccccccccc---cccc----cccccc--ccc------ccc-----cccccccccc-----ccc-------cccccccccccccccc--ccc-------------------c--  &nbspTMSOC  
STRUCT   CCHHHH111111HHHHHCCHHHHH-----------------------------HHHH111CCCHHH11111111HHHH11HH1111HHHHH------------------------------------------HHH11HHHH-----------HHHHHHH-------------------------111HHHH1H111HHHHHHHH1111HHH111H111111111-----------1HHHHHHHHH---------------1HCCCCH11111HHHHHH1H11111111HHHHHHHHHH1111HHHHHHHHHHHHH1-----------1HHHHHHHHHHHHH---------------HHHHHHHHHHHHHHHHHHH11111111111111111111111111HHHHHHHHHHHHHHHHHHHHH1HHHHHHHH11-------------------111HHCHHHH111HHHHHHHHHHHHHHHHH1HHHHHHHHH-------------HHHHHHHHHHHHHHHH1111H---------------HHHHHHHHHHHH11111HCCCCHHHH11111HHH11111111HHHHHHHHHHHHH111111H--------------------CC  &nbspPredictedSECSTR  
MODEL    SLALLVAFGTELLLLRGLDLSELATEKLAKDSVEDQAVVLESSLDDKKRLVEKAALRTIISANPWWKNIIKFSLLLLAALLLALLGLALLSLFYNFYNSSFSDGSTTRIFEKFYEFFFKPRKNRLVFLGVFGHLLLVLRALRRAYGPGAGRVVWRLLLRAVARSAAKFLGVSEERAHPAKAAAERRLSFRRLFLVLNRAYLIGYFRYAAVNNFQNVGTPAGGRLGGDGIKASYHSYGEVVRAYLLAKGNLTETTELDKELSRREGVPLNEPKLSKALASVVFDTPSRQVVLERLLLLDLAEQGLALLLLALLLLLLLFDDFVTFLLQHPLLLLGLAGLSLLLVGGSIIPSLKKWTEVLLGLLLLALLLLLLLLLRLSPKATRRLIAAAVRLLILALGLLRLLRKRRLLLRLERLRERLARLLERRLREGLRLLFLKTEAGAAGILSVQEAKDVEQLLRSPKRLPRRLLALLLLSLLIWLLEALFALYLLLRALGTLDVNLDQEKPLSLLVVLLALPLATLAGSANGLLPSFFIIITPGGANLGVREAALVLLLANFRPILFGVPAAAAAEGIYLAALDIFIKDTALLWRLITFLLPLLLAGLALAIRHCPRIFNPDRYRSRSNLGL  &nbspHMMER2[PF03706.8\_UPF0104\_seed]  
QUERY    GFTLTY------LVMQSFFLDDTW-----------------------------RAFS---ALAGSW--------TGGS--------ANMVA--------------------------------------------------------------LQDILAV-------------------------PETIFGYAL---IMDTIN------YSF---W-VM--VMFW------------LVPFERMFN-------RWTKADTSKLESMSQ-----EIAATV----------TDEK--REPT----TFVHMIGLLGFSL----FIAALATVIGENLPQIGTGINA---------------MTWTILIVSIVGLLLALTP-----------------------------FASIPGSMDIGRVMLYTIVAIIASGAD------------------------FSSIGEI---PVYI-IAGFMVLLFHGL-ILFGFAKLFK------------LDLFTLGVASLANIGG----MVS------APVL--AGAFNRAL---------------IPVG-----VIM--------ALIGGFMGTWFGV------L-------------------TAE   Q9K8K1|Unknown|Q9K8K1\_BACHD[108 383]  
  

**E:1.07e-03[Original]  &nbspE:6.37e-08[Fold-critical]  &nbspE:1.48e+04[Remnant]  &nbspRatio[FC/R]:4.30e-12  &nbspClassification:FN**

TM       -------------------------------------------------------------ccccc--------ccccccccccccccccc------------------------------------------cccccccc---------------------------------------------------c----cccccccc----ccc---cccccccccc-----------------------------------------------------ccc--------cccccccccc----cccccccccccccc---------------------cccc---------------ccccccccccccccccc-------------------------------------------------------------------------------------------ccccccccccccccccccccccccccc--------------cccccccccccccccc---cccc----cccccc--ccc------ccc-----cccccccccc-----ccc-------cccccccccccccccc--ccc-------------------c--  &nbspTMSOC  
STRUCT   CCHHHH111111HHHHHCCHHHHH-----------------------------HHHH111CCCHHH11111111HHHH11HH1111HHHHH------------------------------------------HHH11HHHH-----------HHHHHHH-------------------------111HHHH1H111HHHHHHHH1111HHH111H111111111-----------1HHHHHHHHH---------------1HCCCCH11111HHHHHH1H11111111HHHHHHHHHH1111HHHHHHHHHHHHH1-----------1HHHHHHHHHHHHH---------------HHHHHHHHHHHHHHHHHHH11111111111111111111111111HHHHHHHHHHHHHHHHHHHHH1HHHHHHHH11-------------------111HHCHHHH111HHHHHHHHHHHHHHHHH1HHHHHHHHH-------------HHHHHHHHHHHHHHHH1111H---------------HHHHHHHHHHHH11111HCCCCHHHH11111HHH11111111HHHHHHHHHHHHH111111H--------------------CC  &nbspPredictedSECSTR  
MODEL    xxxxxxxxxxxxxxxxxxxxxxxxxxxxxxxxxxxxxxxxxxxxxxxxxxxxxxxxxxxxxxxxxxxxxxxxxxxxxxxxxxxxxxxxxxxxxxxxxxxxxxxxxxxxxxxxxxxxxxxxxxxxxxxxxxxxxxxxxxxxxxxxxxxxxxxxxxxxxxxxxxxxxxxxxxxxxxxxxxxxxxxxxxxxxxxxxxxxxxxxxxxxxxxxxxxxxxxxxxxxxxxxxxxxxxxxxxxxxxxxxxxxxxxxxxxxxxxxxxxxxxxxxxxxxxxxxxxxxxxxxxxxxxxxxxxxRLSLLDLAETLLALLLFALLALLLLPEEFVTFLTLFVFLSSELIALLLLVTGGSIIPSLKKWTEVLLLILLAALVLLLILLIRLWKKLQYSLGAAAVRVLIKMLVLLLNLRFFKKLERKKRLEERLARLIDLSFKESLKLVAAKPEAGAAGILSVQEALDVVEILSNKALLPRRLLALLLLSLLIWLLNALLSLYLLLAALGTLDVNLPQIKNLSLTVALVAISLATLASISNNLLPSFFIILTPGGANVGVREAAxxxxxxxxxxxxxxxxxxxxxxxxxxxxxxxxxxxxxxxxxxxxxxxxxxxxxxxxxxxxxxxxxxxxxxxxxxxxxx  &nbspHMMER3[PF03706.8\_UPF0104\_seed]  
QUERY    xxxxxxxxxxxxxxxxxxxxxxxxxxxxxxxxxxxxxxxxxxxxxxxxxxxxxxxxxxxxxxxxxxxxxxxxxxxxxxxxxxxxxxxxxxxxxxxxxxxxxxxxxxxxxxxxxxxxxxxxxxxxxxxxxxxxxxxxxxxxxxxxxxxxxxxxxxxxxxxxxxxxxxxxxxxxxxxxxxxxxxxxxxxxxxxxxxxxxxxxxxxxxxxxxxxxxxxxxxxxxxxxxxxxxxxxxxxxxxxxxxxxxxxxxxxxxxxxxxxxxxxxxxxxxxxxxxxxxxxxxxxxxxxxxxxxTYFVAAF----SIVLGFTLTYLVM----------QSFFLDDTWRAFSALA---------GSWTGGSANMVALQDILAVPETIFG---------YALIMDTINYSFWVMVMFWLVPFERMFNRWTKADTSKLESMSQEIAA-----------------------TVTDEKRE-----PTTFVHMIGLLGFSL-FIAALATVIG-ENL--PQIGTGINAMTWTILIVSIVGL----LLA-----LTPFASIPGSMDIGxxxxxxxxxxxxxxxxxxxxxxxxxxxxxxxxxxxxxxxxxxxxxxxxxxxxxxxxxxxxxxxxxxxxxxxxxxxxxx   Q9K8K1|Unknown|Q9K8K1\_BACHD[97 279]  
  
  
**PF00375.13\_SDF\_seed:**  

**E:8.52e-02[Original]  &nbspE:9.98e-06[Fold-critical]  &nbspE:9.52e-08[Remnant]  &nbspRatio[FC/R]:1.05e+02  &nbspClassification:TP**

TM       -------ccc--ccccccccccccccc--------------------------------------------------ccccccccc----------------ccccccccccccccccc-----------------cccccccccccccccccccccccc------------------------------------------------------------------------------------------------------------------------------------------------------------------------ccccccccccccccccccc---------------------------------ccccccccccccc-------ccccccccccc---------------ccccccccccccccccccccccccccc----------------ccccccccccccccccccc---------------------------cccccccccccccccccccccccccc---ccccc---cccccccccccccccccccccccccccccccccccccc--ccc--cccccccc-ccc---cccccccc----------------------------  &nbspTMSOC  
STRUCT   S----?HHHH--HHHHHHHHHHHHHHTT--------------------------------?--HHHHHHHTH-HHHHHHHHHHHHH----------------HHHH-HHHHHHHHHHSS?---HHHHH---HHHHHHHHHHHHHHHHHHHHH-HHHHHHHT??-----------------------------?S????----------??S?S?????????HH----HHHTTS?S?-------HHHHHH--------TT--------------------------------------------------------------------?HHHHHHHHHHHHHHHHHHHT--?SSHHHHHHHHHHH-HHHHHH--HHHHHHHHHHHHH-HHHHH-------HHHHHHHHHHH-HG-----GG?ST-HHHHHHHHHHHHHHHHHHTHHHHHHH-HTT?-?HHHH-HHHHHHHHHHHHHHT?TTTSHHHHHHHHHHTT----------?--B?HHHHHHHHHHHHHH??HHH-HHHHHHHHHHH---HHHTT??-??-HHHHHHHHHHHHHTTTT????TTHHHHH-HHHHHH--HTT---???TT?HH-HH---HHHHHHHTHHHH-HHHHHHHHHH-HHHHHHHHHHHH  &nbspPDB/DSSP  
MODEL    LLKLTLQILIPFALVLGVVLGLLLQLLLEFPDESLKSEKLPTTIIKKTVDAVPKFGVGSAFVVFPEVATWLKTPLGDLFLNLLKML----------------VVPLIVFFSIVSGIASLGDSEGKKLGDRIRLGGKTLLYFLVTTAIAAIIGALLLANLFQPGAGLSEETRTEPPSA-----IDLRLALSGSGGAAISSAKLSIGRFMAAAASAKATEVPSLLDVNDQFLLNIIPSNDIFPYVHIFAALAGSTVTEAREGYQTKYVPVRKKVMVNNDTENVHSIASVVATSIRILSISSSAYSSTNLAEEAAESTVLWKSSVEYTSGTNVLQVIVFAILFGIALAALGEQNKGPEKLKEKAKPLLKKFFESL--NEVMMKVVNWVMKPLAPIG-------VFALIANTVGTGFGDDERFLGTLGRSLGKYVLTVYLALAIHLFVVYPLLLKFLGGLVNPFKFFLKKIRPALLTAFSTASSAATLPVTLECAEENLRDSSCNDLLDGPVVSKSIASFVLPLGATINMDGTAAIYQAVAAIFILFGAQAYGVDPLSSLGQLLTIILTATLASIGAAGVPGGGLVAALALVLSGIAVGSNLLPVLIAPLDEGAILGIALILAIDRILYDMFRTAVNVTAGDAVAAVVVAKL  &nbspHMMER2[PF00375.13\_SDF\_seed]  
QUERY    L---FLSILI--GMTALIVWA--------------------------------EYKIGGKF--FKYVPAIVLIYLGAAFMNTFGLFGDSESLSNTSYGVRNALLPAMILLMLLQ--CDLRK--IIKLG---PKLLITYFVAAFSIVLGFTLT-YLVMQSFFLD----DTWRAFSALAGSWTGGSANMVALQDILAVPE----------TIFGYALIMDTINYSF----WVMVMF---WLVPFERMFNRWT--------KA----------------DTSKLESMSQEIAATV-------------------------TDEKREPTTFVHMIGLLGFSLFIAALATVIGE--NL--------PQIG-TGINAMTWTILIVSIVGLLLA-LTPFASIPGSMDIGRVMLYTIVA-IIASGADFSSIG-EIPVYIIAGFMVLLFHGLILFGFA--KLFKL-DLFTL-------------------------------------------G--V------------------AS-----------L---ANIGGM--VS------------------------------APVLAG--AFNRALIP-----V-------GVIMALIGGFM-G---TWFGVL-----TAEILSRL   Q9K8K1|Unknown|Q9K8K1\_BACHD[7 388]  
  

**E:9.46e-05[Original]  &nbspE:4.99e-16[Fold-critical]  &nbspE:1.48e+04[Remnant]  &nbspRatio[FC/R]:3.37e-20  &nbspClassification:TP**

TM       -------ccc--ccccccccccccccc--------------------------------------------------cccccccccccccccccccccccccc-----------------cccccccccccccccccccccccc-------------------------------------------------------------------------------------------------------------------------------------------------------------------ccccccccccccccccccc---------------------------------ccccccccccccc-------ccccccccccc---------------ccccccccccccccccccccccccccc----------------ccccccccccccccccccc---------------------------cccccccccccccccccccccccccc---ccccc---cccccccccccccccccccccccccccccccccccccc--ccc--cccccccc-ccc---cccccccc----------------------------  &nbspTMSOC  
STRUCT   S----?HHHH--HHHHHHHHHHHHHHTT--------------------------------?--HHHHHHHTH-HHHHHHHHHHHHHHHHH-HHHHHHHHHHSS?---HHHHH---HHHHHHHHHHHHHHHHHHHHH-HHHHHHHT??------------------------?S????----------??S?S?????????HH----HHHTTS?S?-------HHHHHH--------TT--------------------------------------------------------------------?HHHHHHHHHHHHHHHHHHHT--?SSHHHHHHHHHHH-HHHHHHH--HHHHHHHHHHHH-HHHHH-------HHHHHHHHHHH-HG-----GG?ST-HHHHHHHHHHHHHHHHHHTHHHHHHH-HTT?-?HHHH-HHHHHHHHHHHHHHT?TTTSHHHHHHHHHHTT----------?--B?HHHHHHHHHHHHHH??HHH-HHHHHHHHHHH---HHHTT??-??-HHHHHHHHHHHHHTTTT????TTHHHHH-HHHHHH--HTT---???TT?HH-HH---HHHHHHHTHHHH-HHHHHHHHHH-HHHHHHHHHHHH  &nbspPDB/DSSP  
MODEL    xxxxxxxxxxxxxxxxxxxxxxxxxxxxxxxxxxxxxxxxxxxxxxxxxxxxxxxxxxxxxxxxxxxxxxxxxxxxxxxxxxxxxxxxxxxxxxxxxxxxxxxxxxxxxxxGDRIKLGGKTLLYFLLTTLIAAIIGGLLLALLIKPGAGLSEETKTEPPSAIDAQAAASSSGLALSASAKLSIGRLMASAKSAKKTEVDSLLDVNDILLLNLIPSNDIFPYVHIFEALAGALVTESREGAKTKYVSKEAKVLVVNDTENVHSISSAVLNSIRILSISSSAYSTTTLVKEAAESTVEIKSKAEATSGTNVLAVIVFAILFGIALAALGEKAKGPEKLKEEAKPLLLKFFESLN--EVVMKVVSLVMKPLAPIG-------VFALIAATVASGQGDDEAILGALLTSLGKFLLTVYLGLLIHLLVVLPLLxxxxxxxxxxxxxxxxxxxxxxxxxxxxxxxxxxxxxxxxxxxxxxxxxxxxxxxxxxxxxxxxxxxxxxxxxxxxxxxxxxxxxxxxxxxxxxxxxxxxxxxxxxxxxxxxxxxxxxxxxxxxxxxxxxxxxxxxxxxxxxxxxxxxxxxxxxxxxxxxxxxxxxxxxxxxxxxxxxxxxxxxxxxxxxxxxxxxxxx  &nbspHMMER3[PF00375.13\_SDF\_seed]  
QUERY    xxxxxxxxxxxxxxxxxxxxxxxxxxxxxxxxxxxxxxxxxxxxxxxxxxxxxxxxxxxxxxxxxxxxxxxxxxxxxxxxxxxxxxxxxxxxxxxxxxxxxxxxxxxxxxxI---KLGPKLLITYF-VAAFSIVLG-FTLTYLVMQSFFLDDTW--------RAFSALAGSWTGGSA-NMVALQ--DILAVPETIFGYALIMD----TINYSFWVM-VMFWLVPFERMF--------NR------------WTKADTSKLESMSQEIAATV-------------------------TDEKREPTTFVHMIGLLGFSLFIAALATVIGE--NL--------PQIG-TGINAMTWTILIVSIVGLLLA-LTPFASIPGSMDIGRVMLYTIVAIIA-SGADFSSIG-EIPVYIIAGFMVLLFHGLILFGFAxxxxxxxxxxxxxxxxxxxxxxxxxxxxxxxxxxxxxxxxxxxxxxxxxxxxxxxxxxxxxxxxxxxxxxxxxxxxxxxxxxxxxxxxxxxxxxxxxxxxxxxxxxxxxxxxxxxxxxxxxxxxxxxxxxxxxxxxxxxxxxxxxxxxxxxxxxxxxxxxxxxxxxxxxxxxxxxxxxxxxxxxxxxxxxxxxxxxxxx   Q9K8K1|Unknown|Q9K8K1\_BACHD[88 324]  
  
  
**PF00999.16\_Na\_H\_Exchanger\_seed:**  

**E:8.25e-03[Original]  &nbspE:2.69e-06[Fold-critical]  &nbspE:7.01e-05[Remnant]  &nbspRatio[FC/R]:3.84e-02  &nbspClassification:TP**

TM       -------------------------ccc--------cccccccccccccccccc---------c--------------------------------ccccccccccccccccccccc--------------------------ccccccccccccccc-----------cccccccccc---------------cccccccccccccccccccccccc-----------------ccccccccccccccccccccccc--------------------------------ccccccccccccccccccccc--------------------------cccccccccccccccccccccccccccc-------------ccccccccccccc---------ccc------------------cccccccccccccccccccccc--------------cccc-------------ccccccccccccccccccccccccc------------------------------cccccccccccccccccccccc----------------------------cccccccccccccccccccccccc-------c--  &nbspTMSOC  
STRUCT   HHHHHHHHHHHHH--H-HHTTT---??H--------HHHHHHHHHHHSTTTT?---------?-S???---------------------------HHHHHHHHHHHHHHHHHHHHT??HHH-HHTTHHH--------------HHHHHHHHHHHHHHH------------HHHHHH---------HHT?----?HHHHHHHHHHH-H???HHHHHHHHH-----HTT?TTSHHHHHHHHHHHHHHHHHHHHHHHHHHHH-------HTSS??HHH---------------HHHHHHHHHHHHHHHHHHH-TSTTTS?GGG--------------S??S?HHHHHHHHHHHH--HHHHHHH-TS?H-------------HHHHHHHHHHTT-----------TSHHHHT--?HHH----HHHHH----HHHHHHHHHHHHHT??--------------TTST--------T?SHHHHHHHHHHHHHH-H-HHHHHHHHHTG-----------------G-GHHHHHH---HHH-HTT???HHHHHHHHHHHHHT------T?S?H--------------HH-HHHHHHHHHHHHHHHHHHHHHH-------HHH  &nbspPDB/DSSP  
MODEL    AVLLLLLALLAGLSFLLARRLGSVLLPPEDSLVIQLVVGLILAGILLGPSGLGSTVIYMIDLIPVEPDLHEALKPILEVTLSCEVVEMQFPANVELDLEVLAELGLILLLFLAGLELDLRELLRKNGKSNKKLQNDDEKNLPGILLLALLGVLLPFLLLTILELPGLLGIGLLLALLLFFPMFTTPLGLLLGIPLLEALFLGAALSSATSPVVVLAILKGFAGKERGRLNTRLGTLILGEAVLNDIVAVVLLAVLLALAGVLGLGGGVGGLSDLGLSLEVLRILFLILGILLLLILLVVALGGLLLGLVFPWLLRLLTRFTKRA-------RESGDRELEVLLVLALALLAEGALLAELLFGLSG-------------ILGAFLAGLVLSRLRERKDAAEANSRFRNEKKLSEKSRTTLEPFGYMLAGLFLPLFFVSVGLSLDGILDHIAGEDGGLVLSVLLTDKGDLASNGLSLLLLVLLLLVAILLPGKLLGVFLLARLLNKFSRQRGKKKPRTDGQLSLREALIKWIVGHLGLLQRGEVALALAAIGLQLLNADGNGLIDRITPFKIESKHWQLIELIYTLLVIVVLLTTLVQGLTLKPLIAQIARKLKK  &nbspHMMER2[PF00999.16\_Na\_H\_Exchanger\_seed]  
QUERY    SILIGMTALIVWAEYKIGGKFF-KYVPA--------IVLIYLGAAFMNTFGLF---------------GDS--------------------ESLSNTSYGVRNALLPAMILLMLLQCDLRK-IIKLGPK---------------LLITYFVAAFSIVL------------GFTLT-YLV-----MQSFF-LDDTWRAFSALAGSW-T---------------GGSANM------------VALQDILAVPETIFGYALI-------MDTINYSFW-----------------VMVMFWLVPFERMFNRWTKADTSKLESMSQEIAATVTDEKREPTTFVHMIGLLGFSLFI--AALATVI-GENLPQIGTGINAMTWTILIVSIVGLLLA-----------LTPFAS---IPGS-----MDIG----RVMLYTIVAIIASGAD--------------FSSI----------G--EIPVYIIAGFMVLL-FHGLILFGFAKLF---------------K-LDLFTLG---VAS-LANI-GGMVSAPVLAGAFNR------ALIPV--------------GV-IMALIGGFMGTWF--GVLTAEI-------LSR   Q9K8K1|Unknown|Q9K8K1\_BACHD[10 387]  
  
  
**PF13347.1\_MFS\_2\_seed:**  

**E:8.31e-02[Original]  &nbspE:4.11e-06[Fold-critical]  &nbspE:1.15e-04[Remnant]  &nbspRatio[FC/R]:3.57e-02  &nbspClassification:TP**

STRUCT   ????HHHHH---H-HHHHHHHH--HHHHHH-HHHHHGHHHH--HHHH-------HHHHHHHHHHT---------HHHHHH---------HS?-SHHHHHHHHHHHHHHHHHTT?----------------------------------------------------------------------------------------?SST------------------HHHH--------------------------------------H---HHHHHHH-HHHHHHH--HHH--HHHHHHS?TTS---TTTTHHHHHHHHHH-HH--HHHHHHHTTS----------?SHH---------------------------------------------------------------------------------------------------------------------------------------------------------------------HHHHHHHHHHHHHHH---HHH--------HHHS??S?HHHHHHHHHH?SSS---------------HHHHHHHHHHHHHHHHHHSSS?SSTTHHHHHHHHHH--HHHHHSSSHHHHTHHHHHHHHHTTTST---HHHHTTH-------HHHHHHHHHHHHHH-HH-TTSSS---?SHH-HHHHHHHHHHHHHHHHHHHHH-----TT?HHHHHHHHHHHHHHHHHHHHHTTTTHHHHHTTTTS?---------SS??HHHHHHHHHHHHHHHHHHHH-HGGGG-------GGT------S?SS------------HHHHHHHHHHHHHHH-HHHHT?????  &nbspPDB/DSSP  
MODEL    EKIGGYGLGSILDSLGSNLIYGGGMNLLSTIYLLYFYTDVLFNGLSAGTNNKMVAAVGTIFLVARIWDAFNDPIMGAIIDNLYRTRIRTRWGKKFRPWLLIGAIPLAILLVLLFILPSLMPQLPKMVTVHLQQASNTDIATAGVQKPGLHDSLSWFTVSNRDLTLSGNASSADFSVILHRQPESPTRFTIQTTTDKSAPLHGTTPPFGLSGTGVNWQIPQVVTVKLAYSHGDATNVSMALRILPTSDVLNMPLLNKLGIISWSSLQAVLFYITYLLLFGLAYTFVPLNIPYWSLIPVLTRDPKVGERTSLASYRRIGATLVGGLLLVAVIAPPLVLFRIITDFSKAALGGGNGASTAELSFGIKLKQVILVNEVGNGASRIEATAKLPGYEEPSTFQKILMKVANVPDSKNEGKIYKSKKTYYINNETYFLESKILHKGEQDQLFLDKDLKNAHKAGSDTEPFAQIKYCLEGFKSTSVDMGTVFKKNKDTISDEMVPVDRTLALGNDTLSIARRGYFLAALIIAILAVLTILFLI--------CFLGTKERVERQAGFGIYEDSDGDSATIAAGPIEEVKRVVVPPPPKEKVSLKDMFKALFKNRPLLILLLLYLLDDNALALAVRNGLALYYFTYVLGNAGDGGTGLLFSLFLLEVGGLLTTIGTIAAILGAPLWVPFLLAKRFWVIGKKRRLFLAGLLLAAIGLVLLFFLPPLGLWAEDGTPGNVWLFLVLVVLIGFGLGLATTLLPWAMLADVVDYGEWKNTTGKRREGIVFSVYSFADKLGGALGGAIVGLLLDAFAGYVANATARTSEKAAAADQSAAALSGIRLLFTLIPAVLALLAALILLRFYPLTD  &nbspHMMER2[PF13347.1\_MFS\_2\_seed]  
QUERY    AIVL-IYLG---AAFMNTFGLFGDSESLSN-------TSY---GVRN-------ALLPAMILLMLL-----------QCD-------LRKII-KLGPKLLITYFVAAFSIVLGF-----------------------------------------------------------------------------------------T--------------------------------------------------------------L-----TYLVM----QSFF--------------LDDT---------WRAFSALA-GS-WTGGSANMVAL---------QDILA---------------------------------------------------------------------------------------------------------------------------------------------------------------VPETIFGYALIMDTINYSFWV--MVMFWLVPFERMFNRWTKA----------------------------DTSKLESMSQEIAATVTDEKREPTTFVHMIGLLGFSLF---IAALATVIG---------ENLP---Q---IGTGINA-----MTWTILIVSIVGLLLALTP-FASIPGSMDIGR-VMLYTIVAIIASGADFSSI------------GEIPVYIIAGFMVLLFHGLIL---------------------------FGFAKLFKLDLFTLGVASL-----A-NIGGMVSAP---------V--LAGAFNRAL----IPVGVIMALIGGFMGTWFGVLTA   Q9K8K1|Unknown|Q9K8K1\_BACHD[36 382]  
  
  
**PF07155.7\_ECF-ribofla\_trS\_seed:**  

**E:2.18e-03[Original]  &nbspE:1.21e-05[Fold-critical]  &nbspE:4.27e-05[Remnant]  &nbspRatio[FC/R]:2.83e-01  &nbspClassification:TP**

TM       ------cccccccccccccccccc---ccccc------------cccccccccccccccccccccccccc--------cccccccccccccccccccccccc----------------------ccccccccccccccccccc---ccccc--------------cccccccccccccccccccccccc--ccc--  &nbspTMSOC  
STRUCT   ??-?TTHHHHHHHHHHHHHHHHH-H--???B?S--SS?B-??TTHHHHHHHHHHSS??STHHHHHHHHHHHHHHHSS-?G-GGHHHHHHHHHHHHHHHHHH??S?S?SSH-------HHHHH--HHHHHHHHHHHHHHHHHHH-----------------S?HHHHHHHHHHHHH-HHHHHHHHHHHHH--HHHHH  &nbspPDB/DSSP  
MODEL    KLSSTKKVVATAILAALFVVLGRAFPFINIPTPIPNTGGVYINLGDAGIALAAVLFGPKVGFLVGGIGHALKDLLSGVYGSIWAPFTLIIKGLEGLIAGLIAKKLKKNLESHDKNGIFNLLGTFLLLFNIVMVIGYFIAWGLLNPAAAPIGDILIYQPSEPAWKVALQSIPGNIVGQAVVGAIVGLPLLTYKALAK  &nbspHMMER2[PF07155.7\_ECF-ribofla\_trS\_seed]  
QUERY    TW-TILIVSIVGLLLALT-----PF--ASIPG--------SMDIGRVMLYTIVAI--IASGADFSSIGEIPVYIIAG-FM-----V-LLFHGLILFGFAKLFKLD------------LFTLG----VASLANIGGMVSAPVLA---------------GAFNRALIPVGVIMALI-GGFMGTWFGVLTA--EILSR   Q9K8K1|Unknown|Q9K8K1\_BACHD[251 387]  
  
  
**PF03547.13\_Mem\_trans\_seed:**  

**E:3.11e-03[Original]  &nbspE:6.73e-06[Fold-critical]  &nbspE:1.54e-05[Remnant]  &nbspRatio[FC/R]:4.37e-01  &nbspClassification:TP**

TM       -----ccccccccccccccccccc----------cccccccccccccccccccc----------ccccccccccc---ccccccccccc-----------------ccccc----cccccccccc--cccccccc------------------------cccccccccccccccccccc-----------------------------------------------------------------------------------------------------------------------------------------------------------------------------------------------------------------------------------------------------------------------------------------------------------------------------------------------------------------------------------------------------------ccccccccccc----------ccccccccc----------------------------cccccccccccccccccccccc-----------------------cccccccccccccccccc------------cc------cccccccccccccccccccccc---------ccccccccccccccccccc-  &nbspTMSOC  
STRUCT   CCCCHHHHHHHHHHHH1HHH111111111-1111HHHHHHHHHHHHHHHHHHHHHCCCCCCHHHHHHHHHHHHHH---HHHHHHHHHHH11111HH-----------EEEE----EECCCCCCCC--HHHHHHHH111--------------------111HHHHHHHHHHHHHHHHHH-------------------HHH11111--------------------------------------------------------------------------------------------------------------------------------------------------------------------------------------------------------------------------------------------------------------------------------------------------------------------------------------------------------------11HHHHHHHHHHHHHHCCCHHHHHHHHHH----------HHHH111111---------------------HHHHHHHHHHHCHHHHHHHHHHHH--------------HHHH111111111HHHHHHHHHHHHHHHHHHHC11CCCC1HHHHHHHH111111111111HCCCCHHHHHHHHHHHCCCHHHHHHHHHHHHHHHHHHHCCCC  &nbspPredictedSECSTR  
MODEL    TVVEAVLPVFVIMLLGLYLAGKWWGILSP-DQASGINKLVVYFALPLLIFSFISENVTLEMILDFWLIPVLVVLI---VLISLIIGFLVWHPTGTKRIFKLPLEWRGVLIL----TSALPNTGFL--GLPLLLALYGELKDLLWDKIPFGNREKCSSEGLSYAIISVVLGVIIIYTLLLFRLIENDFHYKGDDDEENFLFESRGAKRDKSEEFGESTSGSIVSINVDSDVLSLDGSEDPLETEEEIKEDGTLHVTVRKSNASRSDIVSTSSEERGGVGGDSSTPRPSNLTGAEIYSLQSSRNTTPRGSSFNHTTDFYRTSHVHNNRRNSITGSLRSISMMELPAGRLSNFGPADAYVDQYGRRRRKSSISVQGSRGPTPRPSNFEENLARASTLRFGTNSAAAGAHYPAANPKGMSGPKTAAKAPKKASANNGKAAAEKAKEAAAVVKGKDQSVVSSSASSDRDGLQLDAGANSASESSDQGGGGGRDYADDDNKKVPEKKDGKKDGGQGAEQEDAAVERASQSLGKAGLDSEAAADAAGSKEDEETSEVKLMPPTSVMTLLILIVVLLKLILNPPTYASLLGLI----------LALVFGFKLPLIFPTDAPLKIKQAEEDAFVEEIIQKSISILGDAAIPMALFSLGLFLALGKLKIALGAATATIAVDKSLIVLLILRLILVPLVMLGIVLLLGGSLRGLILLKVAIVQDDPMFLFVLLLQAALPPAIVLFVLAKLYNVDEEEASTVVLWTTLLALLTLPLWI  &nbspHMMER2[PF03547.13\_Mem\_trans\_seed]  
QUERY    KFFKYVPAIVLIYLGAAFMNT-F-GLFGDSESLSNTSYGVRNALLPAMILLMLLQCDLRKIIKLGPKLLITYFVAAFSIVLGFTLTYLV-----MQ-SFFLDDTWRAFSALAGSWTGGSANMVALQDILAVPETIFGY------------------------ALIMDTINYSFWVMVMF---WLVPFE---------RMFN-------------------------------------------------------------RWTKADTSKLE----------------------------------------------------------------SMSQ----------------------------------------------------------------------------------------------------EIAATVTDEKREPT------------------------------------------------------------------------------------------------------------TFVHMIGLLGFSLFIAALATVIGENLPQIGTGINAM-TWTI-LIVSIVGLL------------LALTPFASIPGSMDIGRVMLYT--IVA----IIASGADFSSIGEIPVYIIAGFMVLLFHGLILFGFAKLFK--LDLF-TLGVASL-------------ANIGGMVSAPVLAGAFNRA--LIPVGVIMALIGGFMGTWFGV   Q9K8K1|Unknown|Q9K8K1\_BACHD[29 379]  
  
  
**PF07884.9\_VKOR\_seed:**  

**E:4.32e-02[Original]  &nbspE:7.51e-04[Fold-critical]  &nbspE:7.64e-03[Remnant]  &nbspRatio[FC/R]:9.83e-02  &nbspClassification:TP**

TM       ----ccccccccccccccccc------------------------------------------------------------ccccccccccccccccccccccc-----------------ccccccccccccccccccccccccc--cccccccccccccccccccccccccc---  &nbspTMSOC  
STRUCT   ?SHHHHHHHHHHHHHHHHHHHH---HHH???-----?S?????-----????--SGGGSSSSEE--TT-------------EEHHHHHHHHHHHHH-HHHH?????T---------T?STTHH-HHHHHHHHHHHHHHHHHHHHHH--HTS?-???HHHHHHHHHHHHHHHHHHSS?  &nbspPDB/DSSP  
MODEL    TRWIILILGLIGLLASLYLTLE---KLTLLELDPGYVASCDINEAKPVVSCSCVKVLNSPWATIAKFGLLGSILGNDSLLNIPNSLLGILAYLVVLYLLGVLPLAGVLSENKKLRKTLSRWTWRLGLFLGSLVGVVFSLYLIYISLQAFVIGGALCLYCILSAVVSIILFILSTLGV  &nbspHMMER2[PF07884.9\_VKOR\_seed]  
QUERY    MTWTILIVSIVGLLLALTPFASIPGSMDIGR-----VMLYTIV---AIIASG------ADFSSI--GE-------------IPVYIIAGFMVLLFH-GLILFGFAKL--------FKLDLFTL-GVASLANIGGMVSAPVLAGAFN--RALI------PVGVIMALIGGFMGTWFGV   Q9K8K1|Unknown|Q9K8K1\_BACHD[250 379]  
  
  
**PF00083.19\_Sugar\_tr\_seed:**  

**E:5.20e-02[Original]  &nbspE:7.37e-06[Fold-critical]  &nbspE:1.61e-07[Remnant]  &nbspRatio[FC/R]:4.58e+01  &nbspClassification:TP**

TM       --------------------------------------------------------------------------------------------------------------cccccccccccccccccccccc--cc---------ccccccccccccccccccccc--------------cccc--------ccccccccccccccc-ccccc--------------ccccccccccccccccc---cccccc-------------------ccccccccccccccccccccc--------------------------------------------------------------------------------ccccccccccccccccccccccc---------------cccccc----ccccccccccccccc----ccc-------------cccccc-------cccccccccccccccccccc----------------cccccccccccccccccccccccccc-----------cccccccccc--cccccccccccccc-----------cccccccccccccccccccccc---------------  &nbspTMSOC  
STRUCT   HHHHHHHHH-HHHHHHHHGGGGTHHHHHHHHTGG-G???HHH------------------------------------------------------------------HHHHHHHHHHTHHHHHHHHHHHHH--HHHHHT?HH-HHHHH-HHHHHHHHHHHHH?TTTTTS?SSSSSS??GGGGG?HHHHHHHHHHHHHHHHHHHHHH-HHHHHTTS---?GGGH-HHHHHHHHHHH-HHHHHHH---HHHHHHH-HTTS?TT--TTTTTHHHHHHHTTHHHHHHHHHHG-GGS???HHHH-HHTT?HH-HHHHHHHH---HHHH----HHHHHHHHHHHHHHHHHHHHT--------THHHHS??T------HHHHHHHHHHHHHHT?HHHHHHHHHHHHHHS-S??HHH-HHHHHH----HHH-HHHHHHHHHH-H---HHHHHH?SH-------HHHHHH-------HHHHHHHHHHHH-----HHHHTT??----------HHHHHHHHHHHHHHHHTTTTHHHHHHHHHSS?TTTHHHHHHHHHHHHH--HHHHHHHTHHHHH?HHHHHHHHHTT?HHHHHHHHHHHHHHHHHHHH????TT??HHHHGGGT  &nbspPDB/DSSP  
MODEL    VALVAALGGSFLFGYDTGVIGAFLTLISFLKRFGNALTSSGADAELSSYKCEWTDTSVTVSNTTYGEVCGWADRTTCFLKYSDEAGCLSDSCSKASDSENECKNYVGYSTVLTGLIVSIFSVGCLIGSLFAG--WLGDRFGRKRKSLLISANVLFVIGALLMGAAKGKGTSIIGGASVYMLIVGYVPEFVIVRVIVGLGVGGISVLV-PMYISEIANEYPKKLRLGALVSLYQLAIRTFGILVA---AIIGLGLINKTSNEAEEALSSDGWRIPLGLQLVPALLLLIGLLLFLPESPRWLKVEKGKLEIEAREVLAKSLLLRGVEVKESDVDQEIQEIKDELEASQEEEKLKAGKASWLELFSRKTRPKYRQRLLMGVMLQIFQQLTGINAIFYYSPTIFESL-GVSDSTLSLLVTI----IVGKVVNFVFTFIALIFLGFLVDRFGRRTILLIGLPLLLLGLPGMTVAAAGMAICMVVLGASVIVALLGVAKSKEGDPSSKGAGIVAIVFILLFIAFFALGWGPVPWVIVSELFPLGVRPKAMAIATAANWPLLANFLIGFLFPIITGAIGLERDFLGGYVFLVFAGLLVLFIIFVFFFVPETKGRTLEEIEELF  &nbspHMMER2[PF00083.19\_Sugar\_tr\_seed]  
QUERY    FLFLSILIG------MTALIVWAEYKIG-GKFFK-YVPAI-------------------------------------------------------------------------VLIYLGAAFMNTFGLFGDSESLSNTSYGVR-NALLP-AMILLMLLQCDLRKIIKL-----GPKLLITYFVA--------AFSIVLGFTLTYLVMQSFFLDDTWRAF-SALA-GSWTGGSANMV-ALQDILAVPETIFGYALIMDTIN-----------------YSFWVMVMFWLV-PFERMFNRWT---------KADTSKLE------------SMSQEIAATVTDEKRE-------------------PTT-----FVHMIGLLGFSL------FIAALATVIGENLPQIGTGINAMTWTILIVSIVG-LLLALTPFAS-I---PGSMDIGRV-------MLYTIV-------AIIASGADFSS--------IGEIPV----------YIIAGFMVLLFHGLILFGFAKLF-KL--DLFTLGVASLAN-IGGMVSAPVLAGAFNRALIP----------------VGVIMALIGGFMG--TWFGVL---------TAEIL   Q9K8K1|Unknown|Q9K8K1\_BACHD[6 385]  
  
  
**PF00115.15\_COX1\_seed:**  

**E:7.88e-02[Original]  &nbspE:5.78e-05[Fold-critical]  &nbspE:1.23e-08[Remnant]  &nbspRatio[FC/R]:4.70e+03  &nbspClassification:TP**

TM       ------ccccccccccccccccccccccc---------------------------------------------ccccccccccccccccccccccccccc-----------ccccccccccccccccccccc------ccccc--------------------------------------------------ccccccccccccccccccccccc--cc--------------cccccccccccccccccccccccccccccc------------------------cccccccccccccccccc------------cccccccc--------ccccccccccccccccccccccccccc----------ccc-ccccccccc----------cccccc-----------ccccccc-------------------------ccccccccccccccccccccccccc-------------cccccccccccccccccccccc----ccc---------------ccccccccccccccccccccccccccc---------------------------------------------------  &nbspTMSOC  
STRUCT   HHH-HHHHHHHHHHHHHHHHHHHHHHHHHHT--SSS------------------S?S---SS?H---HHHHHHHHHHHHHHHHTTHHHHHTTTHHHHHHHHHTT?SS?SSHHHH-HHHHHTHHHHHHHHHHHH------HSTT??--------------?S?TT??T-TTT-STTT-----T?------SSHHHHHHHHHHHHHHHHHHHHHHHHHH--HHHSS?TT??GGGS-?HH-HHHHHHHHHHHHHHH-HHHHHHHHHHHHHHHS???SS?GGGT??HHHHHHHHHHHHHHHHHHHHHH-HH------------HHHHHHHHHHTT?SS?TTHHHHHHHHH-HHHHHT-TS?GGGG?TT-S???HHHHHH-HHHHHHHTH----------HHHHHH-----------HHHHHHH------HTT?----?----????-HHHHHHHHHHHHH-HHHHHHHHHHHSHHHHHHHTTBHHHHHHHHHHTHHHHHHHHHHHHHH----HHHHHHSB-----???HHHHHHHHHHHHHHHHHHHTHHHH-HHHT-T?BSS?----SS?------?GG-------GHHH----HHHHHHH--HHHH  &nbspPDB/DSSP  
MODEL    HKDKIGLLYLVTALVFFLIGGVLALLIRLQLAPATPRYDLVLGSPFSPGHLQASGDFNAELSPDAWLLTYNQLRTLHGNIMIFFFAMPALIGGFGNYLVPLMIGARDLAFPRLNLALSFWLLVVGGLLALVSLVGAYGLLLGGGAGLANITGNFWLGTPGTGWTEYPVPLSTSTTSNLNAVSSGEGNQLGLGVDLWILGLLLVGISSILGAINFIVTGVILKMRAPGMTLSRMVPLFAVWSLLATAILLLFAFSPVLAVALLMLLLDRNFGTSFFDPAGGGDPLLYQHLFWFFGHPEVYILILPIAF------------GIVSEIIPTFSGRKPLFGYKWMVLAMVFAIAFLGGFLVWAHHMFTDSGLPPWLRAF-FSVATMLIAALASMIHALVIPTGVKAARRRKGLNNGVFNWLATLERAGRLWGGWRKHREVFKIRFSRTPMLFALGFIVLFFTIGGLTGVMLALVPLDYQLHDTYFVVAHFHYVLYGGAVFALFAGIYYFCWKWFPKLTGRKEDGEMYNERLGKLHFWLTFIGFNLTFFPMHFPLGLLQGMPRRVSSVVADYPYARFMPPQEAVPGREFAPWNLSINLLRTIGVAGFLL  &nbspHMMER2[PF00115.15\_COX1\_seed]  
QUERY    AEYKIGGKFFKYVPAIVLIYLGAAFMNTFGL--FGD------SE-----------S----LSNTSY-GVRNALLPA----MILLMLLQCDLR--------KIIKLG----PKLL-ITYFVAAFSIVLGFTLTY------LVMQSFFLDDT---------------------------------------------WRAFSALA-GSWTGGSANMVAL--------QDILAV-------PETIFGYALIMDTIN-----------------------------YSFWVMVMFWLVP------FER-MFNRWTKADTSKLESMSQEIAATVTDEKR---EPTTFVHMIGLLGFSL-FIAALATVIG-ENLPQIGTGINAMTWTILIV------------SIVG-----------LLLALTP------FASIP--GS----MDIG-RVMLYTIVAIIAS--------------GADFSSIGEIPV-----YIIAGFMVLLFHGLILF----GFAKLFKL----DLFTLGVAS----LANIGGMV-SAPVLA-GAFN----RAL----IPV------GVI---MA--LIGG-----FMGTWF--GVLT   Q9K8K1|Unknown|Q9K8K1\_BACHD[22 381]  
  
  
**PF09847.4\_DUF2074\_seed:**  

**E:8.15e-02[Original]  &nbspE:1.15e-03[Fold-critical]  &nbspE:1.02e-07[Remnant]  &nbspRatio[FC/R]:1.13e+04  &nbspClassification:TP**

TM       ------cccccccccccccccccccc----------cccccccccccccccccccccc-------------------------ccccccccccccccccc--cccccccccc--------ccccccccccccccccccccccc----------------------cccccccccccccccccccc---------------------------------------ccc--cccccccccccccccc----------------------------------------------------------------------------------------------------------------------------------------ccccccccccccccccccccc-------ccccccccccccccccccccc----------------------cccccccccccccccccccc---cc-----------ccccccccc----ccccc----  &nbspTMSOC  
STRUCT   CCHHHHHHHHHHHHHHHHHHHH---------------11HHHHHHHHHHHHHHHHHHHHH11111111111111111111111EEEEEEEHHCCHHHHHH11HHHHHHHHHHHHH----HHHHHHHHHHHHHHHH1HHHHHHHHHHHCCEEECCCHHHHHHHHHHHHHHHHHHHHHHHHHHHHHHHHH11-------------EE11-------------11HHHHH11HHHHHHHH1HHHHHHHHHHHHHHHHHC11CCCCEEEEEEEEEEEEECCCCCHHHHHHHHHHHHCCCCCHHHHHHHHHHHHHHHHH--------------HHHHHHHHHHHHHHHHHHHEEEEE-----------EEECCC--CCCCCHHHHHHHHHHHHHHHHHHHHHHHHHHHHC1CCCHHHHHHHHHHHHHHHHHHHEEE-----------1111111111111HHHHHHHHHHHHHHHHHHHHHHHHHHH111111HHHHHHHHH1111HHHHHHHCC  &nbspPredictedSECSTR  
MODEL    NIKLSVLLQSIMFTFFGLVLVVPSLISERIVQRGVFLSSFLILLFIYSLFITALNSAYFASSISINKLLEPLRSLPIKLLGRVLSVFWLIDTLPSFAFLVLPSIFIAVLLVGNIY----SGLLGLLWSILAILLGAHSLGLLLFIKFGKRISGRGSRSKALLRIFGRLLFLVFIFGIYLLIQYNADIVKQNYEIIPEIPEYYEYIFPILNIDKTVFYALTIYEPFKSKYLLLSLLYTGLLFFFLYKYSIRRLWERLLEPEGEKVKGSVKTEYKIKVRSPLLAFLIKDFKIILRKSQLLVLLLMPIFIVIPNIYSIAKEGFPLRVDPILLTIFLIAFIIVLSSIYLILFLKIEVNGFMSLERSSLLRSLP--LSRREFLLSKLLLILLIYLAISLTLLILVLIYKGFTNALYLLILFPSPILTSGISLLIVYNRLIKKIPKGAETINLPSLGGFLAFIVLFILNGIIVGIVAILSFILSEPLSLSIKLFFLVSTAITLAVLAIIALLLWKK  &nbspHMMER2[PF09847.4\_DUF2074\_seed]  
QUERY    MIENGFLFLSILIGMTALIV-WAEYKIGGKF--------FKYVPAIVLIYLGA---AFMNTF----GLFGDSESLSNTSYGVRNALL------PAMILLM-----LLQCDLRKIIKLGPKLLITYFVAAFSIVLG-FTLTYLVMQ----SF-----FLDDTWRAFSALA------GSWTGGSANMVAL-QDILAVPETIFGYALIMD------------TINY---S-FWVMVMFWL-VPF--------ERMFNRW-------TKADTS---KLESMSQEIAATVTDEK---REPTTFVHMIGLLGFSLF-IAALA----TVIGENLPQIGTGINAMTWTILIVSIVGLLLALTPF---------ASIPGSMDIGRVMLYTIVAIIASGADFSSIGEIPVYIIAG------FMVLLFHGLILFGFAKLFKLDL---------FTLGVASL-ANIGGMVSAPVLAGAFNRALIPVGVIMALIG------GFMGTWFGV----LTAEILSRL   Q9K8K1|Unknown|Q9K8K1\_BACHD[1 388]  
  
  
**PF07556.6\_DUF1538\_seed:**  

**E:1.00e-01[Original]  &nbspE:3.16e-04[Fold-critical]  &nbspE:1.42e-09[Remnant]  &nbspRatio[FC/R]:2.23e+05  &nbspClassification:TP**

TM       -----------------------ccccc-cccccccccccccccc-------------------------------------------cccccccccccccccccccccccc--------------------------ccccccccccccccccccccccc-----cccccccccccccccccccc-----cccc------cccccccccccccccccccccccc--------------ccccccccccccccccccc--cccc-  &nbspTMSOC  
STRUCT   CEEEEHHHHHHHH1CCCHHHHHHHHHHH-HHHHHHHHHHHHHHHHHCCCCHHHHHHHHH-----------------------------HHHHHHHHHHHHHHHHHHHHHHHHHHHHHHHHH111111------------HHHHHHHHHHHHHHHHHHHHHHH1CCHHHHHHHHHHHHHHHHHHHCC11111CCHHHHH11111111111HHHHHHHHHHHHHHH---------------11HHHHHHHHHHHHHHHHH--HHHCC  &nbspPredictedSECSTR  
MODEL    IILVILFFQFLVLPKLPLPNLIRILVGL-LLVILGLTLFLVGLELGLFPIGESMGSALAKPEFIEEIAAAELVTASLPLLLKGSAVVFLWLLLLFGFLLGFATTIAEPALIALAEQAEEVSGSGGAISSTQEALNSYAKVLRLTVAIGVGIGIALGVLRIVYPGIPLHYLIIPGYLLVLILTFFAPLDAELKEFVGIAFDSGGVTTGPVTVPLILALGLGLASAKIEASAGSGGRNPLIDGFGLIALASLGPIIAVLI--LGILS  &nbspHMMER2[PF07556.6\_DUF1538\_seed]  
QUERY    LLG-FSLFIAALA-TVIGENLPQIGTGINAMTWTILIVSIVGLLLALTPF-----ASIPG---------------------SMD--IGRVMLYTI--------------VAIIASGADFSSI--GEIP-------------VYIIA---GFMVLL------F-----HGLILFGFAKLFKLDLFTLGVASLANIGGM-----------VSAPVLA------GAF-NR------------------ALIPVGVIMALIGGFMGTWF   Q9K8K1|Unknown|Q9K8K1\_BACHD[222 377]  
  
  
**PF03611.9\_EIIC-GAT\_seed:**  

**E:1.28e-03[Original]  &nbspE:1.03e-05[Fold-critical]  &nbspE:9.49e-13[Remnant]  &nbspRatio[FC/R]:1.09e+07  &nbspClassification:TP**

TM       ----cc--ccccccccccccccccc---------ccccccccccccccccccccccccc---------------------------------------------ccccccc--cccccccccccccc------------cccccccccccccccccc---ccccccccc--------------ccccccccccccccc-cccccccc--------------ccccccccccccccccc----cccccc-------------------------------------------ccc---cccccccccccccc--------------cc--------------------------------------------------ccc--ccccccccccccccccccccccccc-------------------------------------------cccccccccccccccccccccccccccc----cccccc------cccccc-----ccccccccccccccc-----------ccccc-------ccccccccccc---cccccccc-----ccccccc---------------------------------------------------------------------------------------------  &nbspTMSOC  
STRUCT   CCCCCC11CHHHHHHHHHHHHHHHHCCCCCHHHHHHHHHHHHHHHHHHHHH1HHHHCHHHHHHHHHHHH1111111111111111111HHHHHHHHHHH11111HHHHHHH--HHH1HHHHHHHHHH111HHCCC--CEEEECHHHHHHHHHHHHHH---HHHHCC1CH---------------HHHHHHHHHHHHHH-HHHHHHHHHHHHHHHCC11CCCEEEECHH111111HHHH1111HHHHHHH11111111111111111111111111111111111111111HHHH---HHHHHHHHHHHHH---------------HH11----------------------HHHHH11HH--------------111HHH11HHHHHHHHHHHHHHHHHHHHHHHHHHHHHHHHHHHH11111--------------1CCCC-CCCC--EECCC1CCHHHHHHHHHHHHHHHHHHHHH11111H------------HEE11111111111111111HHHH-----------CCCC111CCHHHHHHHHHHHHHH---HHHHHHHH11111HHHHHHCCC11111--------------------------------------------------------------------------------111111  &nbspPredictedSECSTR  
MODEL    VNNILSTLGPAVLLGLIALIGLLLLKKKFSKALKGTLKTGIGFLILGAGAGPLLVGSLGPFAKAFVEAFGLSGKSSAQQVVPDNGWPAAEAIAALAQQTLFNAGFGSEVAL--IMIPLGFLVNILLALLRRFTKF--KYIFLTGHIWNFMAAMIAGV---LVYAGTSGNQNLLDDKEGVSVGDQWWLIIIGAVILGLY-MLISPALTQPYVRKVTGAPNDGIAIGHFSTATSMAPLGYALFALLSGLIGKLEEKKKSFGEKGKLKDPKKKDKKVKSTEDIKLPKKLGFFRDSTV---AGAILMLILFLIAGPHPIAGVADADKTVLFILFAAGLANVLVPKLDEFKRYARGPEFVESEELSAKEIGAAGKFNAGGQNFVVWGYILIQGITFAAGVAILLRGVRMFLAELVPAFKGISEKLVPGFKENKNIDLDGDGREAKPA-LDCP--VVFGYGAPNAVLIGFLSSFIGGLVPITILIGVLNGALGKIPIGAISLAVILAIPKVGNVPLFFAGAAAGVF-----------GNATIVVGGRRGAIIGSFINGLI---ITFLPALLIGLEYLPVLGPLGTAMAKAVGAALLDKKALMGNSLDNNNGSALKTAVEKVVNATGVNRITEAQSFVKSDSFNSLKAVNSTLAQAIEIISKSAPPGANTTFGDADF  &nbspHMMER2[PF03611.9\_EIIC-GAT\_seed]  
QUERY    L--FLS-----ILIGMTALIVWAEYKIGGK-----FFKYVPAIVLIYLGAA-------------FMNTFGLFG---------DS-----ESLS--------NTSYGVRNALLPAMI-LLMLLQCDLR---KIIKLGPKLL-----ITYFVAAFSIVLGFTLTYLVM-QSFFLDDTW-------RAFSALAGSWTGGSANMVALQDILAVP-ETIFG--YALIMDTINY---SFWVMVMFWLVPFERMFNRW--TKADT---SKLE--SMSQEIAATVTDEK--------REPTTFVHMIGLLGFSLFIAAL--------------AT-------------------------------VIG------------ENL------PQIGTGINAMTWTILIVSIVGLLLA------------LTP-FA-------------SIPGSMDIGRVMLYT--IVAIIASGAD------FSSIGEI-----------------PVYII--A--GFMVLLFHGLILFGFAKLFKLDLFTLGVASL--ANIGGMVSAPVLAGAFNRALIPVGVIM-----ALIG---GF---------------------------------------------------------------------------------MGTWFG---V   Q9K8K1|Unknown|Q9K8K1\_BACHD[7 379]  
  
  
**PF03169.10\_OPT\_seed:**  

**E:3.73e-02[Original]  &nbspE:3.76e-04[Fold-critical]  &nbspE:1.96e-10[Remnant]  &nbspRatio[FC/R]:1.92e+06  &nbspClassification:TP**

TM       -----ccccccccccccccccccccc---ccccccccccccccccccccc---cc------------------------------------------------------------ccccccc----ccccc-----ccccccccccc------------------------------ccccccccccccccccccccccc------------------------------------------------------------------------------------------------------------ccccccccccccccccccccccc----------------------------ccccc---cccccccccccc--cccccccccccccccccccccccccccccccc-----------------------------------------------------ccccccccc--cccccccccccccc-------------------------------------------------------------------------------------------------------cccccccccccccccccccccc-----------------cccccccccccccccc-cccccccc----------------------ccccccccccc-----------------ccccccccccccccc-------------------------cc----ccccccccccccccccccccc---------------------------cccccccc--cc--------------ccccccccccccccccccccc---------------------------------------------cccccccccccccccccccc-------cccccccccccc-------------------------------------------------------cccccccccccccccccccccc-  &nbspTMSOC  
STRUCT   CCHHHHHHHHHHHHHHHHHHHHHHCCCCCCCEEHHHHHHHHHHHHHHHHH11H1--------------------------------------------EEE11111111111HHHHHHHHHH11111111111111111HHHHHHHHHHHHH-------------------1111111HHHHHHHHHHHHHHHHHHHHHHCCCEEE1111111111111111HHHHHHHHHH11111-------------------------------------------------------------------11HHHHHHHHHHHHHHHHHHHHHHHHHCCC1CCH11--------------------------------11111H-----------HHHHHHHHHHHHHHHHHHHHHHHH11111111--------------11111-----------111111HHHHHHHHHHHHHHHHHH11111HHHHHHHHHHHHHHHHHHHHHHHHH1111-------------------------------------------------11HHHHHH1111---------------------11HHHHHHHHH1HHHHHHHHHH----------------------HHHHHHHHHHHHHH-HHHHHHHHCCC11C1CCHH11111111HHHHHHHHHHH---------------------HHHHHHHHHHHHHHHHHHHHHHHHHHHHHHC1CCCHHHHHH1111HHHHHHHHHHHHHHHHHHHH11H111111111111111111111111HHHHHHHHHHHH1111111111111111HHHHHHHHHHHHHHHHHHH----------------------------------------------HHHH------------HHHH1111111HHHHHHHHHHHHHH---------------------------------HHHHHHH------------1HHHHHHHHHHHHHHHHHHHHHCC  &nbspPredictedSECSTR  
MODEL    ELTFRAVVLGILLAILGAAVNMYFGLRTGLVSISSIPAALLAYPLGKLLAKRRILPDDEYVEYVDGCRTDSANIPVDHPTDEQPRVFVKLTWKFTLGGIRFSLNIPGPFTIKENNLIQTMASAATFAGASSTGSGIALAAGIIFVLPALLFYGAEGESDPSGGGGDDRRGGQSFAGNSFGYAILLLLSTQLLGVGFAGPLRRFLVYPEHPKLPFGANEAWPSGLATAELLKALHTPGNHEVGAASELEERRERRRDESDEVLRSRRDPERADYLEEDEASEDAEAEAEEEKDKDISANGWKEAKSRLKFFLIGFVASFVYYWFPFYIFPALSSFCSWVCWIAPKAKMSSNPTFGLLFGGFGGLGGMGLLNFTFDWSAILGYLGSGLIVPLWVAVNMLIGAVLSWGILIPLLYYSGNVWYTAYLPSIMSNGLFDNTGASYNVNVYNESRILTSDGLLDYARYIGYGPMLLSGLYALGWSYGLFFAAITATIVHSILFHGRDIWQALKSFALEIWKAEVKKAREWVAVSYSASKPKSFPKQSKSSRRGPKKSKEEDVGRDDPHRRLMRKYKEIEVDISKQLADTQVANRLKHLVPMWWYLAGLVGLSLVLGIALVAALFGQTQLPVTRDFLATVILWGLLLALLLAFVFAI-PSAYIAGLTGSSNNPVSGLKGPMDARANILTELIAGYLLPGRGVKLLLVPLTVEQGPGGPLANLIFGGVGYNAAAQAGDFMQDLKTGHYMRGAPPRAQFVYGRRAQLIGTLVGSVVNPAVLNLLIDNQAYGCTGAQGDPGMDNSWTCPAMPQAFFSAAVAWGVIGGKRLFHTSGFGLPYYALLWGFLVGAVAPVLDWLLKVAASFIANGGRAVIKPWRRHKRVLPRSKWKKVKLSRRLSQLNPPLAFAGAGTQIGPYLPPSLSSMNPWAGWAILVGGLFNYYVRRAHTNHTKKDDGFKPATEPAQSVLSRRPVESERRKAWWRKYERKDEDLERAKQKNYVLAAGLIAGEALMGVIIAFLV  &nbspHMMER2[PF03169.10\_OPT\_seed]  
QUERY    ENGF--LFLSILIGM--TALIVWAEYKIGGKFFKYVPAIVLIY-LGAAFM-----------------------------------------NTFGLFGDSESLS-----------------N---------T----SYGVRNALL-PAMILL-----------------------------------------MLLQCDLRKIIKLG--PKLLI---------TYFVAAFSIVLGFT------------------------------------------------------------------------LTY--LVMQSFFL-DD----------TW-------------RAFSALAGSWT----------GGSANMVA--LQDILAVP-----ETIFGYA---LIMDTI-NYSFWVM-------------------------------------------------------VM-----------------FWLVPFERMFNRW--------------------------------TKADTSKLESMSQE------IAATVTDEKREPT-------------------TFVHMIGLLGFSL-FIAALATVIGENLP-QIGTGI------NAMTWTILIVSIVGLLLALTPFASIPGSMDI--GRVMLY--------TIVAIIASGADFSSIG----------------EIPVYIIA-------------------GFMV--------------LLFHGLILFGF-----------AKLFKLD----------LFTLGV-------ASLAN--IGG--------------MVSAPVLAGAF----NRAL------------------------IPVG-----------------VIMA---L-IGGFMGTWFGV-------------LTAEILSR---------------------------------------------------------------------------L   Q9K8K1|Unknown|Q9K8K1\_BACHD[3 388]  
  
  
**PF02028.12\_BCCT\_seed:**  

**E:1.18e-02[Original]  &nbspE:4.33e-08[Fold-critical]  &nbspE:8.67e-21[Remnant]  &nbspRatio[FC/R]:4.99e+12  &nbspClassification:TP**

STRUCT   HHHHHHHHHHHHHHHHH-----SS-SHHHHHHHHHTTHHHHHHTHHHHHHHHHHHHHHHHHHHHSG-GGG??S-SSTT???SS?HHHHHHHHHHHH??HHHHHHHHHHHHHHHHT?----------------?--TTS?-----------SS?H-HHHHHHHHHHHSHHHHHHH-HHHHHHHHHHTTT-S???----------------SSGGGGG-TTTS?-T-TT-??SS----SSHHHHHHHHHHHHHHHHHHHHHHHHH----------HHHHHTTTS-?-------?--??-?------??HH-HHHHHHHHHHHTTGGGTTS-??S--HHHHHHHHHHHHHHHHHHHHHHHS?-HHHHHHHHHHHHHHHHHHH------------HHHHT??---TT---TT------SS?H----------------------------------------------HHHHHTTHHHHHHHHHTTHHHHHHHHHHHTTT??HHHHHHHHHHHHHHHHHHHHHHHH------HHHH---------HHHH-----HT?--????--------------TT?S-HHHHHHHHHTS-------------TTHHHH----HHHHHHHHHHHHHHHHHHHHHHHHHHTTTT---?S--S??HHHHHHHHHHHHHHHHH-HHHH--HGG----GHHHHHHHHHHHHHHHHHHHHHHHHHHH---------------------HHHHHT?  &nbspPDB/DSSP  
MODEL    VFIISALIILLFVLWGILPESLIFDPEAAGAVFNTLFAWITNNFGWFYLLLVLFFLVFLLFLAFSRKYGNIRLVGGDDEKPEFSTFSWFAMLFSAGMGIGLVFWGVAEPLYHFLSPFKLLGTAENTNYIAGTPDIPGGGAELGIEPGTIGPEAAAREAMAYTFFHWGLHAWAIYGALVGLALAYFAYRRKGLPGEKDEEDEEDEEDKGELLISSALRYPLLGDEKKRRIYGPASRVIGKAIDILAVFATVFGVATSLGLGVLQ----------INAGLSYLFPGADDDDDWIMDPPENAGSGSLSLTVAQLIIIAIITALATISAVSG-LDKEVGIKRLSNLNMVLALLLLLFVLIVGPMTLFILNTFVQSLGDYLQNFLSISFGAYGPQDVRMSFRTGAADPLGRFGGLGTDGDDGAGDALRGGATNAWGSFEGFKSGLEGEAAALSDEVLAAAYAAGEPGALAEWLGGWTIFYWAWWIAWAPFVGMFIARISRGRTIREFVLGVLLVPTLFTFLWFSVFG------GTAILAEQLDRQTDLELSASWNNGGGGDLADDKAKYAAGTVLVLVAADVVEAALFALLEQLLFRLNLGGADMSGPLGTILALLASVLAIVLIIIFFVTSADSATLVLAMLTSGGLDSDLDLNPPRWQRVFWGVLLGAVAAVLLLLAGEGGTKGDADALDALQTASIIAALPFSVILLLMCISLDFLQIQWVGALVIILYFCCFTLKALREE  &nbspHMMER2[PF02028.12\_BCCT\_seed]  
QUERY    LF------LSILIGMTA-----LI------------------------------------------VWAEYKI-GG-------KFFKYV-------PAIVLIYLGAA-----FMNTF---------------G--LFG------DSES--LSNT-SYGVRNA-----LLPAMIL-------LMLLQCDLRKII--------------------------KLGPK--LLITYF-----------VAAFSIVLGF--TLTYLVMQSFFLDDTWRAFSALAGSWT-G-------G---S-A------N-MVALQDILAVPETIFGYALIMDTINY---------------SFWVMVMFWLVP-FERMFNRWTKADTSKLESM---------SQEIAATVTD---EK---RE------PTT-------------------------------------------------FVH---------------------------------MIGLLGF-SLFIAALATVIGENLPQIGTGI---------NAMT---WTILIV-SIVG-----L-------------------LLALT-------------PFASIP----GSMDIGRVMLYTI---------VAIIASG----AD-----------------------FSSI--GE-------IP----VYIIAGFMVLLFHGLILFGF---------------------AKLFKLD   Q9K8K1|Unknown|Q9K8K1\_BACHD[7 330]  
  
  
**PF13303.1\_PTS\_EIIC\_2\_seed:**  

**E:6.49e-02[Original]  &nbspE:7.45e-04[Fold-critical]  &nbspE:4.41e-11[Remnant]  &nbspRatio[FC/R]:1.69e+07  &nbspClassification:TP**

STRUCT   CCCHHHHHHHHHHHHHHHHHHHHHHHHHCCC1111111111H-------------------HHHHHHHHHHHHHHHHHHHHHHHHCCCCHHHHHHHHHHHHHHCCCEEE---------------------------EECCCCHHHHHH-HHHHHHHHHHHH11111111111EEEHHHHHHHHHHHHHHH1HHHHHHHHHHHHHHHHHHHHHHCHHHHHHHHHHHHHHHHHCH1HHHHHHHHHHCCCC---------------------HHHHHHHHHHHHHHH11HHHHHHHCCCCCCCHHHHHHCCCCCCCHHHHHCCH1H-HHHHHHHHHHHHHHHHHH1--------------------------HHHHHHHHH11------------------H11HH1HHHHHHHHHHHHHHHHHHHHHHH------------------------HHHH11111111111  &nbspPredictedSECSTR  
MODEL    IKVLNGMALGIFVTLIPGAILGTIGKLLGILFPIFALALVSSTGELAAKVAGLSMLNRFCWFLVQIGTLAQSLLGPAIGVAVAYQLKANPLVSFSAGIAGFIGSGAVKFVQTASVATATKTATGGVNATEGGVVDPLAGTGDPINAFI-AAAIAVLVGKLISKNLGGKTKLDIILVPIVVILVGGLIGLLVILPYVKPITTAIGNVINSATDLQPLLMGILIAVIFGILITSPFISSAAIAIALGLTGFGVGNALAIPDPIAIVWNGLALAAGAAAIGCCAQMVMNGLAVASLKVNGLGGLIAQGLGTPKLQMPNILKNPIPI-LLPPLLAAAILGPIATLLFDGYQWFIGIQPNGTPASAVWAGFGTSGLVGPIAALNLMAVNGTVRDVADKMAGTGPSAGTVILLIILVFFVLPAVLALLIYKLFKGFAKKDYKLDEIPTNEINEKNDDIKKLGLIKPGDTLKL  &nbspHMMER2[PF13303.1\_PTS\_EIIC\_2\_seed]  
QUERY    LESMS----------------QEIAATVTD-----------E--------------KREPTTFVHMIGLLGFSL-------------------FIAALATVIG--------------------------ENLPQ---IGTG--INAMTWTILIVSIVGLLLA------------LTPFASIPGSMDIGRV--------MLYTIVAIIASGAD-------------FSSIGEIP---------------VY----------------------------IIAGFM--VLLFH--------GLILFGF----------AKLFK-LDLFTLGVAS-----LANIG--------GMV--SAPVLA------------------------------------GA--FN--RALIPVGVIMALIGGFMG-----------------------------TWFGVLTAEI-LSR   Q9K8K1|Unknown|Q9K8K1\_BACHD[195 387]  
  
  
**PF03806.8\_ABG\_transport\_seed:**  

**E:1.30e-02[Original]  &nbspE:3.08e-05[Fold-critical]  &nbspE:2.86e-23[Remnant]  &nbspRatio[FC/R]:1.08e+18  &nbspClassification:TP**

TM       --------------------------------------------------------------------------------------------ccccccccccccccccc--------------------ccccccccccccccccc-ccccccccccccccccc------cccccccccccc---ccccccccccc----------------------------ccccccccccccccccccc---------------------------------------------ccccccccccccccccccc----------------------------------------------------------------cccccccccccccccccccc----------------------------------------------ccccccccccccccccccc-----------cccccccccccccccccccc--------------------------------------------------  &nbspTMSOC  
STRUCT   CCHHHHHHHCCCCCCHHHHHHHHHHHHHHHHHH----------------------------------EEEEECCCCCCHHHHHHHHCCCCCCCCCHHHHHHHHHHHHHHHHHCCHHHHHHHHHHHCCCCCHHHHHHHHHHHCCCCC-CCCEEECCCHHHHHHHHHCCCCHHHHHHHHH------------------1HHHHCCCHHHHHH111111111111HHHHHHHHHHHHHHHHHHHHH111111111--------------------------HH----HHHHHHHHHHHHHHHHH1HHHH111----------------1HHHHHHHHHHHH--HHHCCEEEEEEEEEEECCHHHHHHHHHHHHHCCCHHHHHHHHHHHHHHHCCCCCCHHHHHHHHHHHHHCCCCCHHHHHHHHHHHHHHHHHHHCCCHHHHHHHHHHHHHHHHHCCCHHHHHHHHHH1111111111111HHHHHHHHHHHHCCCCCHHHHHHHHHHHHHHHHHHHHHHHHHHHH1H111111111111  &nbspPredictedSECSTR  
MODEL    RFLNFVERLGNKLPDPVTLFIILIVLLLVASAILSAFGV-----SVVNPRTGGTKGRFGELEAGDQTIEVKNLLSGEGLALILTNMVKNFTSFAPLGLVLVVMLGVGVAERSGLLSALMRKLVNKAPKKLITPTVVLIGILSHTAS-DAAYVVLIPLAAMIFIALGRHPLAGLAAAFAGVSG---GFSANLLLGTLDPLLAGFTQTAAQIIDPSYAEVVNPLMNWYFIAASVVVLTPIGWFVTDKIVEPRLGPY----KGSLDADEKEELASDELTAEEK----KGLRWAGIAFVVLIALLAALTVIPENGPLRDPETGSVADGSPFMKSIVPLIMLL--FLVPGLVYGRVTGTIKNSKDVVNMMAESMSSMGTYIVLAFFAAQFVAMFNWSNLGPILAVKGAEFLKAIGLPGIPLLLGFILLSAFINLLIGSASAKWAILAPIFVPMLMLLGYSPEFTQAAYRIGDSITNIITPLMSYFPLVLAFAQKYDKKMGIGTLISLMLPYSIAFLIVWILLLLVWVLVLGLPLGPGAPIHY  &nbspHMMER2[PF03806.8\_ABG\_transport\_seed]  
QUERY    -------KIGGK----FFKYVPAIVLIYLGAAFMNTFGLFGDSESLSNT-----------------SYGVRN--------ALLPAMILLM-----------------------LLQCDLRKIIKLGPKLLITY---FVAAFSIVLGFTLTYLV---MQSFFLDDTWR--------AFSALAGSWTGGSANM-VALQDILAVPETIFGYALIMDTI--------NYSFWVMVMFWLVPF---------ERMFNRWTKADTSKLESMSQEIAAT--VTDEKREPTTFVHMIGLLGFSLFIAAL--ATVIGENLPQIGTGINAMT-WTILIVSIVGLLLALTPFA-----------SIPGSMDIGRVMLYTIVA-------------------------IIA-SGADF-SSIGEIPVYIIAGFMVL-----LFHG----------------LILFGFAKLFKLDLFTLGVASLANIGGMVS--APVLA-----------GAFNRALIPVGVIMALIGG-FMGTWFGVL------TAEILS   Q9K8K1|Unknown|Q9K8K1\_BACHD[25 386]  
  
  
**PF01654.12\_Bac\_Ubq\_Cox\_seed:**  

**E:4.01e-02[Original]  &nbspE:5.70e-04[Fold-critical]  &nbspE:7.15e-19[Remnant]  &nbspRatio[FC/R]:7.97e+14  &nbspClassification:TP**

TM       -------cccccccccccccccccccccccc--cccc-------------------------ccccccccccccccccccccccc---------------ccccccccccccccccccccccccccc---------ccccccccccccccccccccccccccc----------------------------------------------------------------------------------ccccccccccccccccccccccccc------------------------------------------------------ccccccccccccccccccccc---------------------------------------------------------------------------------------------------------------------------------------------------------------------------------------------------------------------------------------------------------------------------------ccccccccccccccccccccccccc---ccc----------------------------------------cccccccccccccccccccccccccc-------------------------------------------------------ccccccccccccccccccccccccccc-------------  &nbspTMSOC  
STRUCT   CHHHHHHHHHHHHHHHHHHHHHHHHHHHHHH11HHHHHH11111111111HH1111HHHHHHHHHHHHHHHHHHHHHCCCEEEHHCCCCCHHHHHHHHHHHHHHHHHHHHHHHHHHHHHHHHHHHHCC11CC1CHHHHHHHHHHHHHHHHHHHHHHHHHHCCCC1CCCCE11EE1--------------11111EEEE111CCHHHH-----------------------------HHCCCHHHHHHHHHHHHHHHHHHHHHHHHHHHHHH1--------------------------1111111111111111H1HHH1111HHHHHHHHHHHHHHHHHHH1HHHHHHHHHHHHCHHHHHHHHH11111111------------11111111111111111EEEEE11111111111111111111111EE1EECCCHHHHHH-------------------1111111111111111111-------------------------------------------1111111CCC1111111111111111111111111111-----------------1111111111111111111111111111111111111HHHHHHHHHHHHHHHH1HHHHHHHH11111HHHH1H----------------------------------1HHHHHHHHHHHHH1HHHHHHHH1HHHHHCCCCCEE-EE1----------------11111111111111-----------HHHHHHHHHHHHHHHH1HHHH1HHHHHHHHHHH1111HCCCC  &nbspPredictedSECSTR  
MODEL    LSRLQFAFTASFHFLFVPLTIGLALLLAIMEFMTLYVRTGARTAPGDRADEVRKILYKRMTKFWGKLFAINFAMGVVTGIVMEFQFGTNWSGYSRFVGDIFGAPLAIEGLTAFFLEATFLGLMLFGWDNKRVKSKKLHLLATWLVAIGTNLSAFWILAANSWMQMTPVGYGPEINGIYPFMPEFGGLEPETADGRAELDGVTDFWAVASYLVIQAIIQNPEVAGKVGVIKTDDAVAIFNPSAPYRFVHTVLAAYLTGAFFVAGISAWYLLRARREGDTTVDADGKVVVGEEAPDDAREEGGRKSAEAHVGEAKVEDFARQAVCKSFRIALWFGLIAAPLQIVPLGDLSGLNVAEHQPMKLAAMEGLWDETMDTDQTDKDGLTADEEAAYNPGAGAVAYGDPPLSLFGIPDEPLLEAENSCRGDDAEEANHFAAIEIPYLLSLLAATAKQVGVKVGSEEIQNAWTLKHSLDGVEVKNGLKDLQYGNEKKDGTRDPNLAYYRDIKTGQTADVSPEDAAADPSDEKGIAEPEEKIFPGIIAYDALEKLRAAKKDPAARAAFEAHKNDEATQKAAKEDKYKVGVDLGYGLLLKRYTDDVVKATPEQIAKAAKDYRPHPRVAPVFWSFRIMVGLGFLMGLLLALLGLLGKAVWLRRRRKKKGRLPDKRGRLAPSLEVPHLVLFKNKVDRIYESRWLLRLALLMIPLFPFIANEAGIWIVTEVGRQPWT-VYGNPTGSNGGPPTGVDVVLIQDGVEMIGLRTADAVLSPGYSLSAGQVLFSLIGFVLLYTPVLFVYAEVYLLLRYIRYLFRKGPEE  &nbspHMMER2[PF01654.12\_Bac\_Ubq\_Cox\_seed]  
QUERY    -------------FLFLSILIGMTALIVWAE------------------YKI--------------------------------------------------------G------------------G--KF---FKYVPAIVLIYLGA-----------AFMN-T----FGLF--------------GDS--ESLSN---TSYG-------------------------------VRNALLPAMILLMLLQCDL-----------RKIIK----------------------------LGPKL---LI-----T-YFV----AAFSIVLGFTLTYLVMQSF-FLDDTWRA--------FSALAGSW-T---G---GSANMVALQDI----LA---V----PETIFGYA--LIMD-----T-------INYS-FWV--MVMFWL---------VPFERMFNRWT--KADTS-KLE-SMSQ-----------------------------------------EIAATVTDEKR--------------------------------------------------------------------------------EP-TTFVH-----MIGLLGFS-LFIAALAT-----V--I-G---ENLP-QIGTG---------------------INAMTWTILIVSIV-GLL-----LALT------PFASIPG---------SMDIGRVM---------LYTIVAI------IASGA-DFSSIGEIPVYI--IAG-FMVLLFHGLIL----FGFAK   Q9K8K1|Unknown|Q9K8K1\_BACHD[6 325]  
  
  
**PF01970.11\_TctA\_seed:**  

**E:9.36e-02[Original]  &nbspE:8.36e-04[Fold-critical]  &nbspE:1.50e-13[Remnant]  &nbspRatio[FC/R]:5.57e+09  &nbspClassification:TP**

TM       ----cccccccccccccccccccccccccccccccccccccccccc--------ccccccccccccccccccccccccccc--------------------------cccccccccccccccccccccccccccccccccccccccccccc-ccccccccccccccccc----cc-------------cccccccccccccccccccccc--------------------cccccccccccccccccccccccc------------------------------------------------------------------cccccccccc-----ccccccccccccccc---------------------------------------------------------------------cccccccccccccccccccccccccccc------------------ccccc----ccccccccccccccccccccccc--------ccccccccccccccccccccccc-----cccccccccccccccccccccccc---------ccccc---------  &nbspTMSOC  
STRUCT   CHHHHHHH1HHHHHHHHCCCCCCHHHHHHHHHHH1-------------------HHHHHHHH1HHHHHHHHHCCHHHHHHCCCCCCHHHHHHHCCCCHHHHCCCHHHHHHHHHHHHHHHHHHH1111HHHHHHHHHHHHHHHHH1CCCHHH-HHHHHHHHH1HHHHHCCC1C1HH1111111111111HHHHHHHHHHHHHHH1111111111111111-----------1111111HHHHHHHHHHHHHHHHH1111H1---------------------------1------------------HHHHHHHHHHHH1HHHHHHHHHHH-----CCCCCHHHHHHHHHH------------------------HHHH1H1111-------------------EECCCCCHHH111HHHHHHHHHHHCCCCCCHHHHHHHHHHHH--------------11HH11HHHHH----HHHHHHHHHHHHHHHHHH1HH1H1HHH1HHCCCHHHHHHHHHHHHHHHHHHCC1111CCH1HHHHHHHHHHHHHHHHHHCCCC11111111111111111111111  &nbspPredictedSECSTR  
MODEL    NLLLLLLGAVLLGTIVGALPGLGATMAVALLLPFTFGLGMLEKGLDAGLPTEAAPVSALILLAAGIYYGAIYGGSIPAILLNIPGTPASAATTLDGYPMAKQGRAGRALGLAAIASFIGGLIGILILALLLILLAPPLAKVALKAFGPAEY-FALALLGLTSLIASLSGGGSGLLEKSVSERLLSKYRKGLIAALLGLLLGTVGLDPITGSVPRFITFGIPLGMEYQSYELLGDGISLVPVLIGLFAISEVLRLLERALEKRDEEEETEKEKPKELASYRGGSVPKQKIAGSEGSGLFLPTLGGFSRKELRRSWKAILSRGSLIGTFIGI-----LPGAGATIASFLAYAFVRSDFDRIPLKKKDSEPVLGKEKEAKRLVSSEKNPEEAKDPFIIGKKGAIEGVAAPEAANNALYYAAGGALIPLLTLGIPGSAVTAILLGALLIHGSGELQPGPLLFTENPDLVLVWGL----IASLLIANLLLLILGLPLAIRPLAFARILLRIPYRILYPIILVFCIVGAYAINYLYKNSMIFDVWVMLGFGVLGYLMRKLGFPRSNLLMGVLLAPLIRLSDLRCHL  &nbspHMMER2[PF01970.11\_TctA\_seed]  
QUERY    TYFVAAFS-IVLGF----------TLTYLVMQS--FF--L-----D---DTWRA---------FSALAGSWTGG------------SANMVALQD-------------------------ILA----------VPETIFGYALI-MDTINYSFWVMVM-FW-LVPFE----R-MF---------NRWTKADTSKL----------ESMSQ-EIAA-TV-TD--------EKR-EPTTFVH-MIGLLGFSLFIAA----L------------------A----TVIGENL----PQ-I---------GTGINAMTWTIL-IVSIVGLLLALTPFASIPG-SMDIGRVMLYT------------------------IVAI-IASGADFS--S--I--GE--IPVYIIA---------------------------GFMVLLFHGLILFG---FA-------------KLFKLDLFTLGVASLANIGGMVSAPVL-AG-A-FNR--------ALIPVGVIMALIGGFMGT-------WFGV----------------------------LTAEI--------L   Q9K8K1|Unknown|Q9K8K1\_BACHD[97 385]  
  
  
**PF11299.3\_DUF3100\_seed:**  

**E:9.08e-03[Original]  &nbspE:4.05e-04[Fold-critical]  &nbspE:2.85e-16[Remnant]  &nbspRatio[FC/R]:1.42e+12  &nbspClassification:TP**

TM       -------------------cccccccccccccccccccc--------------------ccccccccccccccccc-ccccc-------------ccccccccccccccccccccccccc--------------------------------cccccccccccccccccccccccccccc------cccccccccccccccccc--cccccc-----------ccccccccccccccccccccccccc--------  &nbspTMSOC  
STRUCT   CEEEE1EECEEEEEECCE1EHHHHHHHHHHHHHHHH----------------1111HHHHHHHHHHHHHHHHHHHH-HHHCCCHHHHHHHH-HHHHHHHHHHH1HHHHHHHHHH1HHH111111111111111111EEEEEE11111111111EHHHHHHHHHHHHHHHHHHHHHHHCCCC1CCHHHHHHHCCCCHHHHHHHHH--HHHHHHC1C1CCHHHHHHHHHHHHHHHHHHHHHHHHHHHHHHHHHHHHHC  &nbspPredictedSECSTR  
MODEL    VVIAEVLIGVVKIPLGPGFSIVLLPMLYALLIGLALGLAKLGKKRKPLKKVISDKQSKFASALLLLALLLLIAKLG-TTVGPNLPKILAAG-PALILQEFGNLVGTILLALPVAVLLLGLKREAIGATFSIGREPNLAIISEKYGLDSPEGRGVLAVYIIGTVFGTIFFSLLASFLASLGIFFHPYALAMASGVGSGSMMAAAS--GALAAAYTPAEMADQILAFAAASNLLTTVTGVYVSLFIALPLAEKLYKKL  &nbspHMMER2[PF11299.3\_DUF3100\_seed]  
QUERY    AALAT-VIGENLPQIGTG----INAMTWTILIVSIVGLLLA------LTPFASIPGSMDIGRVMLYTIVAIIASGADFSSIGEIPVYIIAGFMVLLFH------GLILFGF--A-KLFKL---------------------DLFTLG------------------------VAS-LANIGG-----------------MVSAPVLAGAFNRALIP---VGVIMA------LIGGFMGTWFGVLTA--------EIL   Q9K8K1|Unknown|Q9K8K1\_BACHD[230 385]

---

**User sequence**  

Q9K8K1|Unknown|Q9K8K1\_BACHD  
MIENGFLFLSILIGMTALIVWAEYKIGGKFFKYVPAIVLIYLGAAFMNTFGLFGDSESLSNTSYGVRNALLPAMILLMLLQ  
QCDLRKIIKLGPKLLITYFVAAFSIVLGFTLTYLVMQSFFLDDTWRAFSALAGSWTGGSANMVALQDILAVPETIFGYALI  
IMDTINYSFWVMVMFWLVPFERMFNRWTKADTSKLESMSQEIAATVTDEKREPTTFVHMIGLLGFSLFIAALATVIGENLP  
PQIGTGINAMTWTILIVSIVGLLLALTPFASIPGSMDIGRVMLYTIVAIIASGADFSSIGEIPVYIIAGFMVLLFHGLILF  
FGFAKLFKLDLFTLGVASLANIGGMVSAPVLAGAFNRALIPVGVIMALIGGFMGTWFGVLTAEILSRL

---

**Main reference**  

1) Wing-Cheong Wong, Sebastian Maurer-Stroh, Birgit Eisenhaber, Frank Eisenhaber, 2014,
*The necessity of dissecting similarity scores for inferring homology: an essential issue in protein function prediction and annotation*
, BMC Bioinformatics, 15(1):166, doi:10.1186/1471-2105-15-166

**Relevant references**  

1) Wing-Cheong Wong, Sebastian Maurer-Stroh, Georg Schneider, Frank Eisenhaber, 2012,
*Transmembrane helix: simple or complex*
, Nucleic Acids Research (Web Server issue), doi:10.1093/nar/gks379  
  
2) Wing-Cheong Wong, Sebastian Maurer-Stroh, Frank Eisenhaber, 2011,
*Not all transmembrane helices are born equal: Towards the extension of the sequence homology concept to membrane proteins*
, Biology Direct, 6(57), doi:10.1186/1745-6150-6-57  
  
3) Wing-Cheong Wong, Sebastian Maurer-Stroh, Frank Eisenhaber, 2011,
*The Janus-faced E-values of HMMER2: Extreme value distribution or logistic function*
, Journal of Bioinformatics and Computational Biology, 9(1), doi:10.1142/S0219720011005264  
  
4) Wing-Cheong Wong, Sebastian Maurer-Stroh, Frank Eisenhaber, 2010,
*More than 1001 problems with protein domain databases: transmembrane regions, signalpeptides and the issue of sequence homology*
, PLoS Computational Biology, 6(7), doi:10.1371/journal.pcbi.1000867

**Contact**  

wongwc@bii.a-star.edu.sg

  

24-03-2015 17:37:38 Copyrights Bioinformatics Institute A\*STAR

  
